# Linkage and QTL mapping for tuber shape and specific gravity in a tetraploid mapping population of potato representing the russet market class

**DOI:** 10.1186/s12870-021-03265-2

**Published:** 2021-11-03

**Authors:** Jaebum Park, Alicia N. Massa, David Douches, Joseph Coombs, Deniz Akdemir, G. Craig Yencho, Jonathan L. Whitworth, Richard G. Novy

**Affiliations:** 1 USDA-ARS, Small Grains and Potato Germplasm Research Unit, Aberdeen, ID 83210 USA; 2 USDA-ARS, National Peanut Research Laboratory, Dawson, GA 39842 USA; 3 Michigan State University, East Lansing, MI 48824 USA; 4 University College Dublin, Belfield, Dublin 4, Ireland; 5 North Carolina State University, Raleigh, NC 27695 USA

**Keywords:** Quantitative trait locus/loci (QTL) analysis, Potato tuber shape, Potato specific gravity, MAPpoly, QTLpoly

## Abstract

**Background:**

Tuber shape and specific gravity (dry matter) are important agronomic traits in potato processing and impact production costs, quality, and consistency of the final processed food products such as French fries and potato chips. In this study, linkage and QTL mapping were performed for these two traits to allow for the implementation of marker-assisted selection to facilitate breeding efforts in the russet market class. Two parents, Rio Grande Russet (female) and Premier Russet (male) and their 205 F1 progenies were initially phenotyped for tuber shape and specific gravity in field trials conducted in Idaho and North Carolina in 2010 and 2011, with specific gravity also being measured in Minnesota in 2011. Progenies and parents were previously genotyped using the Illumina SolCAP Infinium 8303 Potato SNP array, with ClusterCall and MAPpoly (R-packages) subsequently used for autotetraploid SNP calling and linkage mapping in this study. The 12 complete linkage groups and phenotypic data were then imported into QTLpoly, an R-package designed for polyploid QTL analyses.

**Results:**

Significant QTL for tuber shape were detected on chromosomes 4, 7, and 10, with heritability estimates ranging from 0.09 to 0.36. Significant tuber shape QTL on chromosomes 4 and 7 were specific to Idaho and North Carolina environments, respectively, whereas the QTL on chromosome 10 was significant regardless of growing environment. Single marker analyses identified alleles in the parents associated with QTL on chromosomes 4, 7, and 10 that contributed to significant differences in tuber shape among progenies. Significant QTL were also identified for specific gravity on chromosomes 1 and 5 with heritability ranging from 0.12 to 0.21 and were reflected across environments.

**Conclusion:**

Fully automated linkage mapping and QTL analysis were conducted to identify significant QTL for tuber shape and dry matter in a tetraploid mapping population representing the russet market class. The findings are important for the development of molecular markers useful to potato breeders for marker-assisted selection for the long tuber shape and acceptable dry matter required by the potato industry within this important market class.

**Supplementary Information:**

The online version contains supplementary material available at 10.1186/s12870-021-03265-2.

## Background

Potato (*Solanum tuberosum*) is known as one of the four primary food sources worldwide [1]. According to the USDA National Agricultural Statistics Service [2], the United States produced about 19.2 million metric tons in 2019. Over 65% of the potato production in the U.S. is used for production of processed potato products, such as French fries, chips, and refrigerated and frozen items utilized by food services [2]. Tuber shape and specific gravity (primarily reflecting starch content) are two essential factors for potato processing. Specific gravity is associated with the amount of oil used during processing and final product quality [3]. Round and long tuber shapes are required by the chip-processing and French fry industries, respectively [4, 5]. Despite the high demand for processed potato products and the importance of these two traits, identification of quantitative trait locus (or loci) (QTL) associated with them, especially in the russet market class, have been limited. Identification of QTL associated with tuber shape and specific gravity could prove useful in marker assisted selection (MAS) to facilitate breeding.

Tuber shape is a polygenic trait of potato, with tubers showing a distribution from round to long [6–8]. As a result, multiple quantitative genetic studies have been performed for tuber shape using various types of mapping populations. A major locus, *Ro,* which is known to control tuber shape [7], has been mapped on chromosome 10 [4, 9, 10]. Furthermore, multiple potato research projects commonly reported QTL associated with tuber shape on chromosome 10 after analyzing full-sib diploid, F2, or gynogenic di-haploid populations [8, 11–14]. Other chromosomes also had QTL for tuber shape. For example, QTL for tuber shape were found on chromosome 2 by Bradshaw et al. [15], Hara-Skrzypiec et al. [8], Meijer et al. [13], and Prashar et al. [14]. Chromosome 3, 4, 5, and 11 had QTL for tuber shape and the regularity of tuber shape [8, 12, 14–16]. QTL for tuber shape were also found on chromosome 6 and 9 [12, 14]. A QTL for regularity of tuber shape was detected on chromosome 8 by Hara-Skrzypiec et al. [8].

Specific gravity is influenced by environmental effects such as temperature, rainfall, and day length [17]. It was confirmed that not only multiple genetic loci control specific gravity, but also genotype × environment interaction is a significant factor for this trait in both tetraploid and diploid potatoes [18, 19]. Freyre and Douches [20] performed a QTL analysis for specific gravity with a diploid mapping population in three different locations and mapped ten putative QTL on chromosomes 1, 2, 3, 5, 7, and 11. Schäfer-Pregl et al. [21] found multiple QTL associated with specific gravity on all 12 potato chromosomes after analyzing two populations derived from crosses between wild diploid potato species and di-haploid lines. Li et al. [22] also reported loci linked to this trait on chromosomes 2, 3, 5, 7, 8, 9, 10, and 11 through candidate genes- or known loci-association mapping in tetraploid potato populations. Li et al. [23] identified specific gravity QTL on chromosomes 1, 2, and 8 after analyzing a Chinese tetraploid mapping population at three different locations for 2 years. Schönhals et al. [24] also observed several loci linked to tuber starch content, which can be interpreted as a characteristic of specific gravity, on chromosome 1, 3, 4, 8, 9, 10, 11, and 12.

In this study, we conducted a QTL analysis for tuber shape and specific gravity with an autotetraploid (2n = 4x = 48) F_1_ mapping population representing the russet-skinned market class which is the primary class grown in North America and which is also characterized by long tuber shape. The F_1_ population was previously used for studying tuber sugar concentration, processing quality and maturity related traits [25, 26]. In this study, the same mapping population was utilized for QTL analyses of tuber shape and specific gravity after increasing its progeny number from 162 to 205 to develop improved linkage groups and QTL maps. Based on the QTL analysis with multiple locations and years, the loci harboring positive and negative impact alleles were localized. New R packages, which can automatically develop the 12 tetraploid linkage groups and QTL maps, were introduced in the current study to improve efficiencies.

## Methods

### Plant material

The autotetraploid mapping population A05141, consisting of 205 F1 progeny, was obtained from a cross between two North American cultivars ‘Rio Grande Russet’ (female parent) and ‘Premier Russet’ (male parent) [26] conducted at the USDA-ARS Small Grains and Potato Germplasm Research Unit (Aberdeen, ID). Rio Grande Russet was initially released as a high-quality fresh market russet with high yield [27]. Premier Russet, which is resistant to reducing sugar accumulation after long-term cold storage, was appropriate for both processing and fresh market [28]. Rio Grande and Premier Russets typically have long and oblong shapes, respectively. Specific gravity of the parents Rio Grande Russet and Premier Russet were 1.075 and 1.079, respectively, when averaged over years and locations.

### Tuber shape and specific gravity measurement

A randomized complete block design with two replications of ten-hill plots was used for assessing the two parents and their progeny at each location [26]. All details of the field experimentation were described in Massa et al. [26]. The A05141 population including the two parents were phenotyped for tuber shape and specific gravity in field trials conducted in Idaho and North Carolina in 2010 and 2011, with specific gravity, also being measured in Minnesota in 2011. Tuber shape was visually scored from “1” (Compressed) to “5” (Long) (Supplementary Fig. 1) [29] based on a scale developed originally by the NE1014 Multi-State Research Project. The specific gravity was calculated based on weight in air / (weight in air – weight in water).

### Best linear unbiased predictors (BLUPs) analyses for tuber shape and specific gravity

The phenotypic data for each of the traits were analyzed with the following mixed-effects model to get estimates of variance components and also for prediction of the genetic values for the genotypes [30–32]:

1 $${y}_{ijkl}=\mu +{G}_i+{R}_{j(kl)}+{Y}_k+{L}_l+{(GY)}_{ik}+{(GL)}_{il}+{(YL)}_{kl}+{\left( GY L\right)}_{ik l}+{\upvarepsilon}_{ijkl}$$

In eq. (1), *y*_*ijkl*_ is the phenotype for genotype *i* in replication *j* of year *k* and location *l*. *μ* is the population mean, *G*_*i*_ is the random effect of genotype *i*, *R*_*j*_ is the random effect of replication *j* within an environment, *Y*_*k*_ is the fixed effect of year *k*, *L*_*l*_ is the random effect of location *l*, (*GY*)_*ik*_ is the genotype *i* by year *k* interaction, (*GL*)_*il*_ is the genotype *i* by location *l* interaction, (*YL*)_*kl*_ is the year *k* by location *l* interaction, (*GYL*)_*ikl*_ is the genotype *i* by year *k* by location *l* interaction, and *Ɛ*_*ijkl*_ is the residual error. Each random effect is assumed to be independent from the rest of the random effects and have a normal distribution with mean zero. The predictions for the random genotype effects (BLUPs) were used in the subsequent QTL analyses [33]. Distributions of all the BLUP datasets were visually evaluated to check their normality through histograms.

### Statistics for heritability

Broad-sense heritability of tuber shape and specific gravity was calculated using the following equations [34].

2 $${H}^2=\kern0.5em \frac{\upsigma_g^2}{\upsigma_p^2}$$

3 $${\upsigma}_p^2={\upsigma}_g^2+\frac{\upsigma_{gy}^2}{y}+\frac{\upsigma_{gl}^2}{l}+\frac{\upsigma_{gy l}^2}{y\cdot l}+\frac{\upsigma_{\varepsilon}^2}{y\cdot l\cdot \beta }$$

In eq. (2), ${\sigma}_p^2$ stands for the variance of mean phenotypic measurements across replicates.

In eq. (3), the variances of *G*_*i*_, *R*_*j*_, *L*_*l*_, (*GY*)_*ik*_, (*GL*)_*il*_, (*YL*)_*kl*_, (*GYL*)_*ikl*_, and *Ɛ*_*ijkl*_ are denoted by ${\sigma}_g^2$, ${\sigma}_r^2$, ${\sigma}_{gy}^2$, ${\sigma}_{gl}^2$, ${\sigma}_{gyl}^2$, and ${\sigma}_{\varepsilon}^2$ correspondingly. The terms *y, l,* and *β* used in eq. (3) indicate the number of years, locations and replications, respectively. All the statistical analyses discussed here were conducted by JMP Pro® Statistics, Version 12 (SAS Institute Inc., Cary, NC, USA).

### Genotyping, SNP calling and dosage evaluation

The DNA sample quality evaluation, genotyping, and obtainment of SNP theta scores were conducted through the Illumina Infinium SolCAP SNP array (8303 SNPs), the Illumina iScan system, and GenomeStudio software (Illumina, Inc., San Diego, CA) as described in Massa et al. [26], Park et al. [35], and Staaf et al. [36]. The SNP theta values were then translated into autotetraploid marker genotypes (AAAA, AAAB, AABB, ABBB, and BBBB) by R-package, ClusterCall (version 1.5) [37].

### Construction of linkage groups and QTL maps

The R-package MAPpoly (v. 0.1.0), which can analyze ploidy levels up to eight when using hidden Markov models (HMM), was used to develop linkage groups in this study [38–40]. Once the translated SNP marker data were imported into MAPpoly, the *filter_missing, filter_segregation, make_seq_mappoly,* and *elim.redundant* functions were used to filter out uninformative markers. MAPpoly then calculated two-point recombination fractions between all the imported SNP markers, sorting the most legitimate phase between each marker pair. The two-point analysis estimates the recombination frequency, the likelihood, and the LOD score for each pair of markers, providing all possible phases using the most likely parental genotype. The phases for each marker pair were then sorted based on their likelihood [41]. Unweighted Pair Group Method with Arithmetic Mean (UPGMA) hierarchical clustering method was used to group the selected markers into 12 linkage groups. The markers belonging to each linkage group were first arranged in order through multidimensional scaling (MDS) operated by an R-package, MDSMap [42]. The arranged markers were then locally re-ordered for refinement based on the potato reference genome PGSC Version 4.03 [43, 44]. This whole linkage mapping process was automated by MAPpoly R-package [45].

QTLpoly, an R-package designed for QTL analysis of polyploid organisms, was used to combine the 12 linkage groups with the BLUP datasets estimated from the phenotype data, and then to draw 12 QTL maps as described in da Silva Pereira et al. [45]. In brief, a random-effect multiple interval mapping (REMIM) model, using the *remim* function as implemented in QTLpoly, was used to fit various random-effect QTL by evaluating a single parameter per QTL. The software then performed linear score statistics tests [46] at every position and compared its *p*-value to a prescribed critical value. The *p*-values showed a continuous pattern over the whole range of the unit interval as a result of weighted sums of the scores from the profiled likelihood. If only one QTL exists in the model, the test is nonasymptotic or called “exact.” On the other hand, if there are two or more QTL, a moment-based approximation to the null distribution is used [45, 46]. The QTLpoly then takes the *p*-values resulting in *LOP* scores (*LOP* = − log10 (*p*-value)), which is used for intuitive visualization and comparison of the detected QTL and for calculation of support intervals of the QTL [45]. The QTL with three or higher *LOP* scores were adopted as significant QTL peaks in this study to reflect and not discard several QTL that were near the more typically used four or higher *LOP* score [G. da Silva Pereira, pers. comm.]. Subsequently additional validation methods were used to protect from false-positive QTL with the use of the three or high LOP which included checking the consistency of a QTL across locations and years. Furthermore, allele effect and single-marker analyses were conducted to check how the presence or absence of a target allele affected the phenotype data of the two traits. Detailed information on those validation processes will be explained in the following paragraphs. The QTLpoly (R-package) also provides information on support intervals defined as the QTL peak adjacent to zone with *LOP* higher than or equal to *LOP – d*, where *d* is a constant, which subtracts the highest *LOP* in that region [45, 47]. We used approximately 95% support intervals by using *LOP –* 1.5*.* The *fit_model* function in QTLpoly calculated the heritability of the significant QTL. They were labeled as “*h*^*2*^_*QTL*_*”* in the current study. If a *h*^*2*^_*QTL*_ of a QTL peak is higher than 10%, the QTL will be considered as a major QTL, while a *h*^*2*^_*QTL*_ ≤ 10% was considered a minor QTL [45]. After significant QTL were determined, the closest SNPs to the QTL were deeply investigated.

### Analyses of allele effects

The QTLpoly software provided allele effects at each SNP position, which are indicated by bar graphs (Supplementary Fig. 2). These effects reflect the contribution of the two parents to the mean of the whole mapping population. For example, how much each homolog of the four homologs of the two parents adds to or subtracts from the mean given one of 205 observed genotypes [45]. The X-axis of an allele effect graph indicates the four homologs of both parents. For example, “a” to “d” represent the four homologs of Rio Grande Russet, and “e” to “h” represent the four homologs of Premier Russet (Supplementary Fig. 2). The Y-axis displays the quantity of an allele effect on each homolog (Supplementary Fig. 2). It is possible to infer which parent primarily contributes to the disposition of the tuber shape and specific gravity of their progenies through these graphs. The graphs also helped to confirm each allele effect vector, such as the quantity of either positive (= increase in) or negative (= decrease in) effect among each parent’s four homologs. Both the positive and negative allele effects on each homolog were then converted to absolute values. The sum of all the eight absolute values at each mapped locus was used to compare contributions of the mapped QTL. The amount of the four absolute values of each parent was calculated to see the contribution of each parent [G. da Silva Pereira, unpublished].

### Single-marker analyses by investigating BLUP segregation depending on genotype

After checking the allele effect of the QTL, single-marker analysis was pursued to check whether the allele effects were actually reflected in the original phenotype data or not, as well as to find the most fitted genetic models such as additive, simplex-dominant, etc. When a target QTL and the linked SNP marker were selected, we separated BLUP data by genotype, giving us two to five different groups. We then compared averages of the BLUPs of each genotype group to check whether a significant mean difference existed between two genotype groups or not. The presence of the significant mean difference can indirectly reveal allele effects on phenotype. For instance, if the “B” allele of an SNP marker is associated with an increase in specific gravity and has an additive impact, the greater number of B alleles in a genotype would be expected to confer a higher specific gravity. Tukey-Kramer mean comparison test (JMP Pro® Statistics, Version 12; SAS Institute Inc., Cary, NC, USA) was used for the single-marker analysis (*p*-value < 0.05).

## Results

### Marker selection and linkage group construction

Two hundred five individuals of the A05141 mapping population and their parents, Rio Grande Russet and Premier Russet, were genotyped with the SolCAP Infinium 8303 potato SNP array. Illumina GenomeStudio software was used to analyze the array data and calculate theta value scores of each individual for 8303 SNP loci. ClusterCall uses the theta values to determine 5630 polyploid marker genotypes. Since MAPpoly cannot analyze the SNPs having no-call in either the two parents, 141 SNP markers were removed (Supplementary Table 1). Within MAPpoly, the *filter_missing* function filtered out 1724 markers with 20% or more no-calls. The *filter_segregation* function then performed the chi-squared (χ2) test, which matches expected genotype frequencies against observed frequencies and calculates the associated *p*-value. Bonferroni correction was used to distinguish informative markers (*p*-value < 0.05). The *make_seq_mappoly* argument omitted additional 171 markers, which significantly did not meet the expected segregation ratios based on Mendelian inheritance. The “*elim.redundant”* argument automatically identified and removed 215 redundant markers. During the two-point and MDS processes, 1020 markers were additionally omitted, which were uninformative, co-segregating, or not belonging to one of the 12 linkage groups.

**Fig. 1:**
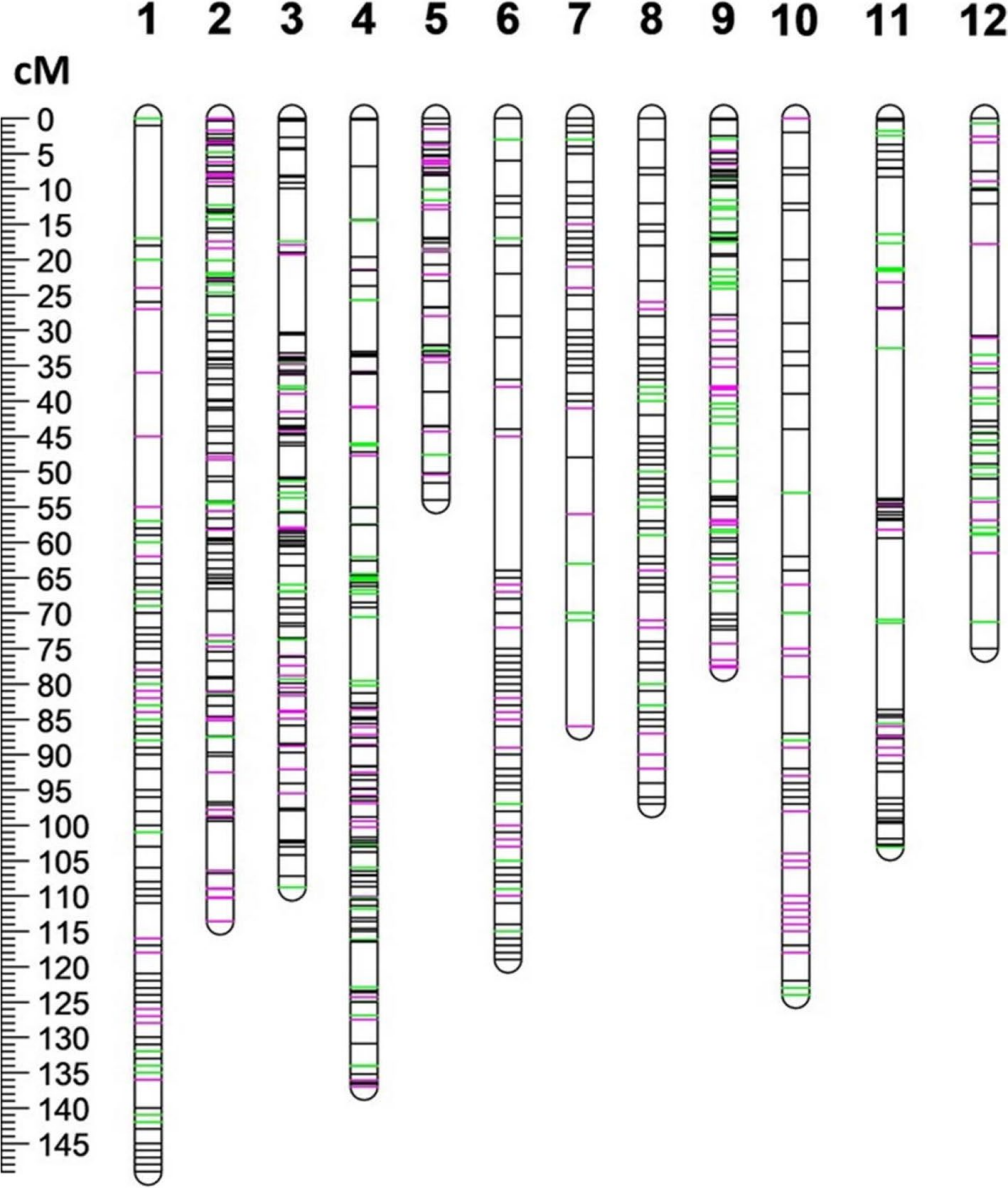
Distribution of the 2359 SNP markers employed for QTL mapping in this study. The 12 numbers at the top represent potato chromosomes. SNPs unique to Rio Grande Russet are shown with green lines, those unique to Premier Russet are shown with pink lines, and those shared between the two parents are shown with black lines. The scale bar on the left indicates genetic distance in centiMorgans (cM)

A total of 2359 SNPs were subsequently used for constructing the final 12 linkage groups, which represent the base potato chromosome number. The number of the selected SNP markers per chromosome ranged from 290 on chromosome 1 to 100 on chromosome 12 (Table 1). The length of each linkage group varied from 149 cM for chromosome 1 to 54 cM for chromosome 5 (Fig. 1; Table 1). Among the selected markers, 1786 and 1974 SNPs segregated in Rio Grande Russet and Premier Russet, respectively, while 1365 SNPs segregated in both parents (Fig.1; Table 1). Rio Grande Russet and Premier Russet had 1223.5 and 1242.2 cM genetic map lengths, respectively, and they covered 94% of the potato physical map (Table 1). High concordance was observed between SNP marker positions of the linkage and PGSC version 4.03 physical maps since MAPpoly used the physical maps for refinement of the linkage groups. The average distance between contiguous SNPs was 0.69 and 0.63 cM for Rio Grande Russet and Premier Russet. The 12 complete autotetraploid linkage groups for each parent were visualized in Supplementary Fig. 3.

**Table 1:** Linkage group summary for the two parents: Rio Grande Russet and Premier Russet

	No. Mapped SNPs ^a^			Map Length (cM) ^b^		Map Coverage ^c^	
Chr ^d^	Total	Rio Grande Russet	Premier Russet	Rio Grande Russet	Premier Russet	Rio Grande Russet	Premier Russet
1	290	237	246	149	149	0.98	0.98
2	263	184	219	109	114	0.75	0.76
3	241	185	241	109	109	0.97	0.97
4	271	209	199	137	137	1.00	0.97
5	124	81	109	54	54	0.94	0.93
6	241	180	204	119	119	1.00	1.00
7	104	79	96	71	86	0.91	0.88
8	233	182	212	97	97	0.98	0.98
9	204	157	150	77	78	0.91	0.92
10	139	89	124	124	122	0.96	0.95
11	149	126	111	103.1	103.1	0.93	0.94
12	100	77	63	75	75	0.92	0.91
Total	2359	1786	1974	1224	1242	0.94	0.94

^a^The number of mapped single nucleotide polymorphisms

^b^Linkage group lengths in centiMorgans

^c^Map coverage relative to PGSC Version 4.03 pseudomolecules

^d^Chromosome number

### Statistical analyses of phenotype data & heritability

#### Tuber shape

The tuber shape score “one”, representative of a compressed shape (Supplementary Fig. 1) was not observed across the two locations over 2 years, so that the A05141 progeny ranged from two to five for tuber shape ratings (Supplementary Fig. 4; Supplementary Table 2). The mixed model (1) was used to analyze the raw tuber shape phenotype data, and to convert the raw data to BLUP values. Variance component estimates of tuber shape were organized in Table 2. Interestingly, the variance of location effect was the largest among the several other random effects, explaining the significant association between tuber shape and features of each location. On the other hand, the variance of the year × location effect was essentially zero. The broad-sense heritability of the tuber shape was 0.57.

**Table 2:** Variance component estimates of tuber shape and specific gravity

Tuber Shape			Specific Gravity		
Random Effect	Var ^a^ Component	Std Error ^b^	Random Effect	Var ^a^ Component	Std Error ^b^
clone ^c^	0.1699089	0.0367514	clone	2.02E-05	6.50E-06
location	0.2792218	0.4001046	location	6.10E-05	1.01E-04
rep [year,location]	0.008989	0.0068642	rep [year,location]	8.00E-07	1.30E-06
clone*year	0.0031053	0.0188942	clone*year	0	0
clone*location	0.138096	0.0311013	clone*location	2.20E-05	9.20E-06
year*location	0	0	year*location	6.64E-05	7.97E-05
clone*year*location	0.0713052	0.0269068	clone*year*location	1.07E-05	1.15E-05
Residual	0.3023925	0.0168895	Residual	2.27E-04	1.14E-05
Total	0.9730186	0.4018347	Total	4.08E-04	1.10E-04
Fixed Effect	Estimate	Std Error ^b^	Fixed Effect	Estimate	Std Error ^b^
Intercept	3.9237789	0.377372	Intercept	1.0772061	0.0062750
year[2010]	0.0153177	0.038452	year[2010]	−0.0015120	0.0043460

^a^Variance component

^b^Standard error

^c^“Clone” indicates a genetic effect of a clone

Nine different BLUP datasets were derived, depending on the combination of BLUP effects of each clone with respect to tuber shape. For example, the first dataset, “*TS_clo,*” was composed of the BLUPs of pooled phenotypic data across all the two-years and two locations, with the “TS” being an abbreviation for tuber shape. The second set, “*TS_clo_ID*” had the BLUPs of interaction between clone and Idaho location. Likewise, the third to ninth BLUP datasets had different combinations of the effects. Although the total progeny number used for genetic mapping was 205, each BLUP dataset has less than 205 BLUPs and different numbers of BLUPs because several clones in this previously unselected population had poor field emergence or did not grow well during the growing seasons. The description of the nine BLUP datasets was summarized in Supplementary Table 3. Distribution patterns of the nine BLUP datasets approximated normal distributions with the exception being *TS clo ID* with some skewness in distribution observed (Supplementary Fig. 5).

#### Specific gravity

The specific gravity of the A05141 progeny was segregated in all the environments, including the three locations and 2 years (Supplementary Table 2). In the same manner as with tuber shape, the raw specific gravity data was converted to BLUPs based on the mixed model (1). Table 2 summarized variance component estimates of specific gravity. Variances of location and year × location effects were significantly higher than other random effects, reflecting the high impact of location effect. The variance of clone × year was zero (Table 2), resulting in the absence of the BLUP datasets of the interactions between clone and year (e.g., *SG_clo_2010* and *SG_clo_2011*) (Supplementary Table 3), with “*SG*” indicating specific gravity. In total, nine BLUP datasets were obtained. All nine of the specific gravity BLUP datasets were normal or close to a normal distribution (Supplementary Fig. 5). As introduced above, the titles of each BLUP dataset are named after the combination of BLUP components. The broad-sense heritability of the specific gravity was 0.42.

### QTL for tuber shape and specific gravity

**Fig. 2:**
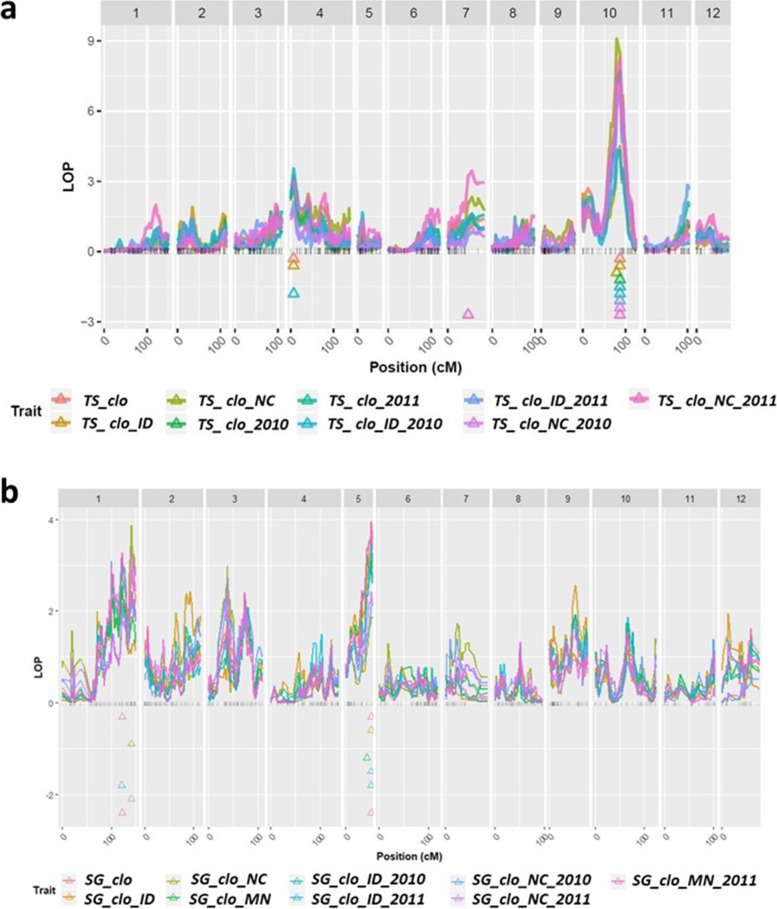
QTL maps for Tuber Shape and Specific Gravity. **a**. Tuber Shape. **b**. Specific Gravity. BLUP data abbreviations: tuber shape (*TS*), specific gravity (*SG*), a genetic effect of clones (*clo*), Idaho (*ID*), North Carolina (*NC*), Minnesota (*MN*) location effects, 2010 (*2010*), and 2011 (*2011*) year effects; Locations of the significant QTL peaks were marked by triangles. Y axis represents LOP score, which is equal to – log10 (*p*-value)

**Fig. 3:**
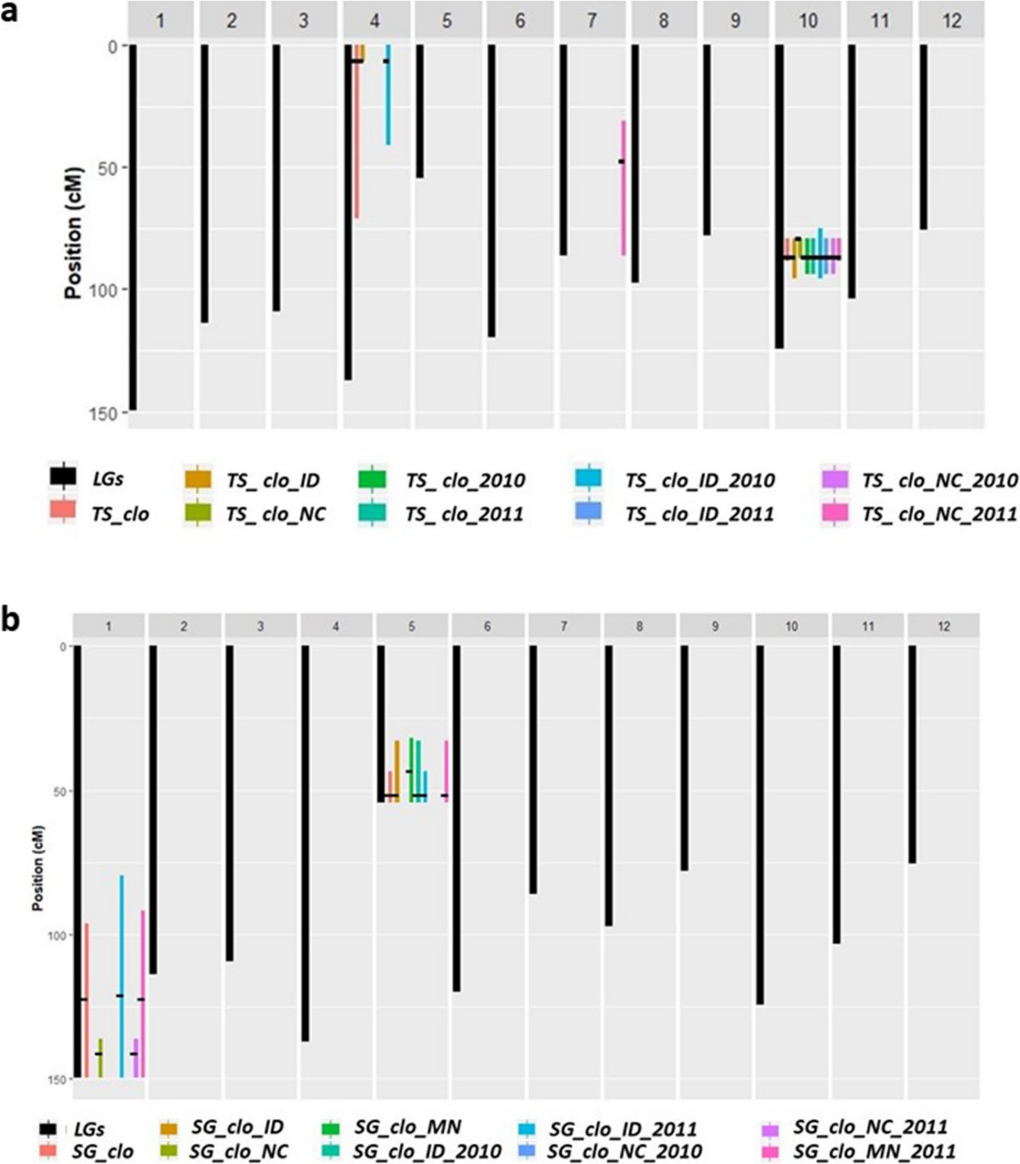
Location of significant QTL peaks and their support intervals. **a**. Tuber Shape. **b**. Specific Gravity. The X axes in Fig.3a and b represent 12 different potato chromosomes. The black bars indicate lengths of each chromosome, respectively. The color bars indicate lengths of each support interval. Those support intervals were labeled in the same way described in Fig. 2

After obtaining the BLUP datasets (Supplementary Table 3), the 12 complete linkage groups and the BLUPs were imported into QTLpoly. Through the *remim* function, QTL mapping was automatically performed by the computer, resulting in QTL maps, detailed information on the mapped QTL such as chromosome number, LOP score, location, support intervals, *h*^*2*^_*QTL*_, and closest SNP markers to the mapped QTL (Figs. 2 and 3; Table 3). By using the argument, *qtl_effects* in QTLpoly, it was possible to investigate allele effects of the SNP markers where the significant QTL are located (Supplementary Fig. 2). A total of 13 and 11 QTL were reported for tuber shape and specific gravity, respectively (Table 3). The positions of the mapped QTL for tuber shape in this study were compared with locations of tuber shape associated QTL reported by references in Table 4.

**Table 3:** Summary of QTL for tuber shape (TS) and specific gravity (SG)

Labels of QTL	Traits ^a^	chr ^b^	LOP score	Heritability of mapped QTL (*h*^*2*^_*QTL*_)	QTL Position (Support Interval) [Unit: cM] ^c^	Most closely associated SNPs ^d^
***TS_QTL_ch04_a***	*TS_clo*	4	3.11	0.09	**6.78** (0.00–70.48)	c2_54790
***TS_QTL_ch04_b***	*TS_clo_ID*	4	3.27	0.11	**6.78** (0.00–6.78)	c2_54790
***TS_QTL_ch04_c***	*TS_clo_ID_2010*	4	3.54	0.12	**6.78** (0.00–40.75)	c2_54790
***TS_QTL_ch07_a***	*TS_clo_NC_2011*	7	3.25	0.11	**47.66** (31.23–85.71)	c2_26012
***TS_QTL_ch10_a***	*TS_clo*	10	7.10	0.28	**86.75** (79.09–88.09)	c1_15594
***TS_QTL_ch10_b***	*TS_clo_ID*	10	4.50	0.18	**86.75** (79.09–95.37)	c1_15594
***TS_QTL_ch10_c***	*TS_clo_NC*	10	9.08	0.36	**79.09** (79.09–86.75)	c1_11535
***TS_QTL_ch10_d***	*TS_clo_2010*	10	7.70	0.32	**86.75** (79.09–93.38)	c1_15594
***TS_QTL_ch10_e***	*TS_clo_2011*	10	7.64	0.34	**86.75** (79.09–93.38)	c1_15594
***TS_QTL_ch10_f***	*TS_clo_ID_2010*	10	4.31	0.19	**86.75** (74.94–95.37)	c1_15594
***TS_QTL_ch10_g***	*TS_clo_ID_2011*	10	7.07	0.31	**86.75** (79.09–93.38)	c1_15594
***TS_QTL_ch10_h***	*TS_clo_NC_2010*	10	7.23	0.31	**86.75** (79.09–93.38)	c1_15594
***TS_QTL_ch10_i***	*TS_clo_NC_2011*	10	8.24	0.33	**86.75** (79.09–88.09)	c1_15594
***SG_QTL_ch01_a***	*SG_clo*	1	3.19	0.12	**122.30** (96.30–149.24)	c1_1847
***SG_QTL_ch01_b***	*SG_clo_NC*	1	3.88	0.19	**141.26** (136.13–149.24)	c2_49905
***SG_QTL_ch01_c***	*SG_clo_ID_2011*	1	3.15	0.13	**121.23** (79.47–149.24)	c2_7053
***SG_QTL_ch01_d***	*SG_clo_NC_2011*	1	3.32	0.16	**141.26** (136.13–149.24)	c2_49905
***SG_QTL_ch01_e***	*SG_clo_MN_2011*	1	3.27	0.14	**122.30** (91.69–149.24)	c1_1847
***SG_QTL_ch05_a***	*SG_clo*	5	3.94	0.21	**51.63** (43.55–54.04)	c2_3452
***SG_QTL_ch05_b***	*SG_clo_ID*	5	3.61	0.18	**51.63** (33.00–54.04)	c2_3452
***SG_QTL_ch05_c***	*SG_clo_MN*	5	3.24	0.15	**43.55** (32.00–54.04)	c2_42406
***SG_QTL_ch05_d***	*SG_clo_ID_2010*	5	3.05	0.17	**51.63** (33.00–54.04)	c2_3452
***SG_QTL_ch05_e***	*SG_clo_ID_2011*	5	3.47	0.18	**51.63** (43.55–54.04)	c2_3452
***SG_QTL_ch05_f***	*SG_clo_MN_2011*	5	3.92	0.20	**51.63** (33.00–54.04)	c2_3452

^a^The same BLUP datasets described in Supplementary Table 3

^b^Chromosome numbers

^c^The bold figures indicate the locations of the mapped QTL peak and numbers in the parentheses showing ranges of their support intervals; The unit is centiMorgans (cM)

^d^The most adjacent SNPs to each QTL peak were presented in this column; “solcap_snp_” was omitted at the beginning of all the SNP marker names

**Table 4:** Location comparison of QTL and linked SNPs between the A05141 mapping population and previously published references on molecular markers associated with potato tuber shape

QTL ^a^ (Traits)	ch	SNPs ^b^	Phy. Map. Pos. ^c^	Molecular markers from references ^d^	References	Reference Marker phy. Map. Pos. ^e^	Dist. Bet. our SNP and reference SNP (cM) ^f^
***TS_QTL_ch04_a*** ***(TS_clo)*** ***TS_QTL_ch04_b*** ***(TS_clo_ID)*** ***TS_QTL_ch04_c*** ***(TS_clo_ID_2010)***	4	^a^c2_54790	1,151,453	pPt-651,535	Hara-Skrzypiec et al. 2018	~ 8,825,900	7.67
***TS_QTL_ch10_a*** ***(TS_clo)*** ***TS_QTL_ch10_b*** ***(TS_clo_ID)*** ***TS_QTL_ch10_d*** ***(TS_clo_2010)*** ***TS_QTL_ch10_e*** ***(TS_clo_2011)*** ***TS_QTL_ch10_f*** ***(TS_clo_ID_2010)*** ***TS_QTL_ch10_g*** ***(TS_clo_ID_2011)*** ***TS_QTL_ch10_h*** ***(TS_clo_NC_2010)*** ***TS_QTL_ch10_i*** ***(TS_clo_NC_2011)***	10	^a^c1_15594	50,187,264	^a^c2_27831	A. Massa, pers. comm.	50,806,407	0.62
				pPt-559,534	Hara-Skrzypiec et al. 2018	~ 49,356,600	0.83
				^a^c2_45606	Endelman & Jansky 2016	48,218,820	1.97
				^a^c2_25485	Endelman & Jansky 2016	48,737,840	1.45
				^a^c1_16351	Endelman & Jansky 2016	48,761,642	1.43
				^a^c1_8020	Endelman & Jansky 2016	48,863,048	1.32
				^a^c1_11540	Endelman & Jansky 2016	49,659,510	0.53
				^a^c2_27795	Endelman & Jansky 2016	50,458,044	0.27
				^a^c2_27821	Endelman & Jansky 2016	50,649,574	0.46
				^a^c2_27829	Endelman & Jansky 2016	50,782,097	0.59
				^a^c2_53946	Endelman & Jansky 2016	51,926,830	1.74
***TS_QTL_ch10_c*** ***(TS_clo_NC)***	10	^a^c1_11535	49,553,136	^a^c2_27831	A. Massa, pers. comm.	50,806,407	1.25
				pPt-559,534	Hara-Skrzypiec et al. 2018	~ 49,356,600	0.20
				^a^c2_45606	Endelman & Jansky 2016	48,218,820	1.33
				^a^c2_25485	Endelman & Jansky 2016	48,737,840	0.82
				^a^c1_16351	Endelman & Jansky 2016	48,761,642	0.79
				^a^c1_8020	Endelman & Jansky 2016	48,863,048	0.69
				^a^c1_11540	Endelman & Jansky 2016	49,659,510	0.11
				^a^c2_27795	Endelman & Jansky 2016	50,458,044	0.90
				^a^c2_27821	Endelman & Jansky 2016	50,649,574	1.10
				^a^c2_27829	Endelman & Jansky 2016	50,782,097	1.23
				^a^c2_53946	Endelman & Jansky 2016	51,926,830	2.37

^a^The titles of the mapped QTL and the BLUP data sets used

^b^The SNPs where the significant QTL peaks are located

^c^Physical map position of the SNPs above; Physical map position of each SNP was obtained by SPUD database

^d^SNPs or other diversity array technology (DArT) markers analyzed by references; Those markers are linked to tuber shape and eye depth

^e^The physical map position of the SNP and DArT markers analyzed by references

^f^The distance between SNPs identified in this study and other SNPs (or DArT markers) studied in the references

^a^ “solcap_snp_” was omitted at the beginning of all the SNP marker names

#### QTL for tuber shape

Significant QTL associated with tuber shape were detected on chromosome 4, 7, and 10 (Fig. 2a; Table 3). All the support intervals of the significant QTL were provided in Fig. 3a.

The significant QTL peak near 86.75 cM of chromosome 10 consistently appeared across two locations and years. Even though the *TS_clo_NC* BLUP dataset had its most significant QTL at 79.09 cM, the LOP score at the position, 86.75 cM, was also much higher than the threshold, proving the significance of the QTL at 86.75 cM (Fig. 2a; Table 3). The LOP scores of the nine QTL ranged from 4.31 to 9.08, and all their *h*^*2*^_*QTL*_ ranged from 18 to 36%. Except for the SNP marker solcap_snp_c1_11535, which was closely associated with SNP *TS_QTL_ch10_c*, the remaining eight QTL on chromosome 10 were most closely aligned with SNP solcap_snp_c1_15594 (Table 3).

One significant QTL peak was repeatedly detected at 6.78 cM on chromosome 4 across the three BLUP datasets, *TS_clo, TS_clo_ID*, and *TS_clo_ID_2010* (Fig. 2a; Table 3). The LOP scores of them ranged from 3.11 to 3.54, and their *h*^*2*^_*QTL*_ ranged from 0.09 to 0.12. The closest SNP marker was solcap_snp_c2_54790.

Chromosome 7 also harbored a major QTL (LOP = 3.25; *h*^*2*^_*QTL*_ = 11%) at 47.66 cM (Fig. 2a; Table 3) when the *TS_clo_NC_2011* BLUP dataset was analyzed. The closest SNP was solcap_snp_c2_26012. Allele effects of the SNPs of the mapped QTL were shown in Supplementary Fig. 2a. All detailed figure values for each allele effect were arranged in Supplementary Table 4.

#### QTL for specific gravity

Eleven significant QTL for specific gravity appeared on chromosomes 1 and 5 (Fig. 2b; Table 3). Their support intervals were reported in Fig. 3b and Table 3. Interestingly, all QTL had a major effect on specific gravity because their *h*^*2*^_*QTL*_ ranged from 12 to 21%.

Chromosome 1 showed five significant QTL. Three QTL, *SG_QTL_chr01_a, _c*, and _*e,* were located near 122 cM, with the remaining two, *SG_QTL_chr01_b* and *_d,* observed at 141.26 cM (Fig. 2b; Table 3). A total of three SNPs, solcap_snp_c1_1847, solcap_snp_c2_49905, and solcap_snp_c2_7053, were detected near the five QTL (Table 3). The LOP scores the QTL ranged from 3.15 to 3.88 (Fig. 2b). Their *h*^*2*^_*QTL*_ were between 12 and 19% (Table 3).

Five QTL at 51.63 cM on chromosome 5 were consistently detected from the five different BLUP datasets, *SG_clo*, *SG_clo_ID*, *SG_clo_ID_2010*, *SG_clo_ID_2011*, and *SG_clo_MN_2011*. In the *SG_clo_MN* BLUP dataset, *SG_QTL_ch05_c* was located at 43.55 cM (Fig. 2b; Table 3). The LOP scores of the QTL were between 3.05 and 3.94. The *h*^*2*^_*QTL*_ of the six QTL ranged from 15 to 21%. SNP solcap_snp_c2_42406 was closely linked to *SG_QTL_ch05_c* with the remaining five QTL aligned with solcap_snp_c2_3452 (Table 3). Supplementary Fig. 2b displayed allele effects of the SNPs linked to the six mapped QTL, and Supplementary Table 4 organized each allele effect value.

### Allele effects of the mapped QTL

#### Allele effect analyses of tuber shape QTL

Among the 104 allele effects (four homologs × two parents × 13 QTL), 51 positive and 53 negative allele effects were observed (Supplementary Fig. 2a; Supplementary Table 4). When the absolute values of the two parents’ allele effects were investigated, Rio Grande Russet and Premier Russet provided 1.77 (40%) and 2.61 (60%) contributions to tuber shape. The contribution of Rio Grande Russet was greater at *TS_QTL_ch04_a* (54%) and *TS_QTL_ch07_a* (51%). The influence of Premier Russet was greater at *TS_QTL_ch04_b* (53%), *TS_QTL_ch04_c* (53%), *TS_QTL_ch04_a* (54%), *TS_QTL_ch10_a* (61%), *TS_QTL_ch10_b* (55%), *TS_QTL_ch10_c* (70%), *TS_QTL_ch10_d* (61%), *TS_QTL_ch10_e* (63%), *TS_QTL_ch10_f* (64%), *TS_QTL_ch10_g* (55%), *TS_QTL_ch10_h* (62%), and *TS_QTL_ch10_i* (64%) (Supplementary Table 4). The sums of the eight absolute allele effects of the nine QTL on chromosome 10 (*TS_QTL_ch10_a* to_*i*) were 0.33, 0.39, 0.51, 0.36, 0.38, 0.28, 0.44, 0.39, and 0.42, respectively (Supplementary Table 4). Those of the three QTL on chromosome 4 (*TS_QTL_ch04_a* to *_c*) were 0.16, 0.29, and 0.21, respectively. Finally, the *TS_QTL_ch07_a* showed 0.22 (Supplementary Table 4).

#### Allele effect analyses of specific gravity QTL

While investigating the 88 allele effects for specific gravity (four homologs × two parents × 11 QTL), 22 positive and 22 negative effects were observed in Rio Grande Russet as well as 29 positive, and 15 negative effects were detected in Premier Russet. Rio Grande Russet and Premier Russet’s contribution for specific gravity were 0.0155 (58%) and 0.0113 (42%), respectively (Supplementary Fig. 2b; Supplementary Table 4). When contributions of Rio Grande Russet and Premier Russet for the 11 specific gravity QTL were compared, the influence of Rio Grande Russet was persistently stronger than Premier Russet (Supplementary Table 4). The sums of absolute values of the eight allele effects of the five QTL on chromosome 1 (*SG_QTL_ch01_a* to_*e*) were 0.0019, 0.0043, 0.0019, 0.0026, and 0.0022, respectively (Supplementary Table 4). Those of the six QTL on chromosome 5 (*SG_QTL_ch05_a* to *_f*) were 0.0023, 0.0026, 0.0021, 0.0021, 0.0022, and 0.0025, respectively (Supplementary Table 4).

### Single-marker analyses by investigating BLUP segregation depending on genotype

Single-marker analysis was performed to indirectly monitor changes in the two traits due to the presence (or absence) of an allele of the SNPs linked to a QTL. Significant mean differences between genotype groups were detected for SNP markers, solcap_snp_c2_54790, solcap_snp_c2_26012, and solcap_snp_c1_11535 for tuber shape, and solcap_snp_c2_49905, solcap_snp_c2_3452, and solcap_snp_c2_42406 for specific gravity (Supplementary Fig. 6).

## Discussion

### Potential feasibility of fully automated linkage and QTL mapping of tetraploid potatoes

Until the early 2000’s, QTL analyses of tetraploid mapping populations of potato had not been extensively conducted relative to diploid potatoes because of a lack of both adequate informative molecular markers and the necessary high-performance software required for tetraploid QTL analysis [35]. About a decade ago, the development and improvement of potato SNP arrays and suitable software such as TetraploidSNPMap (TPMSNP) [48] encouraged potato researchers to directly implement QTL analysis with tetraploid mapping populations of potato [12, 26, 33, 35, 42, 44, 48–53]. Although TPMSNP opened a new chapter in tetraploid QTL analysis by using SNP dosage information to construct a linkage group and perform QTL interval mapping, the marker phasing analysis in TPMSNP is not entirely automated and requires manual input. Consequently, new beginners’ entry or urgent application while performing a potato breeding program had been discouraged. The two R-packages (MAPpoly and QTLpoly) used in this study overcame the manual marker phasing with these two software having automated most components of genetic and QTL mapping. As a result, the elapsed time for linkage mapping was considerably reduced. Furthermore, the selected 2359 SNPs were evenly distributed across the 12 linkage groups, and genome coverage rates of the genetic maps were high. (Fig. 1; Table 1). The successful outcomes of our research proved the feasibility of fully automated QTL analysis for tetraploid potatoes and expanded the useful information that can be used for the first stage of MAS development for tuber shape and specific gravity in the russet market class.

### Tuber shape QTL on chromosome 10

A major QTL for tuber shape in this mapping population was detected on chromosome 10. Except for the *TS_QTL_ch10_c*, all the other tuber shape QTL on chromosome 10 were observed at 86.75 cM and displayed high LOP scores (Table 3). Even though the *TS_QTL_ch10_c* QTL peak was 7.66 cM away from the others in the genetic map, the support intervals of all nine QTL on chromosome 10 mostly overlapped each other and were narrow, suggesting the presence of single major locus impacting tuber shape on this chromosome (Fig. 3a). The hypothesis was also supported by the narrow gap (0.63 cM) between the physical map location of solcap_snp_c1_11535 linked to *TS_QTL_ch10_c* and those of solcap_snp_c1_15594 linked to the other eight QTL (Table 3). When the two SNPs’ physical map locations were compared with those of 11 reference SNPs previously associated with tuber shape QTL (Table 4), the average distance was only one cM [A. Massa, pers. comm., 8, 11]. The frequent reproducibility of the QTL in multiple studies authenticates the presence of a major gene(s) involving tuber shape formation near solcap_snp_c1_15594 and solcap_snp_c1_11535 in the russet mapping population.

A candidate gene for the major QTL found on chromosome 10 in this study is the *Ro* locus. The single dominant gene (or locus), *Ro*, was first postulated by Masson [7], and it is known to confer round shape which is dominant to long shape. Van Eck et al. [9] mapped the *Ro* locus between TAc13b and Tac20 RFLP markers after analyzing a mapping population obtained from the cross between a female parent having *S. phureja* and Chippewa genetic background and a male parent carrying *S. vernei* and *S. tuberosum*. Chen et al. [4] localized the *Ro* locus on chromosome 10 between two BACs, PA28 and PA13_16, based on a full-sib diploid population. Li et al. [54] also mapped the *Ro* locus and another locus associated with eye depth on the same chromosome between STM0051 (SSR) and CT240 (RFLP) markers while studying a diploid family. Since physical map locations of STM0051, solcap_snp_c1_15594, and solcap_snp_c1_11535 were available in Spud DB [43, 55], we checked the distance between STM0051 (physical map position: ~ 23,484,600) and solcap_snp_c1_15594 or solcap_snp_c1_11535 (Table 4). CT240 could not be compared with them because its physical map location was not available in Spud DB. At least 26 MB or longer gaps were observed between STM0051 and solcap_snp_c1_11535 (or solcap_snp_c1_15594), making it challenging to conclude whether the QTL identified on chromosome 10 of our study are localized in close proximity to the *Ro* locus. On the other hand, evidence showing the proximity of solcap_snp_c1_11535 (or solcap_snp_c1_15594) to *Ro* locus was observed in Endelman and Jansky [11]. For instance, they found nine SNP markers (EJ_SNPs) near the *Ro* locus (Table 4). When the physical map positions of the EJ_SNPs were compared with those of solcap_snp_c1_11535 and solcap_snp_c1_15594 through Spud DB, it was revealed that the EJ_SNPs encompassed the two SNPs (Table 4). Furthermore, a QTL and a candidate SNP (solcap_snp_c2_27831) for tuber shape on chromosome 10 was previously identified in the mapping population (A05141) used in this study (Table 4) [A. Massa, pers. comm.]. With this mapping population representing the russet market class, which is characterized by long tubers, it may be that the QTL identified as impacting tuber shape on chromosome 10 may be closely associated with the *Ro* locus. Fine mapping would be needed with a larger population size and more molecular markers, including known *Ro* locus linked markers, to clarify the proximity of those SNP markers to *Ro* locus. According to Spud DB, all the 12 SNPs mentioned above are close to the Bel1-homeotic protein gene (PGSC0003DMG400019142). Sharma et al. [56] reported that Bel1-like genes, including PGSC0003DMG400019142 on chromosome 10, have a key role in tuber development. Additional studies have also reported other QTL, besides *Ro*, being associated with tuber appearance (e.g., eye depth) on chromosome 10, [8, 9, 11, 14, 54]. Therefore, chromosome 10 appears to be a chromosome having a major impact on tuber shape based on our findings and those of others.

### Tuber shape QTL on chromosome 4 and 7

A QTL at 6.78 cM on chromosome 4 seemed to be uniquely associated with Idaho’s environmental conditions relative to North Carolina with that QTL peak being exclusively observed in the BLUP datasets associated with Idaho (e.g., *TS_clo_ID* and *TS_clo_ID_2010*). Only one *TS_clo_ID* among the nine BLUP datasets showed skewness toward long tuber shape (Supplementary Fig. 5), reinforcing the argument of the more robust relationship between the QTL on chromosome 4 and Idaho environment. Conversely, the QTL at 47.66 cM on chromosome 7 reflects North Carolina’s location and 2011-year effects from the BLUP dataset, *TS_clo_NC_2011* (Table 3). Table 2 also supports this argument with the amount of variance for location and clone × location effects being relatively higher than other effects. This is contrast to the QTL for tuber shape at 86.75 cM on chromosome 10 which was of major consequence across all nine BLUP datasets, indicating this QTL as being less impacted by growing environment relative to the QTL identified on chromosomes 4 and 7.

In our review of publications reporting QTL associated with tuber shape, none reported QTL immediately adjacent to *TS_QTL_ch04_a, _b, _c*, and *TS_QTL_ch07_a.* In 2018, Hara-Skrzypiec et al. [8] reported one DArT marker on chromosome 4. However, the pPt-651,535 DArT marker was 7.67 cM away from solcap_snp_c2_54790, which was linked to *TS_QTL_ch04_a, _b,* and *_c* (Tables 3 & 4). Further research is warranted to determine whether pPt-651,535 and solcap_snp_c2_54790 represent the same locus or not. Meanwhile, these results reveal that two or more genes associated with tuber shape appear to be present on chromosome 4.

From the results and discussion, it becomes apparent that with the exception of the tuber shape QTL on chromosome 10, the other tuber shape QTL detected on chromosomes 4 and 7 in this mapping population were exclusively expressed in either Idaho or North Carolina environments, demonstrating the significance of the genotype × geological environment effect on those QTL. For example, any local biotic or abiotic stresses might cause early maturity or unexpected senescence, impacting the plant’s ability to achieve full size and length under a specific environment relative to another. Therefore, additional analysis for QTL of significance in certain environments may be warranted to facilitate their use in MAS in differing production regions. Meanwhile, a zero variance estimate for the two environmental interaction effects (location x year) was reported in Table 2, indicating that the location effect was independent of the year effect. In other words, the effect of location was the same regardless of years and vice versa.

### Allele effects of tuber shape QTL & single-marker analyses

Through the visualized allele effects (Supplementary Fig. 2a) and single-marker analyses (Supplementary Fig. 6a), detailed features of each allele effect were scrutinized, such as the homologs where each allele is located and their impact.

The phased genotypes of the Rio Grande Russet and Premier Russet at SNP solcap_snp_c1_11535 were BBBB and BABB, respectively (Supplementary Fig. 3). When the allele effects of the QTL at 79.09 and 86.75 cM on chromosome 10 were compared, the most significant negative allele had been consistently observed on homolog f of Premier Russet (Supplementary Fig. 2a; Supplementary Table 4). Based on the results, it was confirmed that the allele A located on the homolog f is linked to a significant negative (or rounding) effect on tuber shape. Single-marker analysis for solcap_snp_c1_11535 also supports this argument. The progeny segregated into to two genotype groups at solcap_snp_c1_11535, depending on the presence or absence of the A allele (Supplementary Fig. 6a). The genotypic group having the A allele had a significantly lower mean (− 0.18) than the mean (0.23) of the other group. However, this finding was not observed in solcap_snp_c1_15594, which is a marker linked to the QTL at 86.75 cM where genotypic groups were not significantly different from each other (data not shown), even though separated by 7.7 cM from solcap_snp_c1_11535.

The phased genotypes of the Rio Grande Russet and Premier Russet at SNP solcap_snp_c2_54790 were BABA and BBBA, respectively (Supplementary Fig. 3). Interestingly, while analyzing the QTL (*TS_QTL_ch04_a* to *_c*) at 6.78 cM on chromosome 4, positive allele effects were continuously detected on homolog b, d, and h where the allele A is located (Supplementary Fig. 2a). On the other hand, negative allele effects were constantly observed on homolog a, e, and f, where B allele is detected (Supplementary Fig. 2a). This reflects the A allele of solcap_snp_c2_54790 importantly contributes to lengthened tuber shape (at least under the Idaho environment). The single-marker analysis for solcap_snp_c2_54790 showed that the mean of each genotype group decreased in an additive fashion as the number of B alleles in each genotype group increased (Supplementary Fig. 6a), reinforcing the observed relationship between the A allele and longer tuber shape. Especially, the presence and absence of the A allele resulted in a significant difference in genotypic means (Supplementary Fig. 6a). Based on the results above, investigating the information on solcap_snp_c1_11535 and solcap_snp_c2_54790 seems to be a promising starting point for developing a successful MAS for a preferred tuber shape. The phased genotypes of the Rio Grande Russet and Premier Russet at solcap_snp_c2_26012 located on chromosome 7 at 47.66 cM were ABAB and BABB, respectively (Supplementary Fig. 3). Positive allele effects of the QTL were observed on homolog b, d, g and h. Negative allele effects of the same QTL were detected on homolog a, c, e, and f (Supplementary Fig. 2a). In general, positive and negative effects were matched B and A allele, respectively, except for homolog e, which had B allele but showed a negative effect (Supplementary Fig. 3; Supplementary Fig. 2a). In Supplementary Fig. 6a, unlike the previous two examples, the mean did not continuously increase as the number of one type allele (A or B) increases. However, the significant mean difference was observed between the ABBB genotype group and two other genotype groups (AABB and AAAB); thus, the allele B’s additive dosage effect for longer tuber shape is assumed. Further research projects with fine-mapping are necessary to determine an appropriate genetic model of the QTL at 47.66 cM on chromosome 7.

### Specific gravity QTL on chromosomes 5 and 1

Significant QTL for specific gravity were detected on chromosome 5 while examining *SG clo*, *SG_clo_ID, SG_clo_MN, SG_clo_ID_2010, SG_clo_ID_2011*, and *SG_clo_MN_2011*. Except for the *SG_QTL_ch05_c*, all the other specific gravity QTL on chromosome 5 were observed at 51.63 cM with 0.15 to 0.21 *h*^*2*^_*QTL*_. Even though *SG_QTL_ch05_c* was 8.08 cM away from the others, the range between 43.55 and 54.04 cM of support intervals of all the six QTL had overlapped each other, suggesting the presence of single gene instead of multiple genes (Fig. 3b). Interestingly, this QTL was not observed from any BLUP data associated with North Carolina environmental effects, indicating the conditional influence of the QTL. In the previous studies, QTL for specific gravity on chromosome 5 were reported, supporting the importance of studying the chromosome 5 to achieve efficient MAS [20–22].

On chromosome 1, three different positions (121.23, 122.30, and 141.26 cM) harbored significant QTL depending on BLUP datasets. Interestingly, two BLUP datasets associated with North Carolina (*SG_clo_NC* and *SG_clo_NC_2011*) produced QTL peak on 141.26 cM, but the other three BLUP datasets (*SG_clo, SG_clo_ID_2011*, and *SG_clo_MN_2011*) resulted in their QTL perks near 122 cM. Furthermore, no QTL was detected from any BLUP data related to the 2010-year effect. Based on those results, there would be two (or more) QTL on chromosome 1, and the QTL would be significantly affected by year and location effects. Table 2 showed much higher variances in location and year × location effects compared to other variances, supporting the significant G × E impact on the QTL. Freyre and Douches [20] and Li et al. [23] commonly found specific gravity QTL on chromosome 1. Schönhals et al. [24] reported the AGPaseS_snp1612 marker, which is linked to tuber starch content, and was localized on chromosome 1. However, the accordance of the QTL locations between the references and this study could not be confirmed due to the lack of physical map location information of the reference markers.

### Allele effects of specific gravity QTL & single-marker analyses

As described in tuber-shape QTL analyses above, Supplementary Figs. 2b and 6b were used to identify allelic effects of the QTL for specific gravity. When the allele effects of the QTL at 43.55 and 51.63 cM on chromosome 5 were compared, the homologs b, c, and g had negative alleles, with the remainder of the homologs having positive alleles. The phased genotypes of the Rio Grande Russet and Premier Russet at solcap_snp_c2_3452 were BAAB and BAAA, respectively. Unlike the SNP examples discussed in the tuber shape, a specific linkage phase (e.g., coupling or repulsion) between a SNP allele (A or B) and positive (or negative) was not detected. Besides, vague differences among the genotype groups were persistently observed from the single-marker analyses for the solcap_snp_c2_3452 across the five BLUP datasets even though significant mean difference existed between AABB and AAAA genotypic groups (Supplementary Fig. 6b). The single-marker analysis of solcap_snp_c2_42406 on chromosome 5 and solcap_snp_c2_49905 on chromosome 1 also showed a similar pattern (Supplementary Fig. 6b). Therefore, an appropriate genetic model was not found, and the use of the three SNPs for MAS does not appear promising. Chromosome walking or fine-mapping near the solcap_snp_c2_3452, solcap_snp_c2_42406, and solcap_snp_c2_49905 SNP markers is necessary to find an appropriate polymorphism associated with specific gravity useful for MAS. Bulked-segregant analysis for the three SNPs with greater population numbers and varied potato clones would also be helpful.

Meanwhile, the solcap_snp_c1_1847 and solcap_snp_c2_7053, which were linked to the QTL on chromosome 1, could not be investigated because the means of different genotype groups were not significantly different from each other (data not included).

## Conclusion

Long or oblong russet potato varieties with an appropriate specific gravity are required by the potato industry for the production of French fries. This study provided important genetic information associated with longer tuber shape across growing environments, which significantly affects the russet-skinned market class. Similar to previously published findings, a major QTL on chromosome 10 was identified in this russet mapping population associated with tuber shape across growing environments with environment-specific QTL also being identified on chromosomes 4 and 7 that were of consequence in Idaho and North Carolina environments, respectively. Significant QTL for specific gravity were oftentimes specific to certain growing environments. For example, a QTL for chromosome 5 was identified with significance in Idaho and Minnesota, but not in North Carolina. Two additional significant QTL were discovered in close proximity on chromosome 1, but with one being significant in North Carolina whereas the other was of more significance in Idaho and Minnesota. The results of this study have identified QTL that can be further explored for the development of markers useful for marker-assisted selection in the russet market.

## Supplementary Information

**Additional file 1: Supplementary Figure S1**. Scale for tuber shape measurement (SolCAP 2009)

**Additional file 2: Supplementary Figure S2**. Allele effects of the SNPs at the mapped QTL. a. Tuber Shape. b. Specific Gravity. The "a" to "d" at the X-axis represents four phased homologs of Rio Grande Russet, and the "e" to "h" represent another four homologs of Premier Russet. Y-axis displayed the quantity of an allele effect on each homolog (Unit does not exist). “solcap_snp_” was omitted at the beginning of all the SNP marker names.

**Additional file 3: Supplementary Figure S3**. Twelve tetraploid linkage groups of two parents. ** Supplementary Fig. 3 is uploaded as a separate PDF file**. (PDF 18802 kb)**

**Additional file 4: Supplementary Figure S4** Tuber shape segregation pattern with TS scores

**Additional file 5: Supplementary Figure S5** Distribution of the BLUP datasets of tuber shape and specific gravity. BLUP data abbreviations: tuber shape (*TS*), specific gravity (*SG*), a genetic effect of clones (*clo*), Idaho (*ID*), North Carolina (*NC*), Minnesota (*MN*) location effects, 2010 (*2010*), and 2011 (*2011*) year effects.

**Additional file 6: Supplementary Figure S6** Single-Marker analyses. a. Tuber Shape. b. Specific Gravity. Tukey-Kramer mean comparison test was used for the single-marker analysis (*p*-value < 0.05). If the *p*-value is below 0.05, the two groups were significantly different from each other and are indicated as so under the Group heading by assignment of letters “A” and “B”; e.g., if the two genotypic groups are significantly different, they will be designated A and B, respectively. If a genotype group is not significantly different from another group, it will receive an “AB” designation. The differences are also visualized through circles. For instance, if two circles above do not overlap, the two means are considered significantly different from each other. “solcap_snp_” was omitted at the beginning of all the SNP marker names.

**Additional file 7: Supplementary Table S1** Genotype data of the A05141 mapping population. (CSV 2393 KB). ** Supplementary Table 1 is uploaded as a separate CSV file. (CSV 2337 kb)

**Additional file 8: Supplementary Table S2** Tuber shape and specific gravity phenotype data of the A05141 mapping population (XLSX 101 KB). ** Supplementary Table 2 is uploaded as a separate Excel file.

**Additional file 9: Supplementary Table S3** Summary of the BLUP datasets (DOCX 16.1 KB). ** Supplementary Table 3 is uploaded as a separate Microsoft Word file.

**Additional file 10: Supplementary Table S4** Detailed information on the allele effects of tuber shape and specific gravity (XLSX 14.7 KB). ** Supplementary Table 4 is uploaded as a separate Excel file.

## Data Availability

All data analyzed during this study are included in this published article and its supplementary information files.

## Abbreviations

BLUP: Best Linear Unbiased Predictors
DArT: Diversity Arrays Technology
MDS: Multidimensional Scaling
QTL: Quantitative Trait Locus
SNP: Single Nucleotide Polymorphism
SG: Specific Gravity
TS: Tuber Shape
UPGMA: Unweighted Pair Group Method with Arithmetic Mean

## References

[1] Suttle J. Symposium Introduction: Enhancing the Nutritional Value of Potato Tubers. American Journal of Potato Research. 2008;85:266.

[2] USDA, National Agricultural Statistics Service: Potatoes 2019 Summary. https://usda.library.cornell.edu/concern/publications/fx719m44h (2020). Accessed 02 Dec 2020.

[3] Stark JC, Love SL, Knowles NR, Stark J, Thornton M, Nolte P (2020). Tuber quality. Potato production systems.

[4] Chen N, Zhu W, Xu J, Duan S, Bian C, Hu J (2019). Molecular marker development and primary physical map construction for the tuber shape Ro gene locus in diploid potato (Solanum tuberosum L.). Mol Breed.

[5] Vreugdenhil D, Bradshaw J, Gebhardt C, Govers F, Taylor MA, MacKerron DK (2011). Potato biology and biotechnology: advances and perspectives: advances and perspectives.

[6] De Jong H (1993). Burns VJ inheritance of tuber shape in cultivated diploid potatoes. American Potato Journal..

[7] Masson MF. Mapping, combining abilities, heritabilities and heterosis with 4x X 2x crosses in potato. Ph.D. Thesis, Univ. of Wisconsin; 1985. 119 p. Diss Abstr Int 46B(5): 1448B.

[8] Hara-Skrzypiec A, Śliwka J, Jakuczun H, Zimnoch-Guzowska E (2018). QTL for tuber morphology traits in diploid potato. J Appl Genet.

[9] Van Eck HJ, Jacobs JME, Stam P, Ton J, Stiekema WJ, Jacobsen E (1994). Multiple alleles for tuber shape in diploid potato detected by qualitative and quantitative genetic analysis using RFLPs. Genetics..

[10] Zhu VW. Genetic mapping and molecular markers development of tuber shape gene in potato. Master thesis, Chinese Academy of Agricultural Sciences; 2015.

[11] Endelman JB, Jansky SH (2016). Genetic mapping with an inbred line-derived F2 population in potato. Theor Appl Genet.

[12] Manrique-Carpintero NC, Coombs JJ, Pham GM, Laimbeer FPE, Braz GT, Jiang J (2018). Genome reduction in tetraploid potato reveals genetic load, haplotype variation, and loci associated with agronomic traits. Front Plant Sci.

[13] Meijer D, Viquez-Zamora M, van Eck HJ, Hutten RCB, Su Y, Rothengatter R, et al. QTL mapping in diploid potato by using selfed progenies of the cross *S. tuberosum* 3 *S. chacoense*. Euphytica. 2018;214:121. 10.1007/s10681-018-2191-6 PMC6434985 30996395

[14] Prashar A, Hornyik C, Young V, McLean K, Sharma SK, Dale MFB (2014). Construction of a dense SNP map of a highly heterozygous diploid potato population and QTL analysis of tuber shape and eye depth. Theor Appl Genet.

[15] Bradshaw JE, Hackett CA, Pande B, Waugh R, Bryan GJ (2008). QTL mapping of yield, agronomic and quality traits in tetraploid potato (Solanum tuberosum subsp. tuberosum). Theor Appl Genet.

[16] Sliwka J, Wasilewicz-Flis I, Jakuczun H, Gebhardt C (2008). Tagging quantitative trait loci for dormancy, tuber shape, regularity of tuber shape, eye depth and flesh colour in diploid potato originated from six solanum species. Plant Breed.

[17] Stevenson FJ, Akeley RV, McLean JG (1954). Potato utilization in relation to variety (heredity) and environment. American Potato Journal..

[18] Johansen RH, Miller JC, Newsom DW, Fontenot JF (1967). The influence of environment on the specific gravity, plant maturity and vigor of potato progenies. American Potato Journal..

[19] Ruttencutter G, Haynes F, Moll R (1979). Estimation of narrow-sense heritability for specific gravity in diploid potatoes (S. tuberosum subsp. Phureja and stenotomum). American Potato Journal.

[20] Freyre R, Douches DS (1994). Development of a model for marker-assisted selection of specific gravity in diploid potato across environments. Crop Sci.

[21] Schäfer-Pregl R, Ritter E, Concilio L, Hesselbach J, Lovatti L, Walkemeier B (1998). Analysis of quantitative trait loci (QTLs) and quantitative trait alleles (QTAs) for potato tuber yield and starch content. Theor Appl Genet.

[22] Li L, Paulo MJ, Strahwald J, Lübeck J, Hofferbert HR, Tacke E (2008). Natural DNA variation at candidate loci is associated with potato chip color, tuber starch content, yield and starch yield. Theor Appl Genet.

[23] Li J, Wang Y, Wen G, Li G, Li Z, Zhang R (2019). Mapping QTL underlying tuber starch content and plant maturity in tetraploid potato. The Crop Journal.

[24] Schönhals EM, Ortega F, Barandalla L, Aragones A, Ruiz de Galarreta JI, Liao JC (2016). Identification and reproducibility of diagnostic DNA markers for tuber starch and yield optimization in a novel association mapping population of potato (*Solanum tuberosum* L). Theoretical and Applied Genetics.

[25] Douches D, Hirsch CN, Manrique-Carpintero NC, Massa AN, Coombs J, Hardigan M (2014). The contribution of the Solanaceae coordinated agricultural project to potato breeding. Potato Res.

[26] Massa AN, Manrique-Carpintero NC, Coombs J, Haynes KG, Bethke PC, Brandt TL (2018). Linkage analysis and QTL mapping in a tetraploid russet mapping population of potato. BMC Genet.

[27] Holm D, Davidson R, Essah S (2004). Rio Grande russet: a new high-quality fresh market russet. Colorado Spud Items.

[28] Novy RG, Whitworth JL, Stark JC, Love SL, Corsini DL, Pavek JJ (2008). Premier russet: a dual-purpose, potato cultivar with significant resistance to low temperature sweetening during long-term storage. Am J Potato Res.

[29] Solanaceae Coordinated Agricultural Project (SolCAP): Potato phenotypic data. http://solcap.msu.edu/potato_phenotype_data.shtml (2009). Accessed 01 Feb 2021.

[30] Barr DJ, Levy R, Scheepers C, Tily HJ (2013). Random effects structure for confirmatory hypothesis testing: keep it maximal. J Mem Lang.

[31] Fernando RL, Grossman M (1989). Marker assisted selection using best linear unbiased prediction. Genet Sel Evol.

[32] Peixouto LS, Nunes JAR, Furtado DF (2016). Factor analysis applied to the G+GE matrix via REML/BLUP for multi-environment data. Crop Breeding and Applied Biotechnology.

[33] Rak K, Bethke PC, Palta JP (2017). QTL mapping of potato chip color and tuber traits within an autotetraploid family. Mol Breed.

[34] Schmidt P, Hartung J, Bennewitz J, Piepho HP (2019). Heritability in plant breeding on a genotype-difference basis. Genetics..

[35] Park J, Hackett CA, Dandurand LM, Wang X, De Jong WS (2019). QTL for resistance to Globodera rostochiensis Pathotype Ro2 and G. pallida Pathotype Pa2/3 in Autotetraploid potato. Am J Potato Res.

[36] Staaf J, Vallon-Christersson J, Lindgren D, Juliusson G, Rosenquist R, Höglund M (2008). Normalization of Illumina Infinium whole-genome SNP data improves copy number estimates and allelic intensity ratios. BMC Bioinformatics.

[37] Schmitz Carley CA, Coombs JJ, Douches DS, Bethke PC, Palta JP, Novy RG (2017). Automated tetraploid genotype calling by hierarchical clustering. Theor Appl Genet.

[38] Mollinari M, Garcia AAF (2019). Linkage Analysis and Haplotype Phasing in Experimental Autopolyploid Populations with High Ploidy Level Using Hidden Markov Models. G3: Genes|Genomes|Genetics.

[39] Core R. Team. R: a language and environment for statistical computing. Vienna: R Foundation for statistical. Computing. 2020; www.R-project.org.

[40] Mollinari M, Olukolu BA, da S. Pereira G, Khan A, Gemenet D, Yencho GC, et al. Unraveling the Hexaploid Sweetpotato Inheritance Using Ultra-Dense Multilocus Mapping. G3: Genes|Genomes|Genetics. 2020;10:281–292. 10.1534/g3.119.400620 PMC6945028 31732504

[41] Hackett CA, Luo ZW (2003). Computer note, TetraploidMap: construction of a linkage map in Autotetraploid species. J Hered.

[42] Preedy KF, Hackett CA (2016). A rapid marker ordering approach for high-density genetic linkage maps in experimental autotetraploid populations using multidimensional scaling. Theor Appl Genet.

[43] Spud Database: Potato Genomics Resource. http://solanaceae.plantbiology.msu.edu. Accessed 02 Dec 2020.

[44] Sharma SK, Bolser D, de Boer J, Sønderkær M, Amoros W, Carboni MF, et al. Construction of reference chromosome-scale Pseudomolecules for potato: Integrating the potato genome with genetic and physical maps. G3: Genes|Genomes|Genetics. 2013;3:2031–2047. 10.1534/g3.113.007153 PMC3815063 24062527

[45] da Silva PG, Gemenet DC, Mollinari M, Olukolu BA, Wood JC, Diaz F (2020). Multiple QTL mapping in Autopolyploids: a random-effect model approach with application in a Hexaploid Sweetpotato full-sib population. Genetics..

[46] Qu L, Guennel T, Marshall SL (2013). Linear score tests for variance components in linear mixed models and applications to genetic association studies. Biometrics..

[47] Lander ES, Green P. Construction of multilocus genetic linkage maps in humans. Proceedings of the National Academy of Sciences of the United States of America (PNAS). 1987;84:2363–2367. 10.1073/pnas.84.8.2363 PMC304651 3470801

[48] Hackett CA, Boskamp B, Vogogias A, Preedy K, Milne I (2017). TetraploidSNPMap: software for linkage analysis and QTL mapping in autotetraploid populations using SNP dosage data. J Hered.

[49] Hamilton JP, Hansey CN, Whitty BR, Stoffel K, Massa AN, Deynze AV (2011). Single nucleotide polymorphism discovery in elite north American potato germplasm. BMC Genomics.

[50] Massa AN, Manrique-Carpintero NC, Coombs JJ, Zarka DG, Boone AE, Kirk WW, et al. Genetic linkage mapping of economically important traits in cultivated tetraploid potato (*Solanum tuberosum* L.). G3: Genes|genomes|genetics. 2015;5:2357–2364. 10.1534/g3.115.019646 PMC4632055 26374597

[51] Mengist MF, Alves S, Griffin D, Creedon J, McLaughlin MJ, Jones PW (2018). Genetic mapping of quantitative trait loci for tuber-cadmium and zinc concentration in potato reveals associations with maturity and both overlapping and independent components of genetic control. Theor Appl Genet.

[52] Santa JD, Berdugo-Cely J, Cely-Pardo L, Soto-Suarez M, Mosquera T, Galeano M. CH. QTL analysis reveals quantitative resistant loci for *Phytophthora infestans* and Tecia solanivora in tetraploid potato (*Solanum tuberosum* L.). PLoS ONE. 2018;13(7):e0199716. 10.1371/journal.pone.0199716 PMC6034811 29979690

[53] da Silva WL, Ingram J, Hackett CA, Coombs JJ, Douches D, Bryan GJ, et al. Mapping loci that control tuber and foliar symptoms caused by PVY in autotetraploid potato (*Solanum tuberosum* L.). G3: Genes|Genomes|Genetics. 2017;7:3587–3595. 10.1534/g3.117.300264 PMC5675608 28903982

[54] Li XQ, De Jong H, De Jong DM, De Jong WS (2005). Inheritance and genetic mapping of tuber eye depth in cultivated diploid potatoes. Theor Appl Genet.

[55] Hirsch CD, Hamilton JP, Childs KL, Cepela J, Crisovan E, Vaillancourt B (2014). Spud DB: a resource for mining sequences, genotypes, and phenotypes to accelerate potato breeding. The Plant Genome.

[56] Sharma P, Lin T, Grandellis C, Yu M, Hannapel DJ (2014). The BEL1-like family of transcription factors in potato. J Exp Bot.

