# Supplementary material for: Linkage and QTL mapping for tuber shape and specific gravity in a tetraploid mapping population of potato representing the russet market class
Source: BMC Plant Biol. 2021 Nov 3;21:507. doi: 10.1186/s12870-021-03265-2 (PMC8565078; doi:10.1186/s12870-021-03265-2)
Supplement: Supplementary file 3 — Additional file 3: Supplementary Figure S3. Twelve tetraploid linkage groups of two parents. ** Supplementary Fig. 3 is uploaded as a separate PDF file. (PDF 18802 kb) [file 12870_2021_3265_MOESM3_ESM.pdf]

chr01_Rio_Grande_Russet

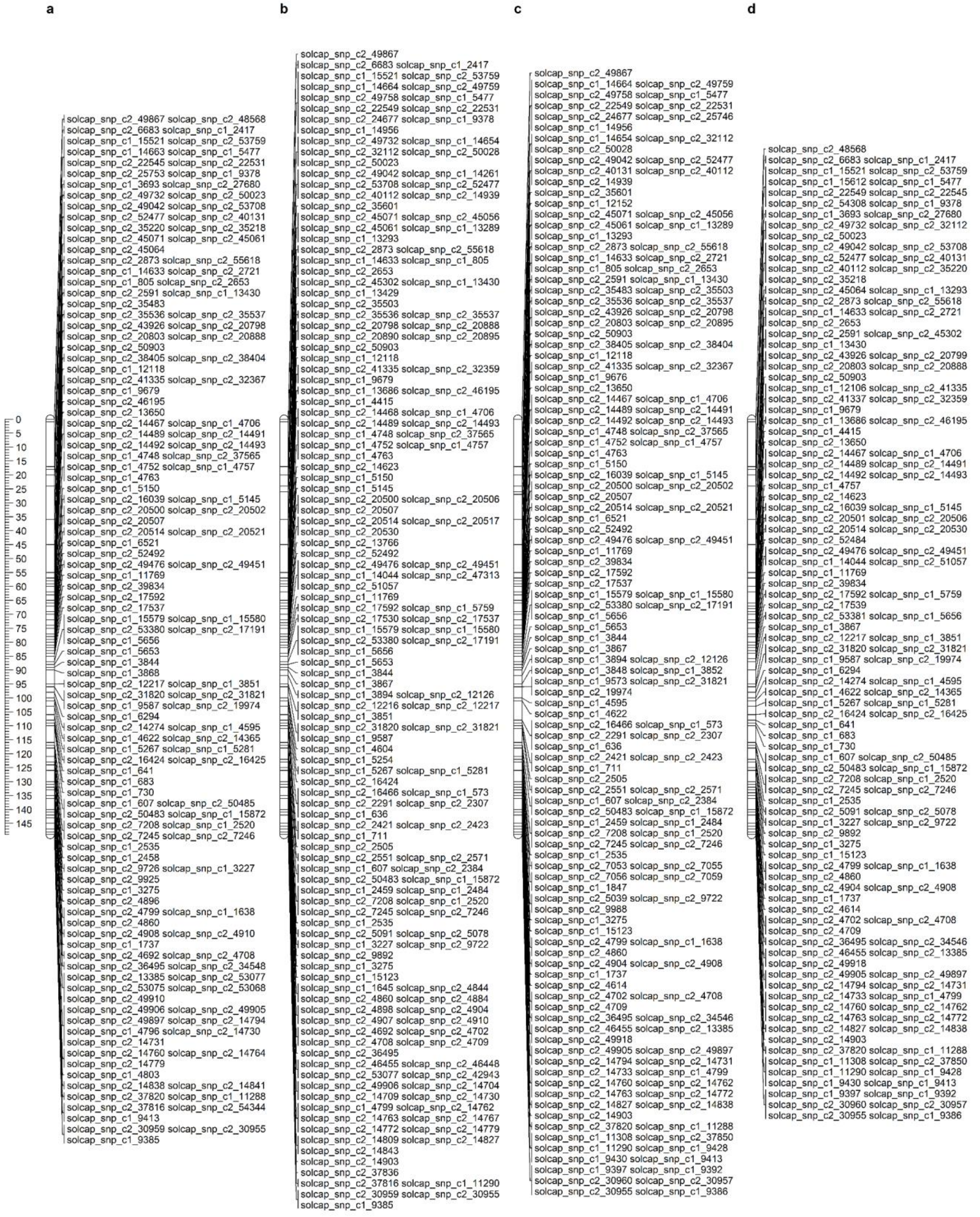

chr01_Premier_Russet

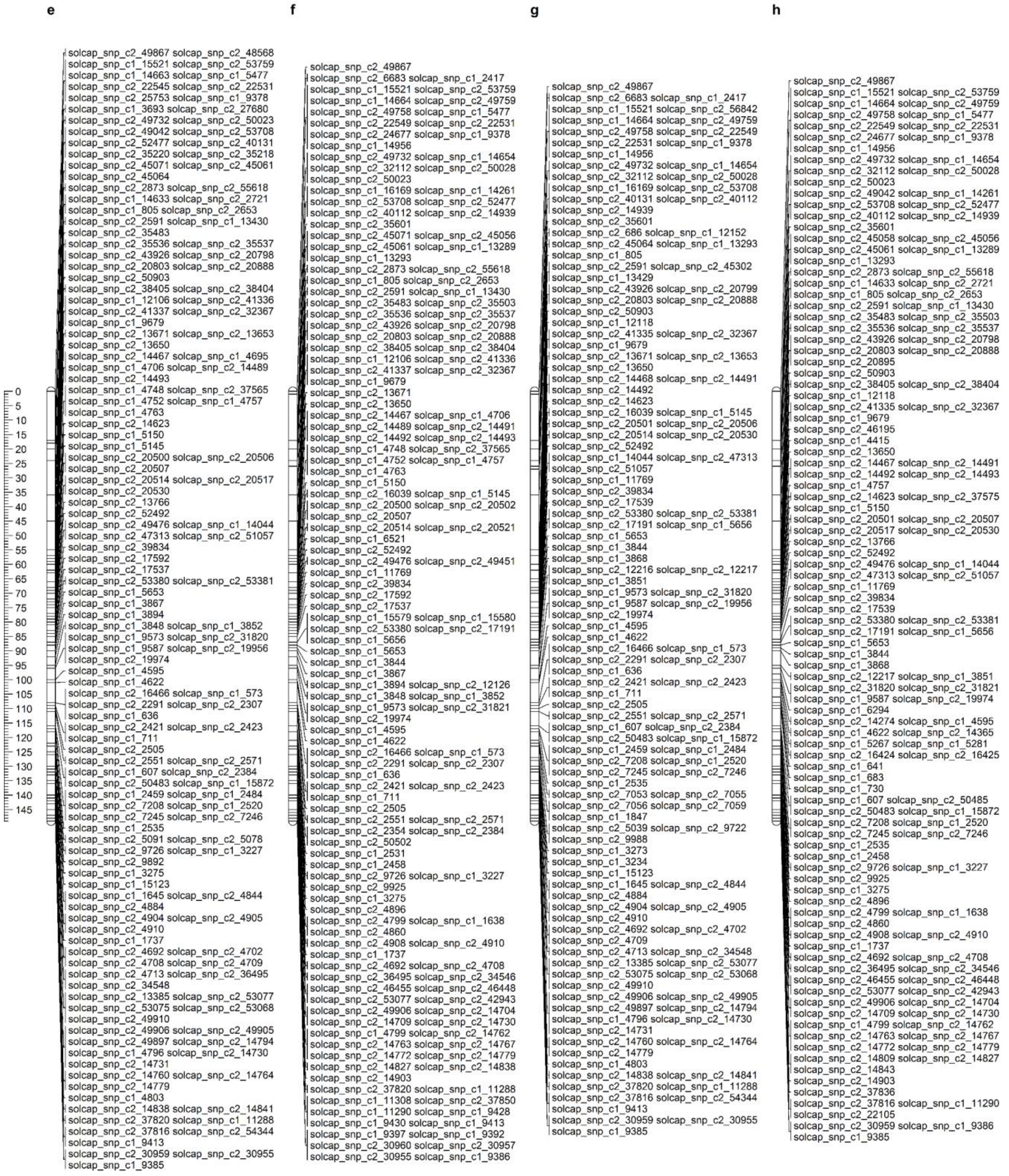

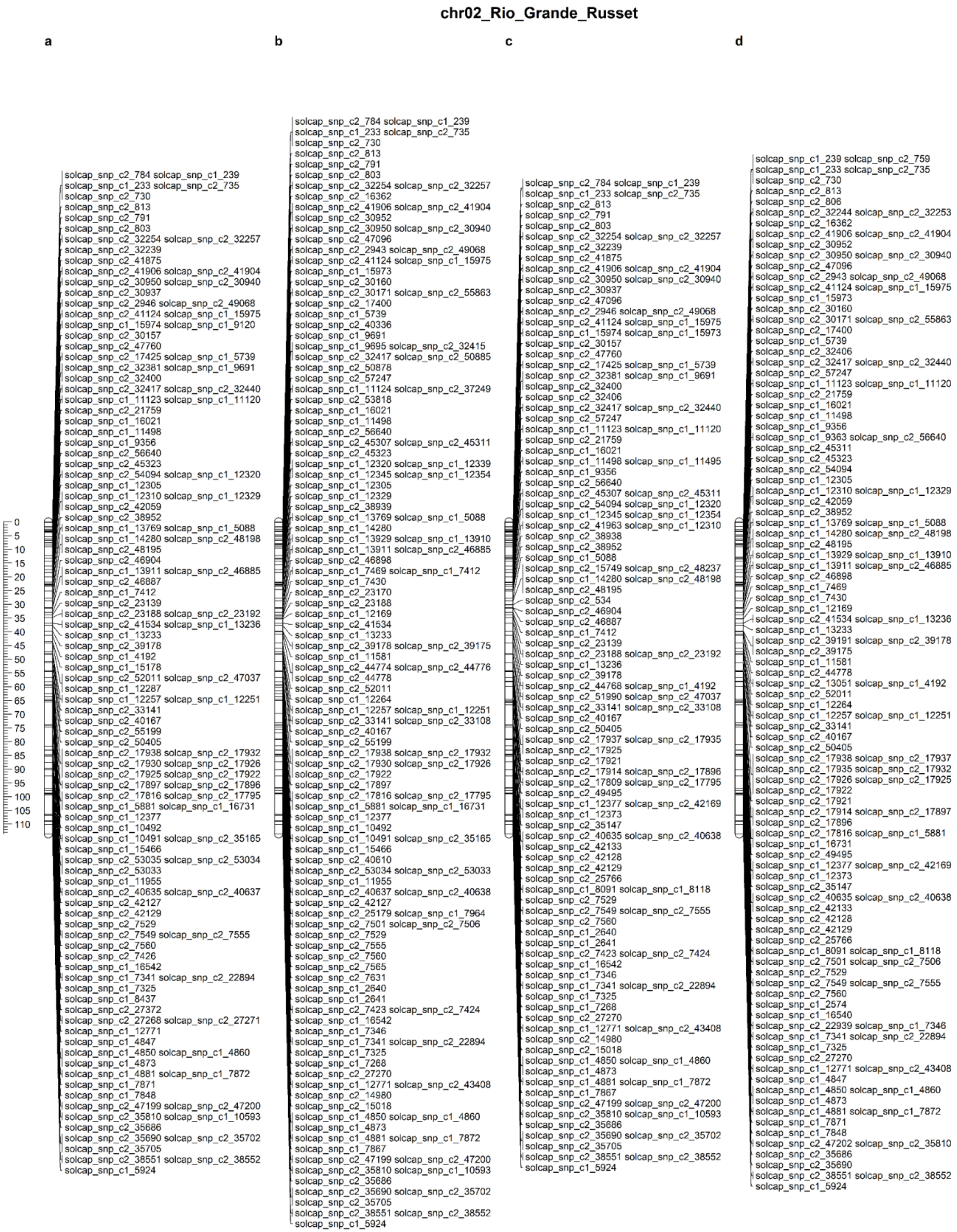
chr02_Rio_Grande_Russet
a
b
c
d
0
5
10
15
20
25
30
35
40
45
50
55
60
65
70
75
80
85
90
95
100
105
110
solcap_snp_c2_784 solcap_snp_c1_239
solcap_snp_c1_233 solcap_snp_c2_735
solcap_snp_c2_730
solcap_snp_c2_813
solcap_snp_c2_791
solcap_snp_c2_803
solcap_snp_c2_32254 solcap_snp_c2_32257
solcap_snp_c2_32239
solcap_snp_c2_41875
solcap_snp_c2_41906 solcap_snp_c2_41904
solcap_snp_c2_30950 solcap_snp_c2_30940
solcap_snp_c2_30937
solcap_snp_c2_2946 solcap_snp_c2_49068
solcap_snp_c2_41124 solcap_snp_c1_15975
solcap_snp_c1_15974 solcap_snp_c1_9120
solcap_snp_c2_30157
solcap_snp_c2_47760
solcap_snp_c2_17425 solcap_snp_c1_5739
solcap_snp_c2_32381 solcap_snp_c1_9691
solcap_snp_c2_32400
solcap_snp_c2_32417 solcap_snp_c2_32440
solcap_snp_c1_11123 solcap_snp_c1_11120
solcap_snp_c2_21759
solcap_snp_c1_16021
solcap_snp_c1_11498
solcap_snp_c1_9356
solcap_snp_c2_56640
solcap_snp_c2_45323
solcap_snp_c2_54094 solcap_snp_c1_12320
solcap_snp_c1_12305
solcap_snp_c1_12310 solcap_snp_c1_12329
solcap_snp_c2_42059
solcap_snp_c2_38952
solcap_snp_c1_13769 solcap_snp_c1_5088
solcap_snp_c1_14280 solcap_snp_c2_48198
solcap_snp_c2_48195
solcap_snp_c2_46904
solcap_snp_c1_13911 solcap_snp_c2_46885
solcap_snp_c2_46887
solcap_snp_c1_7412
solcap_snp_c2_23139
solcap_snp_c2_23188 solcap_snp_c2_23192
solcap_snp_c2_41534 solcap_snp_c1_13236
solcap_snp_c1_13233
solcap_snp_c2_39178
solcap_snp_c1_4192
solcap_snp_c1_15178
solcap_snp_c2_52011 solcap_snp_c2_47037
solcap_snp_c1_12287
solcap_snp_c1_12257 solcap_snp_c1_12251
solcap_snp_c2_33141
solcap_snp_c2_40167
solcap_snp_c2_55199
solcap_snp_c2_50405
solcap_snp_c2_17938 solcap_snp_c2_17932
solcap_snp_c2_17930 solcap_snp_c2_17926
solcap_snp_c2_17925 solcap_snp_c2_17922
solcap_snp_c2_17897 solcap_snp_c2_17896
solcap_snp_c2_17816 solcap_snp_c2_17795
solcap_snp_c1_5881 solcap_snp_c1_16731
solcap_snp_c1_12377
solcap_snp_c1_10492
solcap_snp_c1_10491 solcap_snp_c2_35165
solcap_snp_c1_15466
solcap_snp_c2_53035 solcap_snp_c2_53034
solcap_snp_c2_53033
solcap_snp_c1_11955
solcap_snp_c2_40635 solcap_snp_c2_40637
solcap_snp_c2_42127
solcap_snp_c2_42129
solcap_snp_c2_7529
solcap_snp_c2_7549 solcap_snp_c2_7555
solcap_snp_c2_7560
solcap_snp_c2_7426
solcap_snp_c1_16542
solcap_snp_c1_7341 solcap_snp_c2_22894
solcap_snp_c1_7325
solcap_snp_c1_8437
solcap_snp_c2_27372
solcap_snp_c2_27268 solcap_snp_c2_27271
solcap_snp_c1_12771
solcap_snp_c1_4847
solcap_snp_c1_4850 solcap_snp_c1_4860
solcap_snp_c1_4873
solcap_snp_c1_4881 solcap_snp_c1_7872
solcap_snp_c1_7871
solcap_snp_c1_7848
solcap_snp_c2_47199 solcap_snp_c2_47200
solcap_snp_c2_35810 solcap_snp_c1_10593
solcap_snp_c2_35686
solcap_snp_c2_35690 solcap_snp_c2_35702
solcap_snp_c2_35705
solcap_snp_c2_38551 solcap_snp_c2_38552
solcap_snp_c1_5924
solcap_snp_c2_784 solcap_snp_c1_239
solcap_snp_c1_233 solcap_snp_c2_735
solcap_snp_c2_730
solcap_snp_c2_813
solcap_snp_c2_791
solcap_snp_c2_803
solcap_snp_c2_32254 solcap_snp_c2_32257
solcap_snp_c2_16362
solcap_snp_c2_41906 solcap_snp_c2_41904
solcap_snp_c2_30952
solcap_snp_c2_30950 solcap_snp_c2_30940
solcap_snp_c2_47096
solcap_snp_c2_2943 solcap_snp_c2_49068
solcap_snp_c2_41124 solcap_snp_c1_15975
solcap_snp_c1_15973
solcap_snp_c2_30160
solcap_snp_c2_30171 solcap_snp_c2_55863
solcap_snp_c2_17400
solcap_snp_c1_5739
solcap_snp_c2_40336
solcap_snp_c1_9691
solcap_snp_c1_9695 solcap_snp_c2_32415
solcap_snp_c2_32417 solcap_snp_c2_50885
solcap_snp_c2_50878
solcap_snp_c2_57247
solcap_snp_c1_11124 solcap_snp_c2_37249
solcap_snp_c2_53818
solcap_snp_c1_16021
solcap_snp_c1_11498
solcap_snp_c2_56640
solcap_snp_c2_45307 solcap_snp_c2_45311
solcap_snp_c2_45323
solcap_snp_c1_12320 solcap_snp_c1_12339
solcap_snp_c1_12345 solcap_snp_c1_12354
solcap_snp_c1_12305
solcap_snp_c1_12329
solcap_snp_c2_38939
solcap_snp_c1_13769 solcap_snp_c1_5088
solcap_snp_c1_14280
solcap_snp_c1_13929 solcap_snp_c1_13910
solcap_snp_c1_13911 solcap_snp_c2_46885
solcap_snp_c2_46898
solcap_snp_c1_7469 solcap_snp_c1_7412
solcap_snp_c1_7430
solcap_snp_c2_23170
solcap_snp_c2_23188
solcap_snp_c1_12169
solcap_snp_c2_41534
solcap_snp_c1_13233
solcap_snp_c2_39178 solcap_snp_c2_39175
solcap_snp_c1_11581
solcap_snp_c2_44774 solcap_snp_c2_44776
solcap_snp_c2_44778
solcap_snp_c2_52011
solcap_snp_c1_12264
solcap_snp_c1_12257 solcap_snp_c1_12251
solcap_snp_c2_33141 solcap_snp_c2_33108
solcap_snp_c2_40167
solcap_snp_c2_55199
solcap_snp_c2_17938 solcap_snp_c2_17932
solcap_snp_c2_17930 solcap_snp_c2_17926
solcap_snp_c2_17922
solcap_snp_c2_17897
solcap_snp_c2_17816 solcap_snp_c2_17795
solcap_snp_c1_5881 solcap_snp_c1_16731
solcap_snp_c1_12377
solcap_snp_c1_10492
solcap_snp_c1_10491 solcap_snp_c2_35165
solcap_snp_c1_15466
solcap_snp_c2_40610
solcap_snp_c2_53034 solcap_snp_c2_53033
solcap_snp_c1_11955
solcap_snp_c2_40637 solcap_snp_c2_40638
solcap_snp_c2_42127
solcap_snp_c2_25179 solcap_snp_c1_7964
solcap_snp_c2_7501 solcap_snp_c2_7506
solcap_snp_c2_7529
solcap_snp_c2_7555
solcap_snp_c2_7560
solcap_snp_c2_7565
solcap_snp_c2_7631
solcap_snp_c1_2640
solcap_snp_c1_2641
solcap_snp_c2_7423 solcap_snp_c2_7424
solcap_snp_c1_16542
solcap_snp_c1_7346
solcap_snp_c1_7341 solcap_snp_c2_22894
solcap_snp_c1_7325
solcap_snp_c1_7268
solcap_snp_c2_27270
solcap_snp_c1_12771 solcap_snp_c2_43408
solcap_snp_c2_14980
solcap_snp_c2_15018
solcap_snp_c1_4850 solcap_snp_c1_4860
solcap_snp_c1_4873
solcap_snp_c1_4881 solcap_snp_c1_7872
solcap_snp_c1_7867
solcap_snp_c2_47199 solcap_snp_c2_47200
solcap_snp_c2_35810 solcap_snp_c1_10593
solcap_snp_c2_35686
solcap_snp_c2_35690 solcap_snp_c2_35702
solcap_snp_c2_35705
solcap_snp_c2_38551 solcap_snp_c2_38552
solcap_snp_c1_5924
solcap_snp_c2_784 solcap_snp_c1_239
solcap_snp_c1_233 solcap_snp_c2_735
solcap_snp_c2_813
solcap_snp_c2_791
solcap_snp_c2_803
solcap_snp_c2_32254 solcap_snp_c2_32257
solcap_snp_c2_32239
solcap_snp_c2_41875
solcap_snp_c2_41906 solcap_snp_c2_41904
solcap_snp_c2_30950 solcap_snp_c2_30940
solcap_snp_c2_30937
solcap_snp_c2_47096
solcap_snp_c2_2946 solcap_snp_c2_49068
solcap_snp_c2_41124 solcap_snp_c1_15975
solcap_snp_c1_15974 solcap_snp_c1_15973
solcap_snp_c2_30157
solcap_snp_c2_47760
solcap_snp_c2_17425 solcap_snp_c1_5739
solcap_snp_c2_32381 solcap_snp_c1_9691
solcap_snp_c2_32400
solcap_snp_c2_32406
solcap_snp_c2_32417 solcap_snp_c2_32440
solcap_snp_c2_57247
solcap_snp_c1_11123 solcap_snp_c1_11120
solcap_snp_c2_21759
solcap_snp_c1_16021
solcap_snp_c1_11498 solcap_snp_c1_11495
solcap_snp_c1_9356
solcap_snp_c2_56640
solcap_snp_c2_45307 solcap_snp_c2_45311
solcap_snp_c2_54094 solcap_snp_c1_12320
solcap_snp_c1_12345 solcap_snp_c1_12354
solcap_snp_c2_41963 solcap_snp_c1_12310
solcap_snp_c2_38938
solcap_snp_c2_38952
solcap_snp_c1_5088
solcap_snp_c2_15749 solcap_snp_c2_48237
solcap_snp_c1_14280 solcap_snp_c2_48198
solcap_snp_c2_48195
solcap_snp_c2_534
solcap_snp_c2_46904
solcap_snp_c2_46887
solcap_snp_c1_7412
solcap_snp_c2_23139
solcap_snp_c2_23188 solcap_snp_c2_23192
solcap_snp_c1_13236
solcap_snp_c2_39178
solcap_snp_c2_44768 solcap_snp_c1_4192
solcap_snp_c2_51990 solcap_snp_c2_47037
solcap_snp_c2_33141 solcap_snp_c2_33108
solcap_snp_c2_40167
solcap_snp_c2_50405
solcap_snp_c2_17937 solcap_snp_c2_17935
solcap_snp_c2_17925
solcap_snp_c2_17921
solcap_snp_c2_17914 solcap_snp_c2_17896
solcap_snp_c2_17809 solcap_snp_c2_17795
solcap_snp_c2_49495
solcap_snp_c1_12377 solcap_snp_c2_42169
solcap_snp_c1_12373
solcap_snp_c2_35147
solcap_snp_c2_40635 solcap_snp_c2_40638
solcap_snp_c2_42133
solcap_snp_c2_42128
solcap_snp_c2_42129
solcap_snp_c2_25766
solcap_snp_c1_8091 solcap_snp_c1_8118
solcap_snp_c2_7529
solcap_snp_c2_7549 solcap_snp_c2_7555
solcap_snp_c2_7560
solcap_snp_c1_2640
solcap_snp_c1_2641
solcap_snp_c2_7423 solcap_snp_c2_7424
solcap_snp_c1_16542
solcap_snp_c1_7346
solcap_snp_c1_7341 solcap_snp_c2_22894
solcap_snp_c1_7325
solcap_snp_c1_7268
solcap_snp_c2_27270
solcap_snp_c1_12771 solcap_snp_c2_43408
solcap_snp_c2_14980
solcap_snp_c2_15018
solcap_snp_c1_4850 solcap_snp_c1_4860
solcap_snp_c1_4873
solcap_snp_c1_4881 solcap_snp_c1_7872
solcap_snp_c1_7867
solcap_snp_c2_47199 solcap_snp_c2_47200
solcap_snp_c2_35810 solcap_snp_c1_10593
solcap_snp_c2_35686
solcap_snp_c2_35690 solcap_snp_c2_35702
solcap_snp_c2_35705
solcap_snp_c2_38551 solcap_snp_c2_38552
solcap_snp_c1_5924
solcap_snp_c1_239 solcap_snp_c2_759
solcap_snp_c1_233 solcap_snp_c2_735
solcap_snp_c2_730
solcap_snp_c2_813
solcap_snp_c2_806
solcap_snp_c2_32244 solcap_snp_c2_32253
solcap_snp_c2_16362
solcap_snp_c2_41906 solcap_snp_c2_41904
solcap_snp_c2_30952
solcap_snp_c2_30950 solcap_snp_c2_30940
solcap_snp_c2_47096
solcap_snp_c2_2943 solcap_snp_c2_49068
solcap_snp_c2_41124 solcap_snp_c1_15975
solcap_snp_c1_15973
solcap_snp_c2_30160
solcap_snp_c2_30171 solcap_snp_c2_55863
solcap_snp_c2_17400
solcap_snp_c1_5739
solcap_snp_c2_32406
solcap_snp_c2_32417 solcap_snp_c2_32440
solcap_snp_c2_57247
solcap_snp_c1_11123 solcap_snp_c1_11120
solcap_snp_c2_21759
solcap_snp_c1_16021
solcap_snp_c1_11498
solcap_snp_c1_9356
solcap_snp_c1_9363 solcap_snp_c2_56640
solcap_snp_c2_45311
solcap_snp_c2_45323
solcap_snp_c2_54094
solcap_snp_c1_12305
solcap_snp_c1_12310 solcap_snp_c1_12329
solcap_snp_c2_42059
solcap_snp_c2_38952
solcap_snp_c1_13769 solcap_snp_c1_5088
solcap_snp_c1_14280 solcap_snp_c2_48198
solcap_snp_c2_48195
solcap_snp_c1_13929 solcap_snp_c1_13910
solcap_snp_c1_13911 solcap_snp_c2_46885
solcap_snp_c2_46898
solcap_snp_c1_7469
solcap_snp_c1_7430
solcap_snp_c1_12169
solcap_snp_c2_41534 solcap_snp_c1_13236
solcap_snp_c1_13233
solcap_snp_c2_39191 solcap_snp_c2_39178
solcap_snp_c2_39175
solcap_snp_c1_11581
solcap_snp_c2_44778
solcap_snp_c2_13051 solcap_snp_c1_4192
solcap_snp_c2_52011
solcap_snp_c1_12264
solcap_snp_c1_12257 solcap_snp_c1_12251
solcap_snp_c2_33141
solcap_snp_c2_40167
solcap_snp_c2_50405
solcap_snp_c2_17938 solcap_snp_c2_17937
solcap_snp_c2_17935 solcap_snp_c2_17932
solcap_snp_c2_17926 solcap_snp_c2_17925
solcap_snp_c2_17922
solcap_snp_c2_17921
solcap_snp_c2_17914 solcap_snp_c2_17897
solcap_snp_c2_17896
solcap_snp_c2_17816 solcap_snp_c1_5881
solcap_snp_c1_16731
solcap_snp_c2_49495
solcap_snp_c1_12377 solcap_snp_c2_42169
solcap_snp_c1_12373
solcap_snp_c2_35147
solcap_snp_c2_40635 solcap_snp_c2_40638
solcap_snp_c2_42133
solcap_snp_c2_42128
solcap_snp_c2_42129
solcap_snp_c2_25766
solcap_snp_c1_8091 solcap_snp_c1_8118
solcap_snp_c2_7501 solcap_snp_c2_7506
solcap_snp_c2_7529
solcap_snp_c2_7549 solcap_snp_c2_7555
solcap_snp_c2_7560
solcap_snp_c1_2574
solcap_snp_c1_16540
solcap_snp_c2_22939 solcap_snp_c1_7346
solcap_snp_c1_7341 solcap_snp_c2_22894
solcap_snp_c1_7325
solcap_snp_c2_27270
solcap_snp_c1_12771 solcap_snp_c2_43408
solcap_snp_c1_4847
solcap_snp_c1_4850 solcap_snp_c1_4860
solcap_snp_c1_4873
solcap_snp_c1_4881 solcap_snp_c1_7872
solcap_snp_c1_7871
solcap_snp_c1_7848
solcap_snp_c2_47202 solcap_snp_c2_35810
solcap_snp_c2_35686
solcap_snp_c2_35690
solcap_snp_c2_38551 solcap_snp_c2_38552
solcap_snp_c1_5924

chr02_Premier_Russet

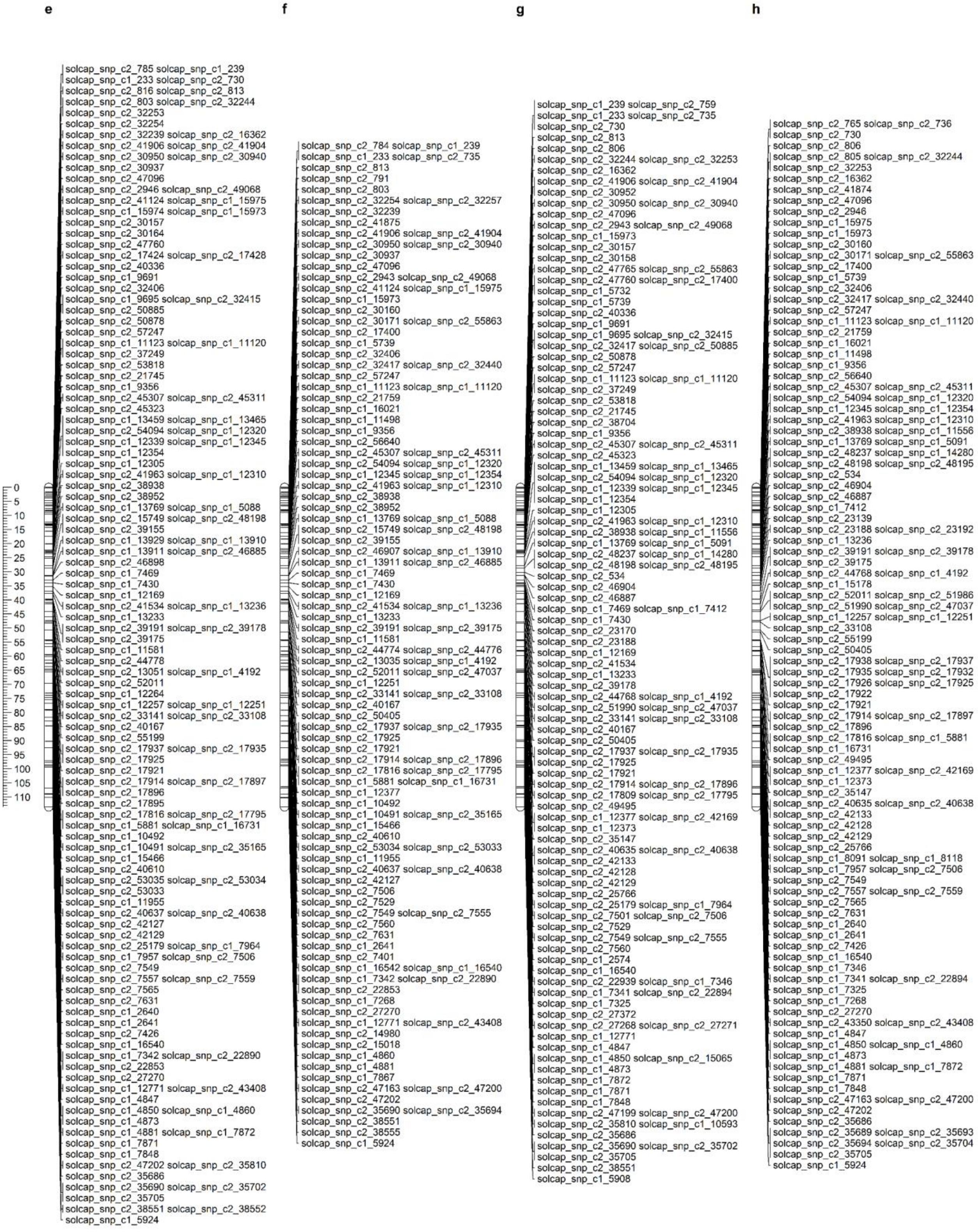

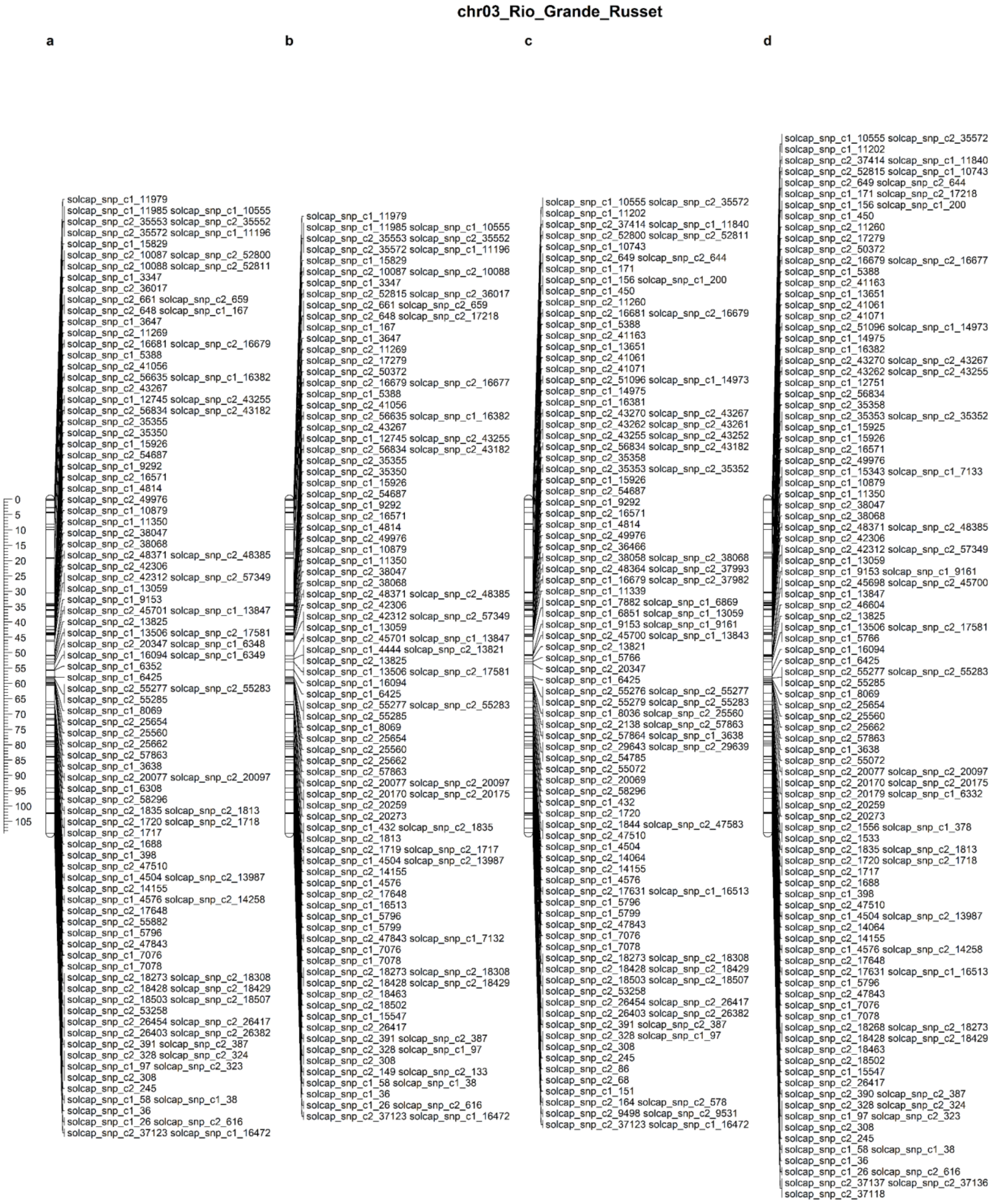
chr03_Rio_Grande_Russet
a
b
c
d
0
5
10
15
20
25
30
35
40
45
50
55
60
65
70
75
80
85
90
95
100
105
solcap_snp_c1_11979
solcap_snp_c1_11985 solcap_snp_c1_10555
solcap_snp_c2_35553 solcap_snp_c2_35552
solcap_snp_c2_35572 solcap_snp_c1_11196
solcap_snp_c1_15829
solcap_snp_c2_10087 solcap_snp_c2_52800
solcap_snp_c2_10088 solcap_snp_c2_52811
solcap_snp_c1_3347
solcap_snp_c2_36017
solcap_snp_c2_661 solcap_snp_c2_659
solcap_snp_c2_648 solcap_snp_c1_167
solcap_snp_c1_3647
solcap_snp_c2_11269
solcap_snp_c2_16681 solcap_snp_c2_16679
solcap_snp_c1_5388
solcap_snp_c2_41056
solcap_snp_c2_56635 solcap_snp_c1_16382
solcap_snp_c2_43267
solcap_snp_c1_12745 solcap_snp_c2_43255
solcap_snp_c2_56834 solcap_snp_c2_43182
solcap_snp_c2_35355
solcap_snp_c2_35350
solcap_snp_c1_15926
solcap_snp_c2_54687
solcap_snp_c1_9292
solcap_snp_c2_16571
solcap_snp_c1_4814
solcap_snp_c2_49976
solcap_snp_c1_10879
solcap_snp_c1_11350
solcap_snp_c2_38047
solcap_snp_c2_38068
solcap_snp_c2_48371 solcap_snp_c2_48385
solcap_snp_c2_42306
solcap_snp_c2_42312 solcap_snp_c2_57349
solcap_snp_c1_13059
solcap_snp_c1_9153
solcap_snp_c2_45701 solcap_snp_c1_13847
solcap_snp_c2_13825
solcap_snp_c1_13506 solcap_snp_c2_17581
solcap_snp_c2_20347 solcap_snp_c1_6348
solcap_snp_c1_16094 solcap_snp_c1_6349
solcap_snp_c1_6352
solcap_snp_c1_6425
solcap_snp_c2_55277 solcap_snp_c2_55283
solcap_snp_c2_55285
solcap_snp_c1_8069
solcap_snp_c2_25654
solcap_snp_c2_25560
solcap_snp_c2_25662
solcap_snp_c2_57863
solcap_snp_c1_3638
solcap_snp_c2_20077 solcap_snp_c2_20097
solcap_snp_c1_6308
solcap_snp_c2_58296
solcap_snp_c2_1835 solcap_snp_c2_1813
solcap_snp_c2_1720 solcap_snp_c2_1718
solcap_snp_c2_1717
solcap_snp_c2_1688
solcap_snp_c1_398
solcap_snp_c2_47510
solcap_snp_c1_4504 solcap_snp_c2_13987
solcap_snp_c2_14155
solcap_snp_c1_4576 solcap_snp_c2_14258
solcap_snp_c2_17648
solcap_snp_c2_55882
solcap_snp_c1_5796
solcap_snp_c2_47843
solcap_snp_c1_7076
solcap_snp_c1_7078
solcap_snp_c2_18273 solcap_snp_c2_18308
solcap_snp_c2_18428 solcap_snp_c2_18429
solcap_snp_c2_18503 solcap_snp_c2_18507
solcap_snp_c2_53258
solcap_snp_c2_26454 solcap_snp_c2_26417
solcap_snp_c2_26403 solcap_snp_c2_26382
solcap_snp_c2_391 solcap_snp_c2_387
solcap_snp_c2_328 solcap_snp_c2_324
solcap_snp_c1_97 solcap_snp_c2_323
solcap_snp_c2_308
solcap_snp_c2_245
solcap_snp_c1_58 solcap_snp_c1_38
solcap_snp_c1_36
solcap_snp_c1_26 solcap_snp_c2_616
solcap_snp_c2_37123 solcap_snp_c1_16472
solcap_snp_c1_11979
solcap_snp_c1_11985 solcap_snp_c1_10555
solcap_snp_c2_35553 solcap_snp_c2_35552
solcap_snp_c2_35572 solcap_snp_c1_11196
solcap_snp_c1_15829
solcap_snp_c2_10087 solcap_snp_c2_10088
solcap_snp_c1_3347
solcap_snp_c2_52815 solcap_snp_c2_36017
solcap_snp_c2_661 solcap_snp_c2_659
solcap_snp_c2_648 solcap_snp_c2_17218
solcap_snp_c1_167
solcap_snp_c1_3647
solcap_snp_c2_11269
solcap_snp_c2_17279
solcap_snp_c2_50372
solcap_snp_c2_16679 solcap_snp_c2_16677
solcap_snp_c1_5388
solcap_snp_c2_41056
solcap_snp_c2_56635 solcap_snp_c1_16382
solcap_snp_c2_43267
solcap_snp_c1_12745 solcap_snp_c2_43255
solcap_snp_c2_56834 solcap_snp_c2_43182
solcap_snp_c2_35355
solcap_snp_c2_35350
solcap_snp_c1_15926
solcap_snp_c2_54687
solcap_snp_c1_9292
solcap_snp_c2_16571
solcap_snp_c1_4814
solcap_snp_c2_49976
solcap_snp_c1_10879
solcap_snp_c1_11350
solcap_snp_c2_38047
solcap_snp_c2_38068
solcap_snp_c2_48371 solcap_snp_c2_48385
solcap_snp_c2_42306
solcap_snp_c2_42312 solcap_snp_c2_57349
solcap_snp_c1_13059
solcap_snp_c2_45701 solcap_snp_c1_13847
solcap_snp_c1_4444 solcap_snp_c2_13821
solcap_snp_c2_13825
solcap_snp_c1_13506 solcap_snp_c2_17581
solcap_snp_c1_16094
solcap_snp_c1_6425
solcap_snp_c2_55277 solcap_snp_c2_55283
solcap_snp_c2_55285
solcap_snp_c1_8069
solcap_snp_c2_25654
solcap_snp_c2_25560
solcap_snp_c2_25662
solcap_snp_c2_57863
solcap_snp_c2_20077 solcap_snp_c2_20097
solcap_snp_c2_20170 solcap_snp_c2_20175
solcap_snp_c2_20259
solcap_snp_c2_20273
solcap_snp_c1_432 solcap_snp_c2_1835
solcap_snp_c2_1813
solcap_snp_c2_1719 solcap_snp_c2_1717
solcap_snp_c1_4504 solcap_snp_c2_13987
solcap_snp_c2_14155
solcap_snp_c1_4576
solcap_snp_c2_17648
solcap_snp_c1_16513
solcap_snp_c1_5796
solcap_snp_c1_5799
solcap_snp_c2_47843 solcap_snp_c1_7132
solcap_snp_c1_7076
solcap_snp_c1_7078
solcap_snp_c2_18273 solcap_snp_c2_18308
solcap_snp_c2_18428 solcap_snp_c2_18429
solcap_snp_c2_18463
solcap_snp_c2_18502
solcap_snp_c1_15547
solcap_snp_c2_26417
solcap_snp_c2_391 solcap_snp_c2_387
solcap_snp_c2_328 solcap_snp_c1_97
solcap_snp_c2_308
solcap_snp_c2_149 solcap_snp_c2_133
solcap_snp_c1_58 solcap_snp_c1_38
solcap_snp_c1_36
solcap_snp_c1_26 solcap_snp_c2_616
solcap_snp_c2_37123 solcap_snp_c1_16472
solcap_snp_c1_10555 solcap_snp_c2_35572
solcap_snp_c1_11202
solcap_snp_c2_37414 solcap_snp_c1_11840
solcap_snp_c2_52800 solcap_snp_c2_52811
solcap_snp_c1_10743
solcap_snp_c2_649 solcap_snp_c2_644
solcap_snp_c1_171
solcap_snp_c1_156 solcap_snp_c1_200
solcap_snp_c1_450
solcap_snp_c2_11260
solcap_snp_c2_16681 solcap_snp_c2_16679
solcap_snp_c1_5388
solcap_snp_c2_41163
solcap_snp_c1_13651
solcap_snp_c2_41061
solcap_snp_c2_41071
solcap_snp_c2_51096 solcap_snp_c1_14973
solcap_snp_c1_14975
solcap_snp_c1_16381
solcap_snp_c2_43270 solcap_snp_c2_43267
solcap_snp_c2_43262 solcap_snp_c2_43261
solcap_snp_c2_43255 solcap_snp_c2_43252
solcap_snp_c2_56834 solcap_snp_c2_43182
solcap_snp_c2_35358
solcap_snp_c2_35353 solcap_snp_c2_35352
solcap_snp_c1_15926
solcap_snp_c2_54687
solcap_snp_c1_9292
solcap_snp_c2_16571
solcap_snp_c1_4814
solcap_snp_c2_49976
solcap_snp_c2_36466
solcap_snp_c2_38058 solcap_snp_c2_38068
solcap_snp_c2_48364 solcap_snp_c2_37993
solcap_snp_c1_16679 solcap_snp_c2_37982
solcap_snp_c1_11339
solcap_snp_c1_7882 solcap_snp_c1_6869
solcap_snp_c1_6851 solcap_snp_c1_13059
solcap_snp_c1_9153 solcap_snp_c1_9161
solcap_snp_c2_45700 solcap_snp_c1_13843
solcap_snp_c2_13821
solcap_snp_c1_5766
solcap_snp_c2_20347
solcap_snp_c1_6425
solcap_snp_c2_55276 solcap_snp_c2_55277
solcap_snp_c2_55279 solcap_snp_c2_55283
solcap_snp_c1_8036 solcap_snp_c2_25560
solcap_snp_c2_2138 solcap_snp_c2_57863
solcap_snp_c2_57864 solcap_snp_c1_3638
solcap_snp_c2_29643 solcap_snp_c2_29639
solcap_snp_c2_54785
solcap_snp_c2_55072
solcap_snp_c2_20069
solcap_snp_c2_58296
solcap_snp_c1_432
solcap_snp_c2_1720
solcap_snp_c2_1844 solcap_snp_c2_47583
solcap_snp_c2_47510
solcap_snp_c1_4504
solcap_snp_c2_14064
solcap_snp_c2_14155
solcap_snp_c1_4576
solcap_snp_c2_17631 solcap_snp_c1_16513
solcap_snp_c1_5796
solcap_snp_c1_5799
solcap_snp_c2_47843
solcap_snp_c1_7076
solcap_snp_c1_7078
solcap_snp_c2_18273 solcap_snp_c2_18308
solcap_snp_c2_18428 solcap_snp_c2_18429
solcap_snp_c2_18503 solcap_snp_c2_18507
solcap_snp_c2_53258
solcap_snp_c2_26454 solcap_snp_c2_26417
solcap_snp_c2_26403 solcap_snp_c2_26382
solcap_snp_c2_391 solcap_snp_c2_387
solcap_snp_c2_328 solcap_snp_c1_97
solcap_snp_c2_308
solcap_snp_c2_245
solcap_snp_c2_86
solcap_snp_c2_68
solcap_snp_c1_151
solcap_snp_c2_164 solcap_snp_c2_578
solcap_snp_c2_9498 solcap_snp_c2_9531
solcap_snp_c2_37123 solcap_snp_c1_16472
solcap_snp_c1_10555 solcap_snp_c2_35572
solcap_snp_c1_11202
solcap_snp_c2_37414 solcap_snp_c1_11840
solcap_snp_c2_52815 solcap_snp_c1_10743
solcap_snp_c2_649 solcap_snp_c2_644
solcap_snp_c1_171 solcap_snp_c2_17218
solcap_snp_c1_156 solcap_snp_c1_200
solcap_snp_c1_450
solcap_snp_c2_11260
solcap_snp_c2_17279
solcap_snp_c2_50372
solcap_snp_c2_16679 solcap_snp_c2_16677
solcap_snp_c1_5388
solcap_snp_c2_41163
solcap_snp_c1_13651
solcap_snp_c2_41061
solcap_snp_c2_41071
solcap_snp_c2_51096 solcap_snp_c1_14973
solcap_snp_c1_14975
solcap_snp_c1_16382
solcap_snp_c2_43270 solcap_snp_c2_43267
solcap_snp_c2_43262 solcap_snp_c2_43255
solcap_snp_c1_12751
solcap_snp_c2_56834
solcap_snp_c2_35358
solcap_snp_c2_35353 solcap_snp_c2_35352
solcap_snp_c1_15925
solcap_snp_c1_15926
solcap_snp_c2_16571
solcap_snp_c2_49976
solcap_snp_c1_15343 solcap_snp_c1_7133
solcap_snp_c1_10879
solcap_snp_c1_11350
solcap_snp_c2_38047
solcap_snp_c2_38068
solcap_snp_c2_48371 solcap_snp_c2_48385
solcap_snp_c2_42306
solcap_snp_c2_42312 solcap_snp_c2_57349
solcap_snp_c1_13059
solcap_snp_c1_9153 solcap_snp_c1_9161
solcap_snp_c2_45698 solcap_snp_c2_45700
solcap_snp_c1_13847
solcap_snp_c2_46604
solcap_snp_c2_13825
solcap_snp_c1_13506 solcap_snp_c2_17581
solcap_snp_c1_5766
solcap_snp_c1_16094
solcap_snp_c1_6425
solcap_snp_c2_55277 solcap_snp_c2_55283
solcap_snp_c2_55285
solcap_snp_c1_8069
solcap_snp_c2_25654
solcap_snp_c2_25560
solcap_snp_c2_25662
solcap_snp_c2_57863
solcap_snp_c1_3638
solcap_snp_c2_55072
solcap_snp_c2_20077 solcap_snp_c2_20097
solcap_snp_c2_20170 solcap_snp_c2_20175
solcap_snp_c2_20179 solcap_snp_c1_6332
solcap_snp_c2_20259
solcap_snp_c2_20273
solcap_snp_c2_1556 solcap_snp_c1_378
solcap_snp_c2_1533
solcap_snp_c2_1835 solcap_snp_c2_1813
solcap_snp_c2_1720 solcap_snp_c2_1718
solcap_snp_c2_1717
solcap_snp_c2_1688
solcap_snp_c1_398
solcap_snp_c2_47510
solcap_snp_c1_4504 solcap_snp_c2_13987
solcap_snp_c2_14064
solcap_snp_c2_14155
solcap_snp_c1_4576 solcap_snp_c2_14258
solcap_snp_c2_17648
solcap_snp_c2_17631 solcap_snp_c1_16513
solcap_snp_c1_5796
solcap_snp_c2_47843
solcap_snp_c1_7076
solcap_snp_c1_7078
solcap_snp_c2_18268 solcap_snp_c2_18273
solcap_snp_c2_18428 solcap_snp_c2_18429
solcap_snp_c2_18463
solcap_snp_c2_18502
solcap_snp_c1_15547
solcap_snp_c2_26417
solcap_snp_c2_390 solcap_snp_c2_387
solcap_snp_c2_328 solcap_snp_c2_324
solcap_snp_c1_97 solcap_snp_c2_323
solcap_snp_c2_308
solcap_snp_c2_245
solcap_snp_c1_58 solcap_snp_c1_38
solcap_snp_c1_36
solcap_snp_c1_26 solcap_snp_c2_616
solcap_snp_c2_37137 solcap_snp_c2_37136
solcap_snp_c2_37118

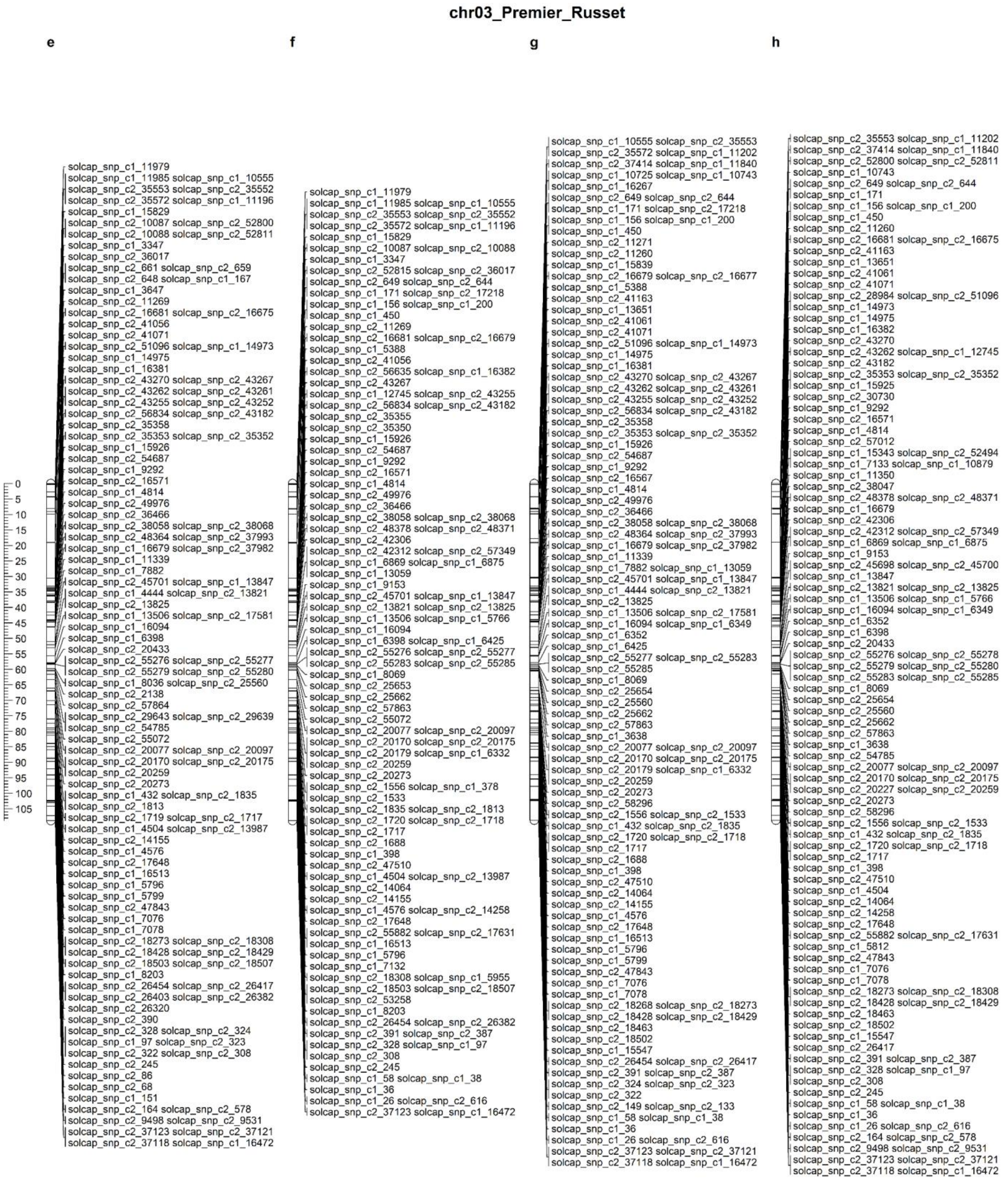
chr03_Premier_Russet
e
f
g
h
0
5
10
15
20
25
30
35
40
45
50
55
60
65
70
75
80
85
90
95
100
105
solcap_snp_c1_11979
solcap_snp_c1_11985 solcap_snp_c1_10555
solcap_snp_c2_35553 solcap_snp_c2_35552
solcap_snp_c2_35572 solcap_snp_c1_11196
solcap_snp_c1_15829
solcap_snp_c2_10087 solcap_snp_c2_52800
solcap_snp_c2_10088 solcap_snp_c2_52811
solcap_snp_c1_3347
solcap_snp_c2_36017
solcap_snp_c2_661 solcap_snp_c2_659
solcap_snp_c2_648 solcap_snp_c1_167
solcap_snp_c1_3647
solcap_snp_c2_11269
solcap_snp_c2_16681 solcap_snp_c2_16675
solcap_snp_c2_41056
solcap_snp_c2_41071
solcap_snp_c2_51096 solcap_snp_c1_14973
solcap_snp_c1_14975
solcap_snp_c1_16381
solcap_snp_c2_43270 solcap_snp_c2_43267
solcap_snp_c2_43262 solcap_snp_c2_43261
solcap_snp_c2_43255 solcap_snp_c2_43252
solcap_snp_c2_56834 solcap_snp_c2_43182
solcap_snp_c2_35358
solcap_snp_c2_35353 solcap_snp_c2_35352
solcap_snp_c1_15926
solcap_snp_c2_54687
solcap_snp_c1_9292
solcap_snp_c2_16571
solcap_snp_c1_4814
solcap_snp_c2_49976
solcap_snp_c2_36466
solcap_snp_c2_38058 solcap_snp_c2_38068
solcap_snp_c2_48364 solcap_snp_c2_37993
solcap_snp_c1_16679 solcap_snp_c2_37982
solcap_snp_c1_11339
solcap_snp_c1_7882
solcap_snp_c2_45701 solcap_snp_c1_13847
solcap_snp_c1_4444 solcap_snp_c2_13821
solcap_snp_c2_13825
solcap_snp_c1_13506 solcap_snp_c2_17581
solcap_snp_c1_16094
solcap_snp_c1_6398
solcap_snp_c2_20433
solcap_snp_c2_55276 solcap_snp_c2_55277
solcap_snp_c2_55279 solcap_snp_c2_55280
solcap_snp_c1_8036 solcap_snp_c2_25560
solcap_snp_c2_2138
solcap_snp_c2_57864
solcap_snp_c2_29643 solcap_snp_c2_29639
solcap_snp_c2_54785
solcap_snp_c2_55072
solcap_snp_c2_20077 solcap_snp_c2_20097
solcap_snp_c2_20170 solcap_snp_c2_20175
solcap_snp_c2_20259
solcap_snp_c2_20273
solcap_snp_c1_432 solcap_snp_c2_1835
solcap_snp_c2_1813
solcap_snp_c2_1719 solcap_snp_c2_1717
solcap_snp_c1_4504 solcap_snp_c2_13987
solcap_snp_c2_14155
solcap_snp_c1_4576
solcap_snp_c2_17648
solcap_snp_c1_16513
solcap_snp_c1_5796
solcap_snp_c1_5799
solcap_snp_c2_47843
solcap_snp_c1_7076
solcap_snp_c1_7078
solcap_snp_c2_18273 solcap_snp_c2_18308
solcap_snp_c2_18428 solcap_snp_c2_18429
solcap_snp_c2_18503 solcap_snp_c2_18507
solcap_snp_c1_8203
solcap_snp_c2_26454 solcap_snp_c2_26417
solcap_snp_c2_26403 solcap_snp_c2_26382
solcap_snp_c2_26320
solcap_snp_c2_390
solcap_snp_c2_328 solcap_snp_c2_324
solcap_snp_c1_97 solcap_snp_c2_323
solcap_snp_c2_322 solcap_snp_c2_308
solcap_snp_c2_245
solcap_snp_c2_86
solcap_snp_c2_68
solcap_snp_c1_151
solcap_snp_c2_164 solcap_snp_c2_578
solcap_snp_c2_9498 solcap_snp_c2_9531
solcap_snp_c2_37123 solcap_snp_c2_37121
solcap_snp_c2_37118 solcap_snp_c1_16472
solcap_snp_c1_11979
solcap_snp_c1_11985 solcap_snp_c1_10555
solcap_snp_c2_35553 solcap_snp_c2_35552
solcap_snp_c2_35572 solcap_snp_c1_11196
solcap_snp_c1_15829
solcap_snp_c2_10087 solcap_snp_c2_10088
solcap_snp_c1_3347
solcap_snp_c2_52815 solcap_snp_c2_36017
solcap_snp_c2_649 solcap_snp_c2_644
solcap_snp_c1_171 solcap_snp_c2_17218
solcap_snp_c1_156 solcap_snp_c1_200
solcap_snp_c1_450
solcap_snp_c2_11269
solcap_snp_c2_16681 solcap_snp_c2_16679
solcap_snp_c1_5388
solcap_snp_c2_41056
solcap_snp_c2_56635 solcap_snp_c1_16382
solcap_snp_c2_43267
solcap_snp_c1_12745 solcap_snp_c2_43255
solcap_snp_c2_56834 solcap_snp_c2_43182
solcap_snp_c2_35355
solcap_snp_c2_35350
solcap_snp_c1_15926
solcap_snp_c2_54687
solcap_snp_c1_9292
solcap_snp_c2_16571
solcap_snp_c1_4814
solcap_snp_c2_49976
solcap_snp_c2_36466
solcap_snp_c2_38058 solcap_snp_c2_38068
solcap_snp_c2_48378 solcap_snp_c2_48371
solcap_snp_c2_42306
solcap_snp_c2_42312 solcap_snp_c2_57349
solcap_snp_c1_6869 solcap_snp_c1_6875
solcap_snp_c1_13059
solcap_snp_c1_9153
solcap_snp_c2_45701 solcap_snp_c1_13847
solcap_snp_c2_13821 solcap_snp_c2_13825
solcap_snp_c1_13506 solcap_snp_c1_5766
solcap_snp_c1_16094
solcap_snp_c1_6398 solcap_snp_c1_6425
solcap_snp_c2_55276 solcap_snp_c2_55277
solcap_snp_c2_55283 solcap_snp_c2_55285
solcap_snp_c1_8069
solcap_snp_c2_25653
solcap_snp_c2_25662
solcap_snp_c2_57863
solcap_snp_c2_55072
solcap_snp_c2_20077 solcap_snp_c2_20097
solcap_snp_c2_20170 solcap_snp_c2_20175
solcap_snp_c2_20179 solcap_snp_c1_6332
solcap_snp_c2_20259
solcap_snp_c2_20273
solcap_snp_c2_1556 solcap_snp_c1_378
solcap_snp_c2_1533
solcap_snp_c2_1835 solcap_snp_c2_1813
solcap_snp_c2_1720 solcap_snp_c2_1718
solcap_snp_c2_1717
solcap_snp_c2_1688
solcap_snp_c1_398
solcap_snp_c2_47510
solcap_snp_c1_4504 solcap_snp_c2_13987
solcap_snp_c2_14064
solcap_snp_c2_14155
solcap_snp_c1_4576 solcap_snp_c2_14258
solcap_snp_c2_17648
solcap_snp_c2_55882 solcap_snp_c2_17631
solcap_snp_c1_16513
solcap_snp_c1_5796
solcap_snp_c1_7132
solcap_snp_c2_18308 solcap_snp_c1_5955
solcap_snp_c2_18503 solcap_snp_c2_18507
solcap_snp_c2_53258
solcap_snp_c1_8203
solcap_snp_c2_26454 solcap_snp_c2_26382
solcap_snp_c2_391 solcap_snp_c2_387
solcap_snp_c2_328 solcap_snp_c1_97
solcap_snp_c2_308
solcap_snp_c2_245
solcap_snp_c1_58 solcap_snp_c1_38
solcap_snp_c1_36
solcap_snp_c1_26 solcap_snp_c2_616
solcap_snp_c2_37123 solcap_snp_c1_16472
solcap_snp_c1_10555 solcap_snp_c2_35553
solcap_snp_c2_35572 solcap_snp_c1_11202
solcap_snp_c2_37414 solcap_snp_c1_11840
solcap_snp_c1_10725 solcap_snp_c1_10743
solcap_snp_c1_16267
solcap_snp_c2_649 solcap_snp_c2_644
solcap_snp_c1_171 solcap_snp_c2_17218
solcap_snp_c1_156 solcap_snp_c1_200
solcap_snp_c1_450
solcap_snp_c2_11271
solcap_snp_c2_11260
solcap_snp_c1_15839
solcap_snp_c2_16679 solcap_snp_c2_16677
solcap_snp_c1_5388
solcap_snp_c2_41163
solcap_snp_c1_13651
solcap_snp_c2_41061
solcap_snp_c2_41071
solcap_snp_c2_51096 solcap_snp_c1_14973
solcap_snp_c1_14975
solcap_snp_c1_16381
solcap_snp_c2_43270 solcap_snp_c2_43267
solcap_snp_c2_43262 solcap_snp_c2_43261
solcap_snp_c2_43255 solcap_snp_c2_43252
solcap_snp_c2_56834 solcap_snp_c2_43182
solcap_snp_c2_35358
solcap_snp_c2_35353 solcap_snp_c2_35352
solcap_snp_c1_15926
solcap_snp_c2_54687
solcap_snp_c1_9292
solcap_snp_c2_16567
solcap_snp_c1_4814
solcap_snp_c2_49976
solcap_snp_c2_36466
solcap_snp_c2_38058 solcap_snp_c2_38068
solcap_snp_c2_48364 solcap_snp_c2_37993
solcap_snp_c1_16679 solcap_snp_c2_37982
solcap_snp_c1_11339
solcap_snp_c1_7882 solcap_snp_c1_13059
solcap_snp_c2_45701 solcap_snp_c1_13847
solcap_snp_c1_4444 solcap_snp_c2_13821
solcap_snp_c2_13825
solcap_snp_c1_13506 solcap_snp_c2_17581
solcap_snp_c1_16094 solcap_snp_c1_6349
solcap_snp_c1_6352
solcap_snp_c1_6425
solcap_snp_c2_55277 solcap_snp_c2_55283
solcap_snp_c2_55285
solcap_snp_c1_8069
solcap_snp_c2_25654
solcap_snp_c2_25560
solcap_snp_c2_25662
solcap_snp_c2_57863
solcap_snp_c1_3638
solcap_snp_c2_20077 solcap_snp_c2_20097
solcap_snp_c2_20170 solcap_snp_c2_20175
solcap_snp_c2_20179 solcap_snp_c1_6332
solcap_snp_c2_20259
solcap_snp_c2_20273
solcap_snp_c2_58296
solcap_snp_c2_1556 solcap_snp_c2_1533
solcap_snp_c1_432 solcap_snp_c2_1835
solcap_snp_c2_1720 solcap_snp_c2_1718
solcap_snp_c2_1717
solcap_snp_c2_1688
solcap_snp_c1_398
solcap_snp_c2_47510
solcap_snp_c2_14064
solcap_snp_c2_14155
solcap_snp_c1_4576
solcap_snp_c2_17648
solcap_snp_c1_16513
solcap_snp_c1_5796
solcap_snp_c1_5799
solcap_snp_c2_47843
solcap_snp_c1_7076
solcap_snp_c1_7078
solcap_snp_c2_18268 solcap_snp_c2_18273
solcap_snp_c2_18428 solcap_snp_c2_18429
solcap_snp_c2_18463
solcap_snp_c2_18502
solcap_snp_c1_15547
solcap_snp_c2_26454 solcap_snp_c2_26417
solcap_snp_c2_391 solcap_snp_c2_387
solcap_snp_c2_324 solcap_snp_c2_323
solcap_snp_c2_322
solcap_snp_c2_149 solcap_snp_c2_133
solcap_snp_c1_58 solcap_snp_c1_38
solcap_snp_c1_36
solcap_snp_c1_26 solcap_snp_c2_616
solcap_snp_c2_37123 solcap_snp_c2_37121
solcap_snp_c2_37118 solcap_snp_c1_16472
solcap_snp_c2_35553 solcap_snp_c1_11202
solcap_snp_c2_37414 solcap_snp_c1_11840
solcap_snp_c2_52800 solcap_snp_c2_52811
solcap_snp_c1_10743
solcap_snp_c2_649 solcap_snp_c2_644
solcap_snp_c1_171
solcap_snp_c1_156 solcap_snp_c1_200
solcap_snp_c1_450
solcap_snp_c2_11260
solcap_snp_c2_16681 solcap_snp_c2_16675
solcap_snp_c2_41163
solcap_snp_c1_13651
solcap_snp_c2_41061
solcap_snp_c2_41071
solcap_snp_c2_28984 solcap_snp_c2_51096
solcap_snp_c1_14973
solcap_snp_c1_14975
solcap_snp_c1_16382
solcap_snp_c2_43270
solcap_snp_c2_43262 solcap_snp_c1_12745
solcap_snp_c2_43182
solcap_snp_c2_35353 solcap_snp_c2_35352
solcap_snp_c1_15925
solcap_snp_c2_30730
solcap_snp_c1_9292
solcap_snp_c2_16571
solcap_snp_c1_4814
solcap_snp_c2_57012
solcap_snp_c1_15343 solcap_snp_c2_52494
solcap_snp_c1_7133 solcap_snp_c1_10879
solcap_snp_c1_11350
solcap_snp_c2_38047
solcap_snp_c2_48378 solcap_snp_c2_48371
solcap_snp_c1_16679
solcap_snp_c2_42306
solcap_snp_c2_42312 solcap_snp_c2_57349
solcap_snp_c1_6869 solcap_snp_c1_6875
solcap_snp_c1_9153
solcap_snp_c2_45698 solcap_snp_c2_45700
solcap_snp_c1_13847
solcap_snp_c2_13821 solcap_snp_c2_13825
solcap_snp_c1_13506 solcap_snp_c1_5766
solcap_snp_c1_16094 solcap_snp_c1_6349
solcap_snp_c1_6352
solcap_snp_c1_6398
solcap_snp_c2_20433
solcap_snp_c2_55276 solcap_snp_c2_55278
solcap_snp_c2_55279 solcap_snp_c2_55280
solcap_snp_c2_55283 solcap_snp_c2_55285
solcap_snp_c1_8069
solcap_snp_c2_25654
solcap_snp_c2_25560
solcap_snp_c2_25662
solcap_snp_c2_57863
solcap_snp_c1_3638
solcap_snp_c2_54785
solcap_snp_c2_20077 solcap_snp_c2_20097
solcap_snp_c2_20170 solcap_snp_c2_20175
solcap_snp_c2_20227 solcap_snp_c2_20259
solcap_snp_c2_20273
solcap_snp_c2_58296
solcap_snp_c2_1556 solcap_snp_c2_1533
solcap_snp_c1_432 solcap_snp_c2_1835
solcap_snp_c2_1720 solcap_snp_c2_1718
solcap_snp_c2_1717
solcap_snp_c1_398
solcap_snp_c2_47510
solcap_snp_c1_4504
solcap_snp_c2_14064
solcap_snp_c2_14258
solcap_snp_c2_17648
solcap_snp_c2_55882 solcap_snp_c2_17631
solcap_snp_c1_5812
solcap_snp_c2_47843
solcap_snp_c1_7076
solcap_snp_c1_7078
solcap_snp_c2_18273 solcap_snp_c2_18308
solcap_snp_c2_18428 solcap_snp_c2_18429
solcap_snp_c2_18463
solcap_snp_c2_18502
solcap_snp_c1_15547
solcap_snp_c2_26417
solcap_snp_c2_391 solcap_snp_c2_387
solcap_snp_c2_328 solcap_snp_c1_97
solcap_snp_c2_308
solcap_snp_c2_245
solcap_snp_c1_58 solcap_snp_c1_38
solcap_snp_c1_36
solcap_snp_c1_26 solcap_snp_c2_616
solcap_snp_c2_164 solcap_snp_c2_578
solcap_snp_c2_9498 solcap_snp_c2_9531
solcap_snp_c2_37123 solcap_snp_c2_37121
solcap_snp_c2_37118 solcap_snp_c1_16472

chr04_Rio_Grande_Russet

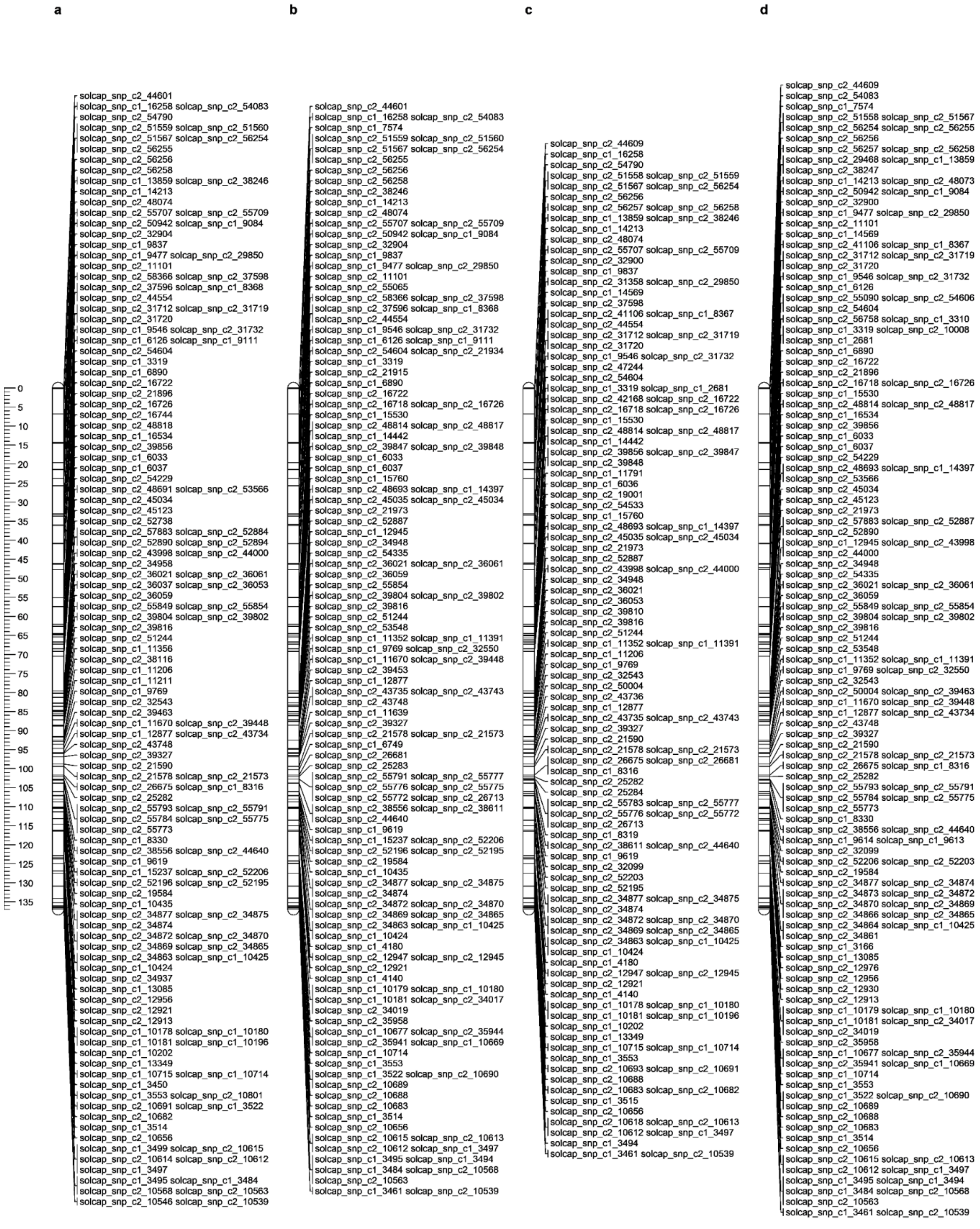

chr04_Premier_Russet

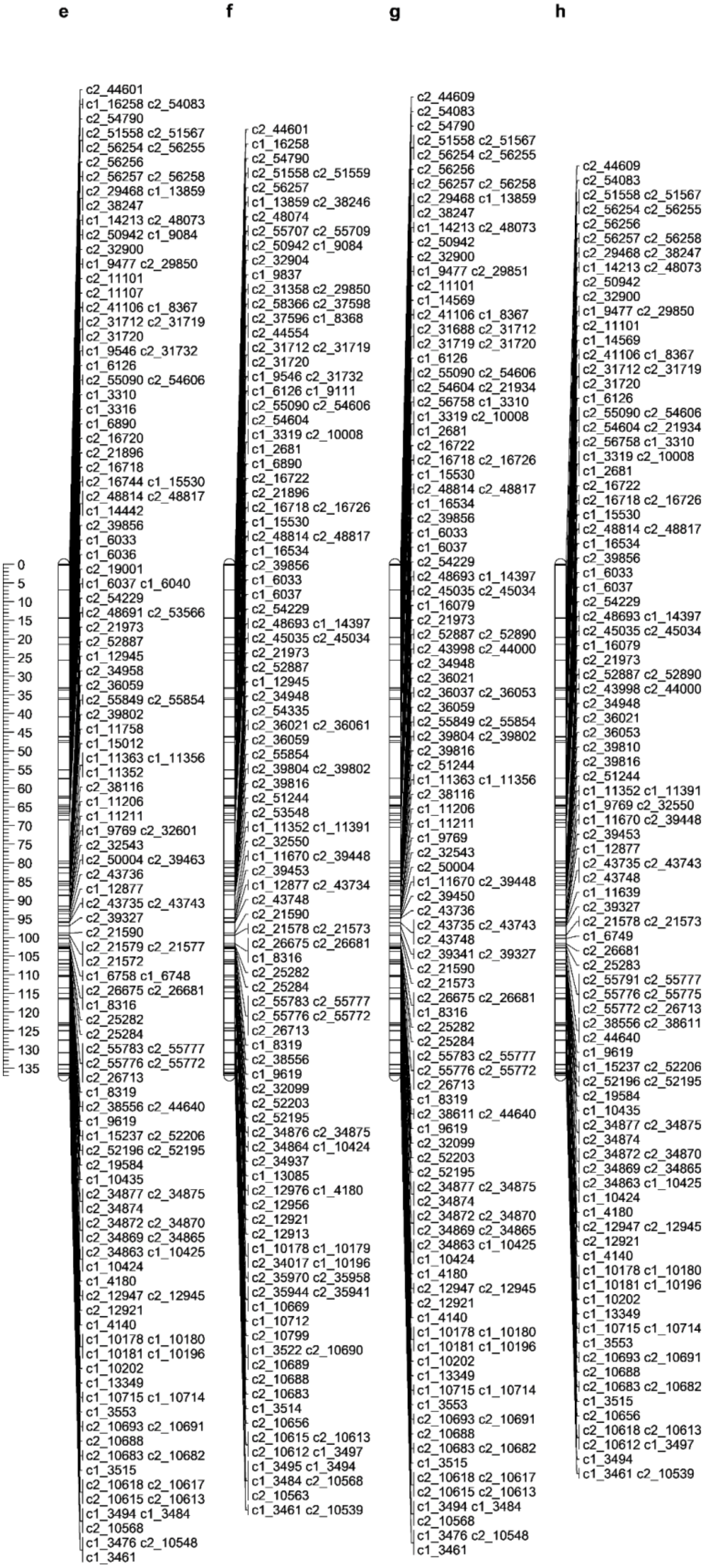

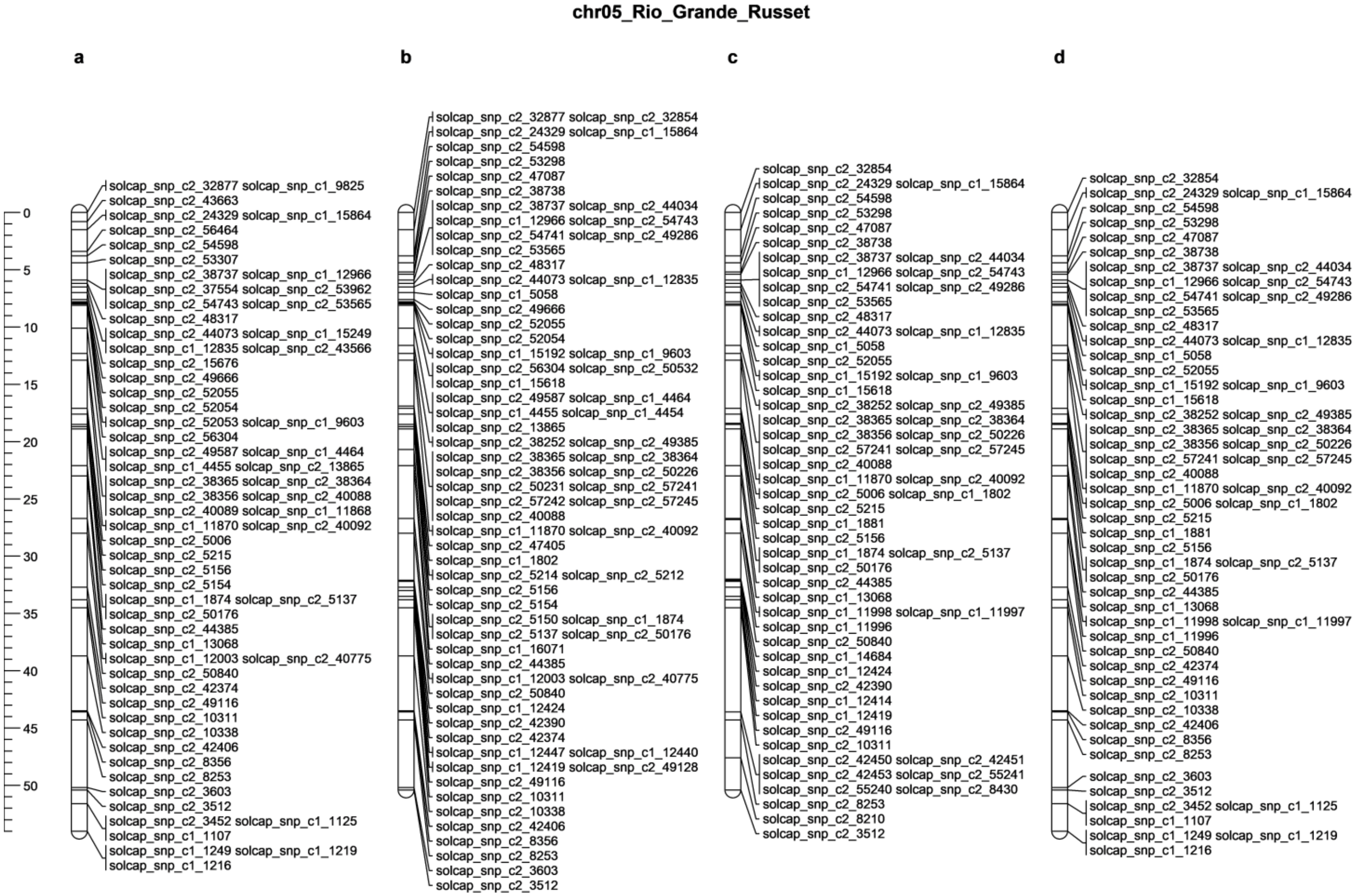

chr05_Rio_Grande_Russet
a
b
c
d
0
5
10
15
20
25
30
35
40
45
50
solcap_snp_c2_32877 solcap_snp_c1_9825
solcap_snp_c2_43663
solcap_snp_c2_24329 solcap_snp_c1_15864
solcap_snp_c2_56464
solcap_snp_c2_54598
solcap_snp_c2_53307
solcap_snp_c2_38737 solcap_snp_c1_12966
solcap_snp_c2_37554 solcap_snp_c2_53962
solcap_snp_c2_54743 solcap_snp_c2_53565
solcap_snp_c2_48317
solcap_snp_c2_44073 solcap_snp_c1_15249
solcap_snp_c1_12835 solcap_snp_c2_43566
solcap_snp_c2_15676
solcap_snp_c2_49666
solcap_snp_c2_52055
solcap_snp_c2_52054
solcap_snp_c2_52053 solcap_snp_c1_9603
solcap_snp_c2_56304
solcap_snp_c2_49587 solcap_snp_c1_4464
solcap_snp_c1_4455 solcap_snp_c2_13865
solcap_snp_c2_38365 solcap_snp_c2_38364
solcap_snp_c2_38356 solcap_snp_c2_40088
solcap_snp_c2_40089 solcap_snp_c1_11868
solcap_snp_c1_11870 solcap_snp_c2_40092
solcap_snp_c2_5006
solcap_snp_c2_5215
solcap_snp_c2_5156
solcap_snp_c2_5154
solcap_snp_c1_1874 solcap_snp_c2_5137
solcap_snp_c2_50176
solcap_snp_c2_44385
solcap_snp_c1_13068
solcap_snp_c1_12003 solcap_snp_c2_40775
solcap_snp_c2_50840
solcap_snp_c2_42374
solcap_snp_c2_49116
solcap_snp_c2_10311
solcap_snp_c2_10338
solcap_snp_c2_42406
solcap_snp_c2_8356
solcap_snp_c2_8253
solcap_snp_c2_3603
solcap_snp_c2_3512
solcap_snp_c2_3452 solcap_snp_c1_1125
solcap_snp_c1_1107
solcap_snp_c1_1249 solcap_snp_c1_1219
solcap_snp_c1_1216
solcap_snp_c2_32877 solcap_snp_c2_32854
solcap_snp_c2_24329 solcap_snp_c1_15864
solcap_snp_c2_54598
solcap_snp_c2_53298
solcap_snp_c2_47087
solcap_snp_c2_38738
solcap_snp_c2_38737 solcap_snp_c2_44034
solcap_snp_c1_12966 solcap_snp_c2_54743
solcap_snp_c2_54741 solcap_snp_c2_49286
solcap_snp_c2_53565
solcap_snp_c2_48317
solcap_snp_c2_44073 solcap_snp_c1_12835
solcap_snp_c1_5058
solcap_snp_c2_49666
solcap_snp_c2_52055
solcap_snp_c2_52054
solcap_snp_c1_15192 solcap_snp_c1_9603
solcap_snp_c2_56304 solcap_snp_c2_50532
solcap_snp_c1_15618
solcap_snp_c2_49587 solcap_snp_c1_4464
solcap_snp_c1_4455 solcap_snp_c1_4454
solcap_snp_c2_13865
solcap_snp_c2_38252 solcap_snp_c2_49385
solcap_snp_c2_38365 solcap_snp_c2_38364
solcap_snp_c2_38356 solcap_snp_c2_50226
solcap_snp_c2_50231 solcap_snp_c2_57241
solcap_snp_c2_57242 solcap_snp_c2_57245
solcap_snp_c2_40088
solcap_snp_c1_11870 solcap_snp_c2_40092
solcap_snp_c2_47405
solcap_snp_c1_1802
solcap_snp_c2_5214 solcap_snp_c2_5212
solcap_snp_c2_5156
solcap_snp_c2_5154
solcap_snp_c2_5150 solcap_snp_c1_1874
solcap_snp_c2_5137 solcap_snp_c2_50176
solcap_snp_c1_16071
solcap_snp_c2_44385
solcap_snp_c1_12003 solcap_snp_c2_40775
solcap_snp_c2_50840
solcap_snp_c1_12424
solcap_snp_c2_42390
solcap_snp_c2_42374
solcap_snp_c1_12447 solcap_snp_c1_12440
solcap_snp_c1_12419 solcap_snp_c2_49128
solcap_snp_c2_49116
solcap_snp_c2_10311
solcap_snp_c2_10338
solcap_snp_c2_42406
solcap_snp_c2_8356
solcap_snp_c2_8253
solcap_snp_c2_3603
solcap_snp_c2_3512
solcap_snp_c2_32854
solcap_snp_c2_24329 solcap_snp_c1_15864
solcap_snp_c2_54598
solcap_snp_c2_53298
solcap_snp_c2_47087
solcap_snp_c2_38738
solcap_snp_c2_38737 solcap_snp_c2_44034
solcap_snp_c1_12966 solcap_snp_c2_54743
solcap_snp_c2_54741 solcap_snp_c2_49286
solcap_snp_c2_53565
solcap_snp_c2_48317
solcap_snp_c2_44073 solcap_snp_c1_12835
solcap_snp_c1_5058
solcap_snp_c2_52055
solcap_snp_c1_15192 solcap_snp_c1_9603
solcap_snp_c1_15618
solcap_snp_c2_38252 solcap_snp_c2_49385
solcap_snp_c2_38365 solcap_snp_c2_38364
solcap_snp_c2_38356 solcap_snp_c2_50226
solcap_snp_c2_57241 solcap_snp_c2_57245
solcap_snp_c2_40088
solcap_snp_c1_11870 solcap_snp_c2_40092
solcap_snp_c2_5006 solcap_snp_c1_1802
solcap_snp_c2_5215
solcap_snp_c1_1881
solcap_snp_c2_5156
solcap_snp_c1_1874 solcap_snp_c2_5137
solcap_snp_c2_50176
solcap_snp_c2_44385
solcap_snp_c1_13068
solcap_snp_c1_11998 solcap_snp_c1_11997
solcap_snp_c1_11996
solcap_snp_c2_50840
solcap_snp_c1_14684
solcap_snp_c1_12424
solcap_snp_c2_42390
solcap_snp_c1_12414
solcap_snp_c1_12419
solcap_snp_c2_49116
solcap_snp_c2_10311
solcap_snp_c2_42450 solcap_snp_c2_42451
solcap_snp_c2_42453 solcap_snp_c2_55241
solcap_snp_c2_55240 solcap_snp_c2_8430
solcap_snp_c2_8253
solcap_snp_c2_8210
solcap_snp_c2_3512
solcap_snp_c2_32854
solcap_snp_c2_24329 solcap_snp_c1_15864
solcap_snp_c2_54598
solcap_snp_c2_53298
solcap_snp_c2_47087
solcap_snp_c2_38738
solcap_snp_c2_38737 solcap_snp_c2_44034
solcap_snp_c1_12966 solcap_snp_c2_54743
solcap_snp_c2_54741 solcap_snp_c2_49286
solcap_snp_c2_53565
solcap_snp_c2_48317
solcap_snp_c2_44073 solcap_snp_c1_12835
solcap_snp_c1_5058
solcap_snp_c2_52055
solcap_snp_c1_15192 solcap_snp_c1_9603
solcap_snp_c1_15618
solcap_snp_c2_38252 solcap_snp_c2_49385
solcap_snp_c2_38365 solcap_snp_c2_38364
solcap_snp_c2_38356 solcap_snp_c2_50226
solcap_snp_c2_57241 solcap_snp_c2_57245
solcap_snp_c2_40088
solcap_snp_c1_11870 solcap_snp_c2_40092
solcap_snp_c2_5006 solcap_snp_c1_1802
solcap_snp_c2_5215
solcap_snp_c1_1881
solcap_snp_c2_5156
solcap_snp_c1_1874 solcap_snp_c2_5137
solcap_snp_c2_50176
solcap_snp_c2_44385
solcap_snp_c1_13068
solcap_snp_c1_11998 solcap_snp_c1_11997
solcap_snp_c1_11996
solcap_snp_c2_50840
solcap_snp_c2_42374
solcap_snp_c2_49116
solcap_snp_c2_10311
solcap_snp_c2_10338
solcap_snp_c2_42406
solcap_snp_c2_8356
solcap_snp_c2_8253
solcap_snp_c2_3603
solcap_snp_c2_3512
solcap_snp_c2_3452 solcap_snp_c1_1125
solcap_snp_c1_1107
solcap_snp_c1_1249 solcap_snp_c1_1219
solcap_snp_c1_1216

chr05_Premier_Russet

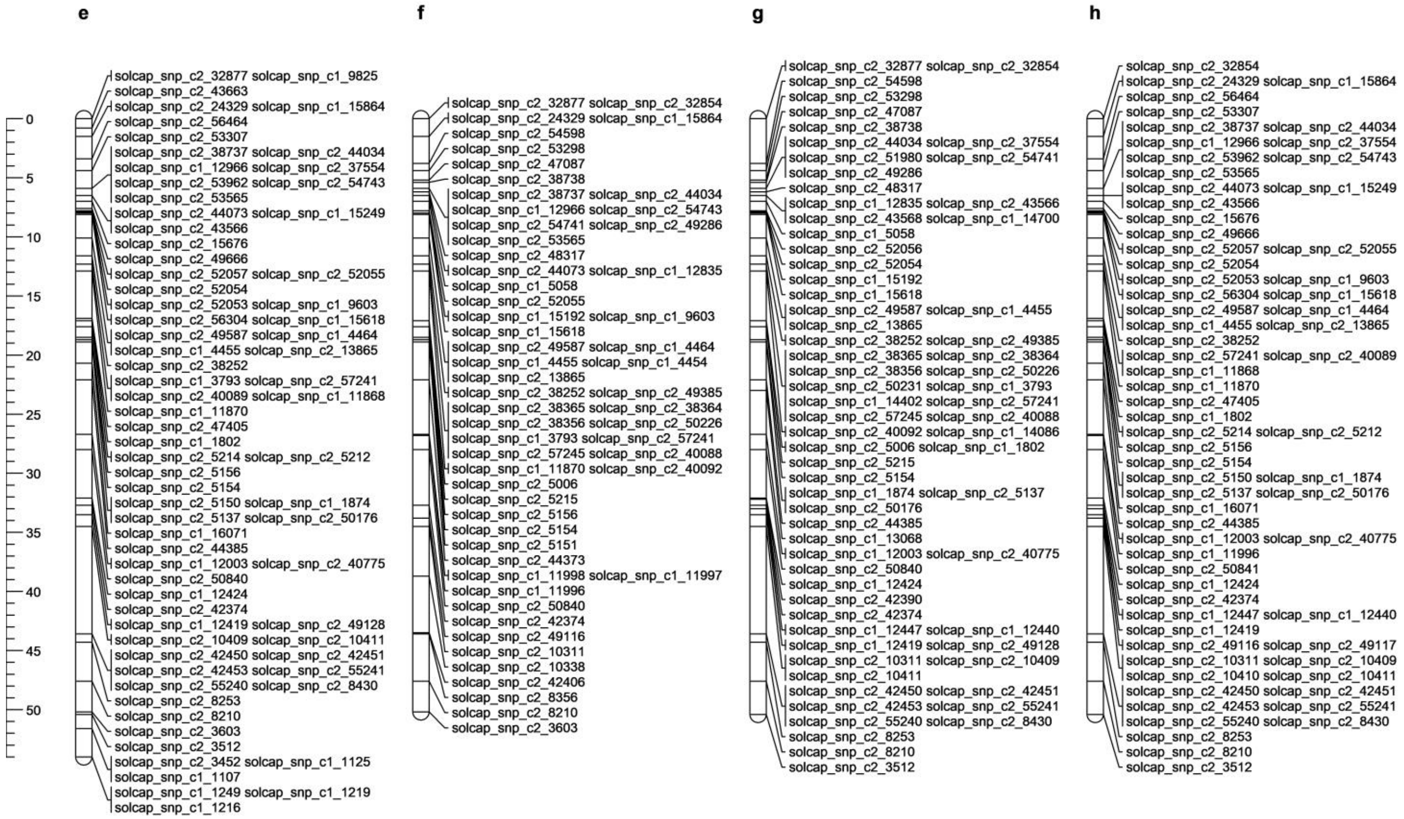

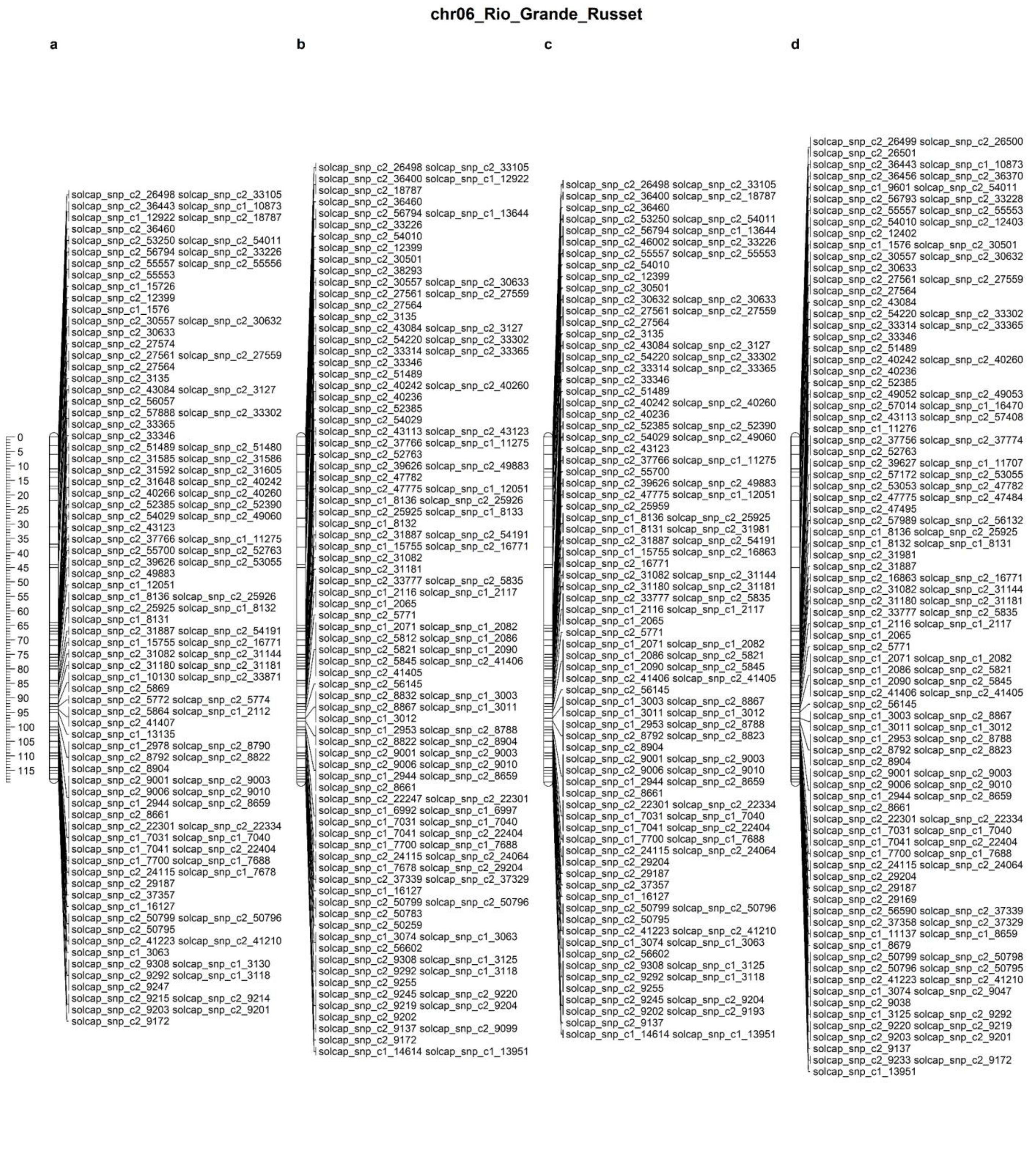
chr06_Rio_Grande_Russet
a
b
c
d
0
5
10
15
20
25
30
35
40
45
50
55
60
65
70
75
80
85
90
95
100
105
110
115
solcap_snp_c2_26498 solcap_snp_c2_33105
solcap_snp_c2_36443 solcap_snp_c1_10873
solcap_snp_c1_12922 solcap_snp_c2_18787
solcap_snp_c2_36460
solcap_snp_c2_53250 solcap_snp_c2_54011
solcap_snp_c2_56794 solcap_snp_c2_33226
solcap_snp_c2_55557 solcap_snp_c2_55556
solcap_snp_c2_55553
solcap_snp_c1_15726
solcap_snp_c2_12399
solcap_snp_c1_1576
solcap_snp_c2_30557 solcap_snp_c2_30632
solcap_snp_c2_30633
solcap_snp_c2_27574
solcap_snp_c2_27561 solcap_snp_c2_27559
solcap_snp_c2_27564
solcap_snp_c2_3135
solcap_snp_c2_43084 solcap_snp_c2_3127
solcap_snp_c2_56057
solcap_snp_c2_57888 solcap_snp_c2_33302
solcap_snp_c2_33365
solcap_snp_c2_33346
solcap_snp_c2_51489 solcap_snp_c2_51480
solcap_snp_c2_31585 solcap_snp_c2_31586
solcap_snp_c2_31592 solcap_snp_c2_31605
solcap_snp_c2_31648 solcap_snp_c2_40242
solcap_snp_c2_40266 solcap_snp_c2_40260
solcap_snp_c2_52385 solcap_snp_c2_52390
solcap_snp_c2_54029 solcap_snp_c2_49060
solcap_snp_c2_43123
solcap_snp_c2_37766 solcap_snp_c1_11275
solcap_snp_c2_55700 solcap_snp_c2_52763
solcap_snp_c2_39626 solcap_snp_c2_53055
solcap_snp_c2_49883
solcap_snp_c1_12051
solcap_snp_c1_8136 solcap_snp_c2_25926
solcap_snp_c2_25925 solcap_snp_c1_8132
solcap_snp_c1_8131
solcap_snp_c2_31887 solcap_snp_c2_54191
solcap_snp_c1_15755 solcap_snp_c2_16771
solcap_snp_c2_31082 solcap_snp_c2_31144
solcap_snp_c2_31180 solcap_snp_c2_31181
solcap_snp_c1_10130 solcap_snp_c2_33871
solcap_snp_c2_5869
solcap_snp_c2_5772 solcap_snp_c2_5774
solcap_snp_c2_5864 solcap_snp_c1_2112
solcap_snp_c2_41407
solcap_snp_c1_13135
solcap_snp_c1_2978 solcap_snp_c2_8790
solcap_snp_c2_8792 solcap_snp_c2_8822
solcap_snp_c2_8904
solcap_snp_c2_9001 solcap_snp_c2_9003
solcap_snp_c2_9006 solcap_snp_c2_9010
solcap_snp_c1_2944 solcap_snp_c2_8659
solcap_snp_c2_8661
solcap_snp_c2_22301 solcap_snp_c2_22334
solcap_snp_c1_7031 solcap_snp_c1_7040
solcap_snp_c1_7041 solcap_snp_c2_22404
solcap_snp_c1_7700 solcap_snp_c1_7688
solcap_snp_c2_24115 solcap_snp_c1_7678
solcap_snp_c2_29187
solcap_snp_c2_37357
solcap_snp_c1_16127
solcap_snp_c2_50799 solcap_snp_c2_50796
solcap_snp_c2_50795
solcap_snp_c2_41223 solcap_snp_c2_41210
solcap_snp_c1_3063
solcap_snp_c2_9308 solcap_snp_c1_3130
solcap_snp_c2_9292 solcap_snp_c1_3118
solcap_snp_c2_9247
solcap_snp_c2_9215 solcap_snp_c2_9214
solcap_snp_c2_9203 solcap_snp_c2_9201
solcap_snp_c2_9172
solcap_snp_c2_26498 solcap_snp_c2_33105
solcap_snp_c2_36400 solcap_snp_c1_12922
solcap_snp_c2_18787
solcap_snp_c2_36460
solcap_snp_c2_56794 solcap_snp_c1_13644
solcap_snp_c2_33226
solcap_snp_c2_54010
solcap_snp_c2_12399
solcap_snp_c2_30501
solcap_snp_c2_38293
solcap_snp_c2_30557 solcap_snp_c2_30633
solcap_snp_c2_27561 solcap_snp_c2_27559
solcap_snp_c2_27564
solcap_snp_c2_3135
solcap_snp_c2_43084 solcap_snp_c2_3127
solcap_snp_c2_54220 solcap_snp_c2_33302
solcap_snp_c2_33314 solcap_snp_c2_33365
solcap_snp_c2_33346
solcap_snp_c2_51489
solcap_snp_c2_40242 solcap_snp_c2_40260
solcap_snp_c2_40236
solcap_snp_c2_52385
solcap_snp_c2_54029
solcap_snp_c2_43113 solcap_snp_c2_43123
solcap_snp_c2_37766 solcap_snp_c1_11275
solcap_snp_c2_52763
solcap_snp_c2_39626 solcap_snp_c2_49883
solcap_snp_c2_47782
solcap_snp_c2_47775 solcap_snp_c1_12051
solcap_snp_c1_8136 solcap_snp_c2_25926
solcap_snp_c2_25925 solcap_snp_c1_8133
solcap_snp_c1_8132
solcap_snp_c2_31887 solcap_snp_c2_54191
solcap_snp_c1_15755 solcap_snp_c2_16771
solcap_snp_c2_31082
solcap_snp_c2_31181
solcap_snp_c2_33777 solcap_snp_c2_5835
solcap_snp_c1_2116 solcap_snp_c1_2117
solcap_snp_c1_2065
solcap_snp_c2_5771
solcap_snp_c1_2071 solcap_snp_c1_2082
solcap_snp_c2_5812 solcap_snp_c1_2086
solcap_snp_c2_5821 solcap_snp_c1_2090
solcap_snp_c2_5845 solcap_snp_c2_41406
solcap_snp_c2_41405
solcap_snp_c2_56145
solcap_snp_c2_8832 solcap_snp_c1_3003
solcap_snp_c2_8867 solcap_snp_c1_3011
solcap_snp_c1_3012
solcap_snp_c1_2953 solcap_snp_c2_8788
solcap_snp_c2_8822 solcap_snp_c2_8904
solcap_snp_c2_9001 solcap_snp_c2_9003
solcap_snp_c2_9006 solcap_snp_c2_9010
solcap_snp_c1_2944 solcap_snp_c2_8659
solcap_snp_c2_8661
solcap_snp_c2_22247 solcap_snp_c2_22301
solcap_snp_c1_6992 solcap_snp_c1_6997
solcap_snp_c1_7031 solcap_snp_c1_7040
solcap_snp_c1_7041 solcap_snp_c2_22404
solcap_snp_c1_7700 solcap_snp_c1_7688
solcap_snp_c2_24115 solcap_snp_c2_24064
solcap_snp_c1_7678 solcap_snp_c2_29204
solcap_snp_c2_37339 solcap_snp_c2_37329
solcap_snp_c1_16127
solcap_snp_c2_50799 solcap_snp_c2_50796
solcap_snp_c2_50783
solcap_snp_c2_50259
solcap_snp_c1_3074 solcap_snp_c1_3063
solcap_snp_c2_56602
solcap_snp_c2_9308 solcap_snp_c1_3125
solcap_snp_c2_9292 solcap_snp_c1_3118
solcap_snp_c2_9255
solcap_snp_c2_9245 solcap_snp_c2_9220
solcap_snp_c2_9219 solcap_snp_c2_9204
solcap_snp_c2_9202
solcap_snp_c2_9137 solcap_snp_c2_9099
solcap_snp_c2_9172
solcap_snp_c1_14614 solcap_snp_c1_13951
solcap_snp_c2_26498 solcap_snp_c2_33105
solcap_snp_c2_36400 solcap_snp_c2_18787
solcap_snp_c2_36460
solcap_snp_c2_53250 solcap_snp_c2_54011
solcap_snp_c2_56794 solcap_snp_c1_13644
solcap_snp_c2_46002 solcap_snp_c2_33226
solcap_snp_c2_55557 solcap_snp_c2_55553
solcap_snp_c2_54010
solcap_snp_c2_12399
solcap_snp_c2_30501
solcap_snp_c2_30632 solcap_snp_c2_30633
solcap_snp_c2_27561 solcap_snp_c2_27559
solcap_snp_c2_27564
solcap_snp_c2_3135
solcap_snp_c2_43084 solcap_snp_c2_3127
solcap_snp_c2_54220 solcap_snp_c2_33302
solcap_snp_c2_33314 solcap_snp_c2_33365
solcap_snp_c2_33346
solcap_snp_c2_51489
solcap_snp_c2_40242 solcap_snp_c2_40260
solcap_snp_c2_40236
solcap_snp_c2_52385 solcap_snp_c2_52390
solcap_snp_c2_54029 solcap_snp_c2_49060
solcap_snp_c2_43123
solcap_snp_c2_37766 solcap_snp_c1_11275
solcap_snp_c2_55700
solcap_snp_c2_39626 solcap_snp_c2_49883
solcap_snp_c2_47775 solcap_snp_c1_12051
solcap_snp_c2_25959
solcap_snp_c1_8136 solcap_snp_c2_25925
solcap_snp_c1_8131 solcap_snp_c2_31981
solcap_snp_c2_31887 solcap_snp_c2_54191
solcap_snp_c1_15755 solcap_snp_c2_16863
solcap_snp_c2_16771
solcap_snp_c2_31082 solcap_snp_c2_31144
solcap_snp_c2_31180 solcap_snp_c2_31181
solcap_snp_c2_33777 solcap_snp_c2_5835
solcap_snp_c1_2116 solcap_snp_c1_2117
solcap_snp_c1_2065
solcap_snp_c2_5771
solcap_snp_c1_2071 solcap_snp_c1_2082
solcap_snp_c1_2086 solcap_snp_c2_5821
solcap_snp_c1_2090 solcap_snp_c2_5845
solcap_snp_c2_41406 solcap_snp_c2_41405
solcap_snp_c2_56145
solcap_snp_c1_3003 solcap_snp_c2_8867
solcap_snp_c1_3011 solcap_snp_c1_3012
solcap_snp_c1_2953 solcap_snp_c2_8788
solcap_snp_c2_8792 solcap_snp_c2_8823
solcap_snp_c2_8904
solcap_snp_c2_9001 solcap_snp_c2_9003
solcap_snp_c2_9006 solcap_snp_c2_9010
solcap_snp_c1_2944 solcap_snp_c2_8659
solcap_snp_c2_8661
solcap_snp_c2_22301 solcap_snp_c2_22334
solcap_snp_c1_7031 solcap_snp_c1_7040
solcap_snp_c1_7041 solcap_snp_c2_22404
solcap_snp_c1_7700 solcap_snp_c1_7688
solcap_snp_c2_24115 solcap_snp_c2_24064
solcap_snp_c2_29204
solcap_snp_c2_29187
solcap_snp_c2_37357
solcap_snp_c1_16127
solcap_snp_c2_50799 solcap_snp_c2_50796
solcap_snp_c2_50795
solcap_snp_c2_41223 solcap_snp_c2_41210
solcap_snp_c1_3074 solcap_snp_c1_3063
solcap_snp_c2_56602
solcap_snp_c2_9308 solcap_snp_c1_3125
solcap_snp_c2_9292 solcap_snp_c1_3118
solcap_snp_c2_9255
solcap_snp_c2_9245 solcap_snp_c2_9204
solcap_snp_c2_9202 solcap_snp_c2_9193
solcap_snp_c2_9137
solcap_snp_c1_14614 solcap_snp_c1_13951
solcap_snp_c2_26499 solcap_snp_c2_26500
solcap_snp_c2_26501
solcap_snp_c2_36443 solcap_snp_c1_10873
solcap_snp_c2_36456 solcap_snp_c2_36370
solcap_snp_c1_9601 solcap_snp_c2_54011
solcap_snp_c2_56793 solcap_snp_c2_33228
solcap_snp_c2_55557 solcap_snp_c2_55553
solcap_snp_c2_54010 solcap_snp_c2_12403
solcap_snp_c2_12402
solcap_snp_c1_1576 solcap_snp_c2_30501
solcap_snp_c2_30557 solcap_snp_c2_30632
solcap_snp_c2_30633
solcap_snp_c2_27561 solcap_snp_c2_27559
solcap_snp_c2_27564
solcap_snp_c2_43084
solcap_snp_c2_54220 solcap_snp_c2_33302
solcap_snp_c2_33314 solcap_snp_c2_33365
solcap_snp_c2_33346
solcap_snp_c2_51489
solcap_snp_c2_40242 solcap_snp_c2_40260
solcap_snp_c2_40236
solcap_snp_c2_52385
solcap_snp_c2_49052 solcap_snp_c2_49053
solcap_snp_c2_57014 solcap_snp_c1_16470
solcap_snp_c2_43113 solcap_snp_c2_57408
solcap_snp_c1_11276
solcap_snp_c2_37756 solcap_snp_c2_37774
solcap_snp_c2_52763
solcap_snp_c2_39627 solcap_snp_c1_11707
solcap_snp_c2_57172 solcap_snp_c2_53055
solcap_snp_c2_53053 solcap_snp_c2_47782
solcap_snp_c2_47775 solcap_snp_c2_47484
solcap_snp_c2_47495
solcap_snp_c2_57989 solcap_snp_c2_56132
solcap_snp_c1_8136 solcap_snp_c2_25925
solcap_snp_c1_8132 solcap_snp_c1_8131
solcap_snp_c2_31981
solcap_snp_c2_31887
solcap_snp_c2_16863 solcap_snp_c2_16771
solcap_snp_c2_31082 solcap_snp_c2_31144
solcap_snp_c2_31180 solcap_snp_c2_31181
solcap_snp_c2_33777 solcap_snp_c2_5835
solcap_snp_c1_2116 solcap_snp_c1_2117
solcap_snp_c1_2065
solcap_snp_c2_5771
solcap_snp_c1_2071 solcap_snp_c1_2082
solcap_snp_c1_2086 solcap_snp_c2_5821
solcap_snp_c1_2090 solcap_snp_c2_5845
solcap_snp_c2_41406 solcap_snp_c2_41405
solcap_snp_c2_56145
solcap_snp_c1_3003 solcap_snp_c2_8867
solcap_snp_c1_3011 solcap_snp_c1_3012
solcap_snp_c1_2953 solcap_snp_c2_8788
solcap_snp_c2_8792 solcap_snp_c2_8823
solcap_snp_c2_8904
solcap_snp_c2_9001 solcap_snp_c2_9003
solcap_snp_c2_9006 solcap_snp_c2_9010
solcap_snp_c1_2944 solcap_snp_c2_8659
solcap_snp_c2_8661
solcap_snp_c2_22301 solcap_snp_c2_22334
solcap_snp_c1_7031 solcap_snp_c1_7040
solcap_snp_c1_7041 solcap_snp_c2_22404
solcap_snp_c1_7700 solcap_snp_c1_7688
solcap_snp_c2_24115 solcap_snp_c2_24064
solcap_snp_c2_29204
solcap_snp_c2_29187
solcap_snp_c2_29169
solcap_snp_c2_56590 solcap_snp_c2_37339
solcap_snp_c2_37358 solcap_snp_c2_37329
solcap_snp_c1_11137 solcap_snp_c1_8659
solcap_snp_c1_8679
solcap_snp_c2_50799 solcap_snp_c2_50798
solcap_snp_c2_50796 solcap_snp_c2_50795
solcap_snp_c2_41223 solcap_snp_c2_41210
solcap_snp_c1_3074 solcap_snp_c2_9047
solcap_snp_c2_9038
solcap_snp_c1_3125 solcap_snp_c2_9292
solcap_snp_c2_9220 solcap_snp_c2_9219
solcap_snp_c2_9203 solcap_snp_c2_9201
solcap_snp_c2_9137
solcap_snp_c2_9233 solcap_snp_c2_9172
solcap_snp_c1_13951

chr06_Premier_Russet

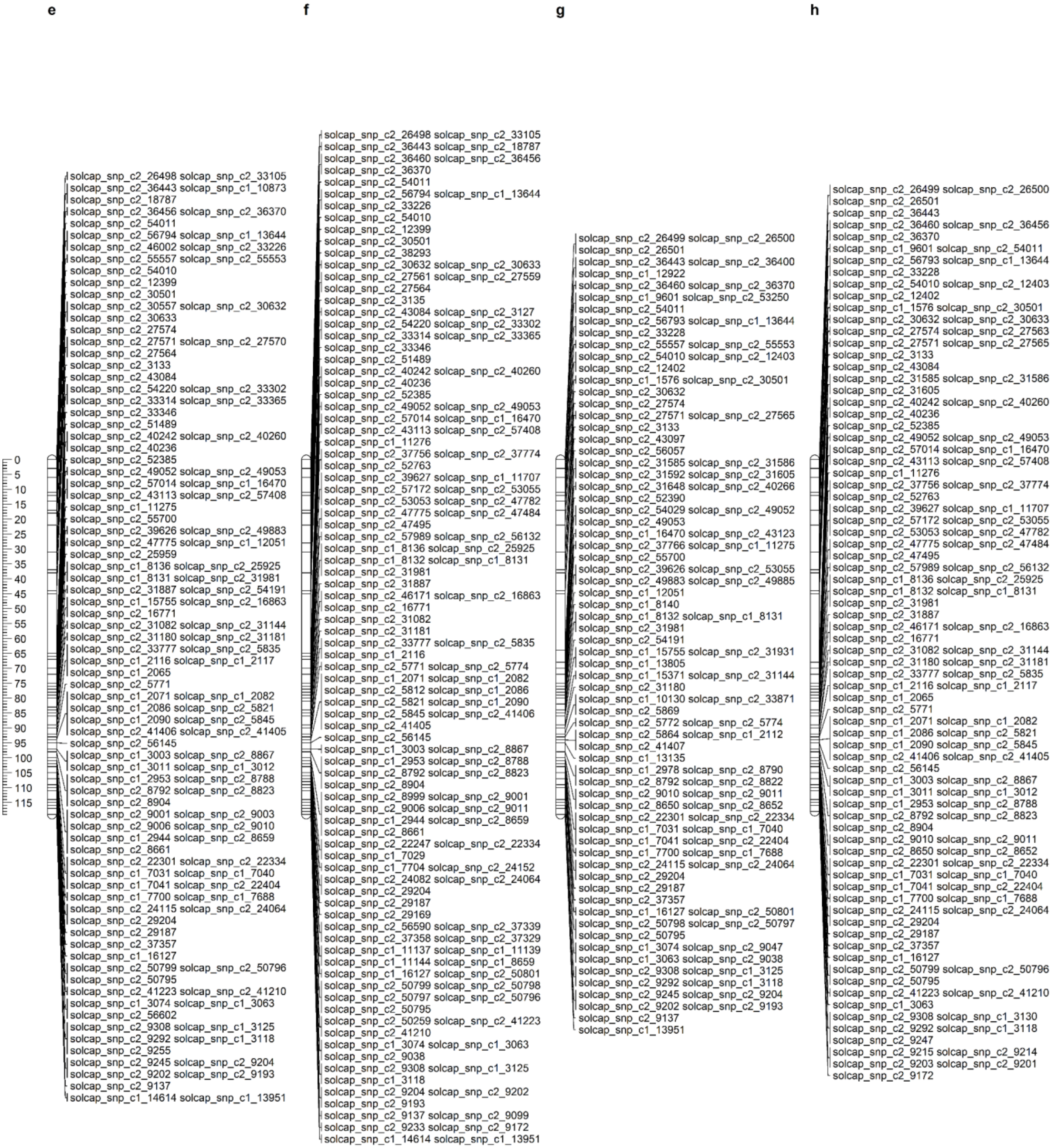

chr07_Rio_Grande_Russet

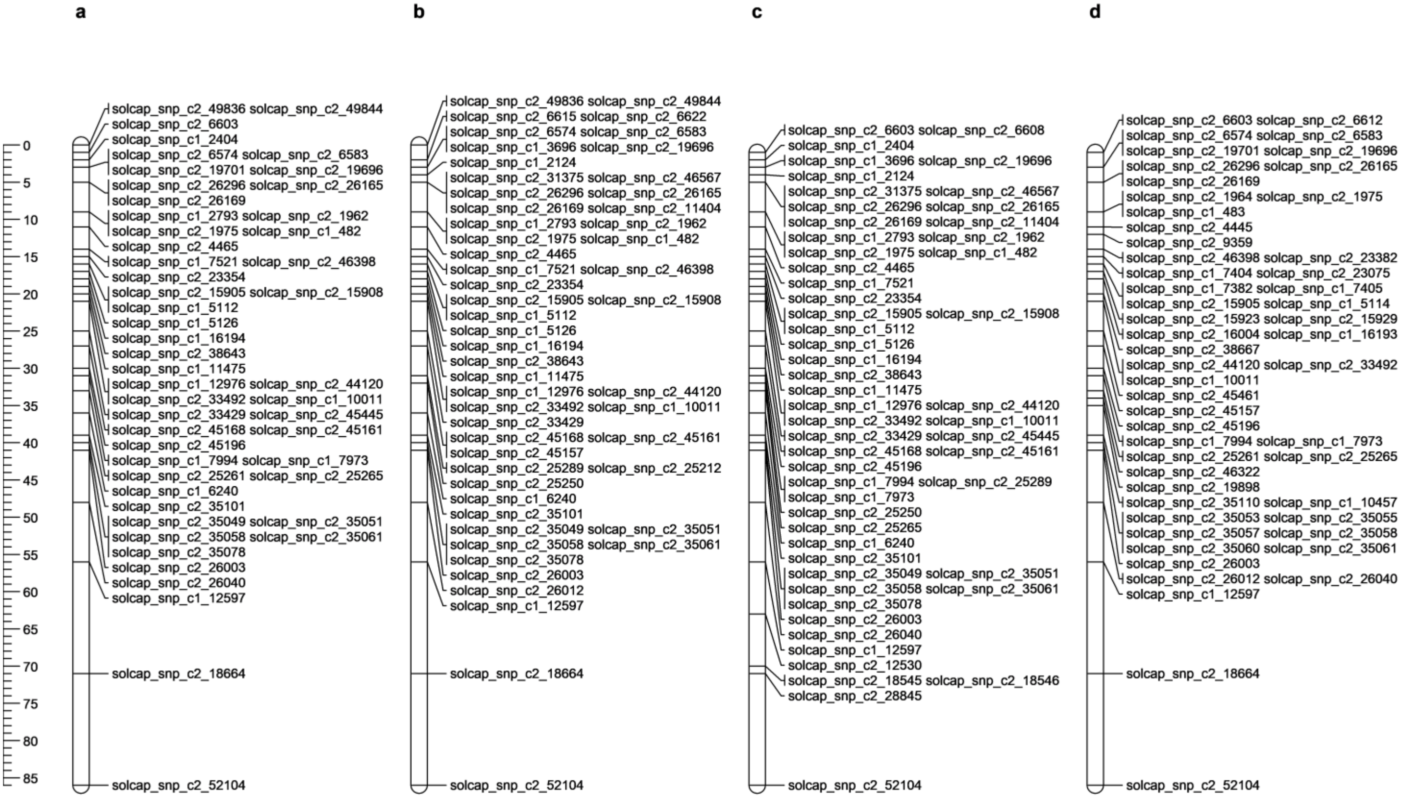

chr07_Premier_Russet

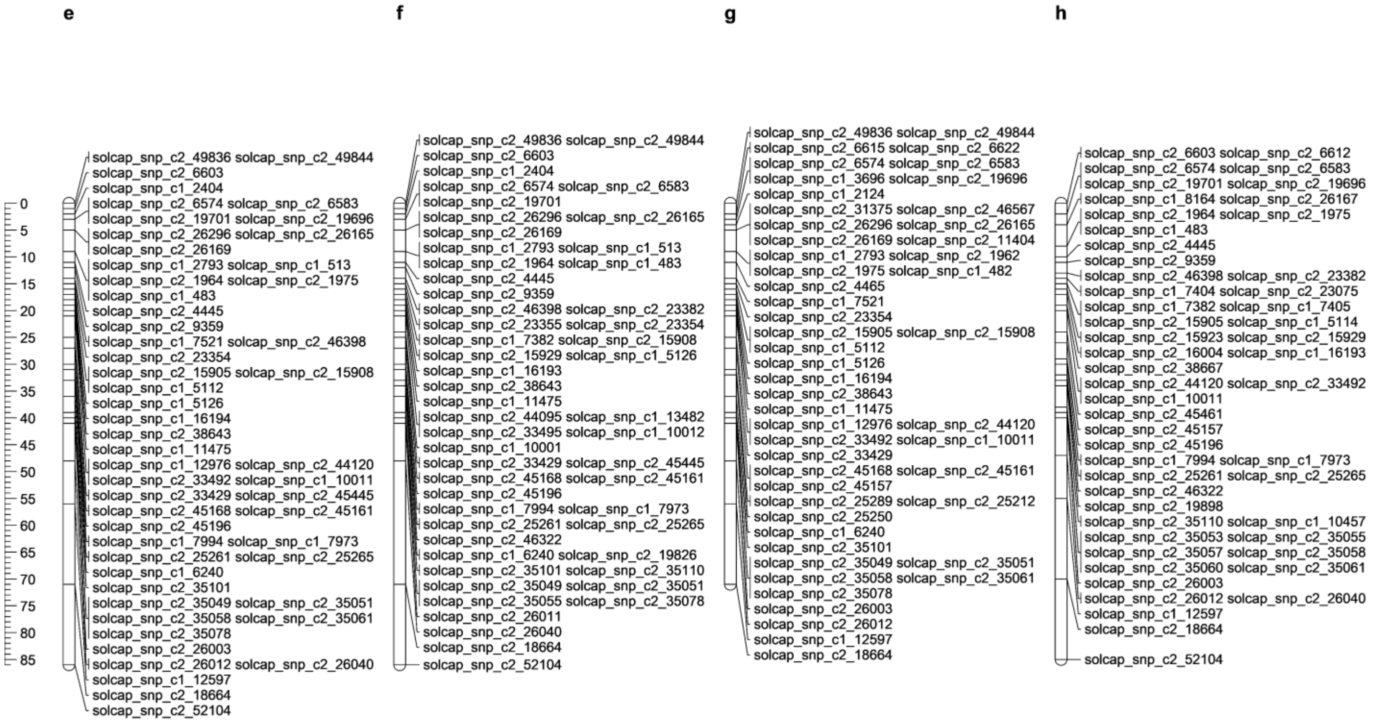

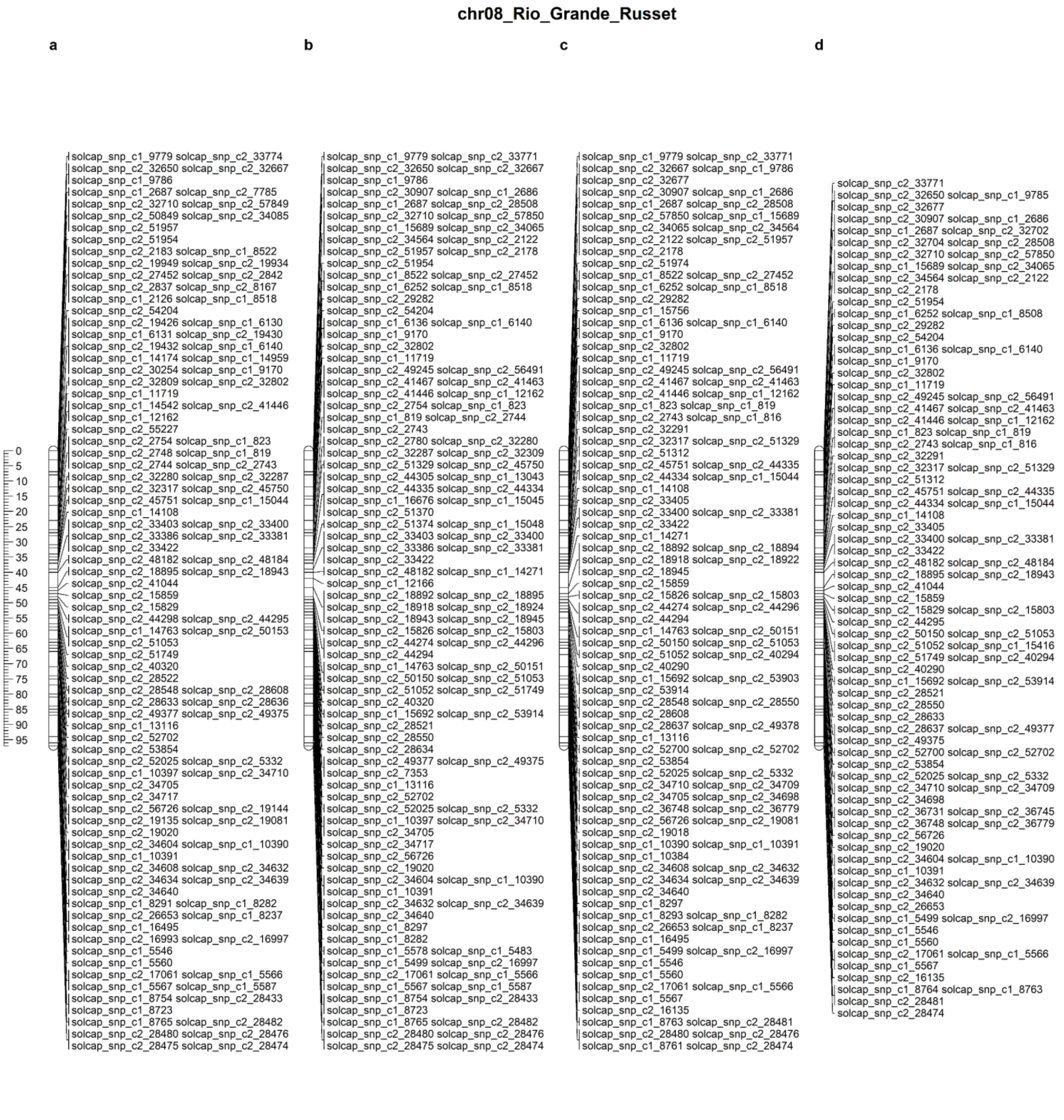
chr08_Rio_Grande_Russet
a
b
c
d
0
5
10
15
20
25
30
35
40
45
50
55
60
65
70
75
80
85
90
95
solcap_snp_c1_9779 solcap_snp_c2_33774
solcap_snp_c2_32650 solcap_snp_c2_32667
solcap_snp_c1_9786
solcap_snp_c1_2687 solcap_snp_c2_7785
solcap_snp_c2_32710 solcap_snp_c2_57849
solcap_snp_c2_50849 solcap_snp_c2_34085
solcap_snp_c2_51957
solcap_snp_c2_51954
solcap_snp_c2_2183 solcap_snp_c1_8522
solcap_snp_c2_19949 solcap_snp_c2_19934
solcap_snp_c2_27452 solcap_snp_c2_2842
solcap_snp_c2_2837 solcap_snp_c2_8167
solcap_snp_c1_2126 solcap_snp_c1_8518
solcap_snp_c2_54204
solcap_snp_c2_19426 solcap_snp_c1_6130
solcap_snp_c1_6131 solcap_snp_c2_19430
solcap_snp_c2_19432 solcap_snp_c1_6140
solcap_snp_c1_14174 solcap_snp_c1_14959
solcap_snp_c2_30254 solcap_snp_c1_9170
solcap_snp_c2_32809 solcap_snp_c2_32802
solcap_snp_c1_11719
solcap_snp_c1_14542 solcap_snp_c2_41446
solcap_snp_c1_12162
solcap_snp_c2_55227
solcap_snp_c2_2754 solcap_snp_c1_823
solcap_snp_c2_2748 solcap_snp_c1_819
solcap_snp_c2_2744 solcap_snp_c2_2743
solcap_snp_c2_32280 solcap_snp_c2_32287
solcap_snp_c2_32317 solcap_snp_c2_45750
solcap_snp_c2_45751 solcap_snp_c1_15044
solcap_snp_c1_14108
solcap_snp_c2_33403 solcap_snp_c2_33400
solcap_snp_c2_33386 solcap_snp_c2_33381
solcap_snp_c2_33422
solcap_snp_c2_48182 solcap_snp_c2_48184
solcap_snp_c2_18895 solcap_snp_c2_18943
solcap_snp_c2_41044
solcap_snp_c2_15859
solcap_snp_c2_15829
solcap_snp_c2_44298 solcap_snp_c2_44295
solcap_snp_c1_14763 solcap_snp_c2_50153
solcap_snp_c2_51053
solcap_snp_c2_51749
solcap_snp_c2_40320
solcap_snp_c2_28522
solcap_snp_c2_28548 solcap_snp_c2_28608
solcap_snp_c2_28633 solcap_snp_c2_28636
solcap_snp_c2_49377 solcap_snp_c2_49375
solcap_snp_c1_13116
solcap_snp_c2_52702
solcap_snp_c2_53854
solcap_snp_c2_52025 solcap_snp_c2_5332
solcap_snp_c1_10397 solcap_snp_c2_34710
solcap_snp_c2_34705
solcap_snp_c2_34717
solcap_snp_c2_56726 solcap_snp_c2_19144
solcap_snp_c2_19135 solcap_snp_c2_19081
solcap_snp_c2_19020
solcap_snp_c2_34604 solcap_snp_c1_10390
solcap_snp_c1_10391
solcap_snp_c2_34608 solcap_snp_c2_34632
solcap_snp_c2_34634 solcap_snp_c2_34639
solcap_snp_c2_34640
solcap_snp_c1_8291 solcap_snp_c1_8282
solcap_snp_c2_26653 solcap_snp_c1_8237
solcap_snp_c1_16495
solcap_snp_c2_16993 solcap_snp_c2_16997
solcap_snp_c1_5546
solcap_snp_c1_5560
solcap_snp_c2_17061 solcap_snp_c1_5566
solcap_snp_c1_5567 solcap_snp_c1_5587
solcap_snp_c1_8754 solcap_snp_c2_28433
solcap_snp_c1_8723
solcap_snp_c1_8765 solcap_snp_c2_28482
solcap_snp_c2_28480 solcap_snp_c2_28476
solcap_snp_c2_28475 solcap_snp_c2_28474
solcap_snp_c1_9779 solcap_snp_c2_33771
solcap_snp_c2_32650 solcap_snp_c2_32667
solcap_snp_c1_9786
solcap_snp_c2_30907 solcap_snp_c1_2686
solcap_snp_c1_2687 solcap_snp_c2_28508
solcap_snp_c2_32710 solcap_snp_c2_57850
solcap_snp_c1_15689 solcap_snp_c2_34065
solcap_snp_c2_34564 solcap_snp_c2_2122
solcap_snp_c2_51957 solcap_snp_c2_2178
solcap_snp_c2_51954
solcap_snp_c1_8522 solcap_snp_c2_27452
solcap_snp_c1_6252 solcap_snp_c1_8518
solcap_snp_c2_29282
solcap_snp_c2_54204
solcap_snp_c1_6136 solcap_snp_c1_6140
solcap_snp_c1_9170
solcap_snp_c2_32802
solcap_snp_c1_11719
solcap_snp_c2_49245 solcap_snp_c2_56491
solcap_snp_c2_41467 solcap_snp_c2_41463
solcap_snp_c2_41446 solcap_snp_c1_12162
solcap_snp_c2_2754 solcap_snp_c1_823
solcap_snp_c1_819 solcap_snp_c2_2744
solcap_snp_c2_2743
solcap_snp_c2_2780 solcap_snp_c2_32280
solcap_snp_c2_32287 solcap_snp_c2_32309
solcap_snp_c2_51329 solcap_snp_c2_45750
solcap_snp_c2_44305 solcap_snp_c1_13043
solcap_snp_c2_44335 solcap_snp_c2_44334
solcap_snp_c1_16676 solcap_snp_c1_15045
solcap_snp_c2_51370
solcap_snp_c2_51374 solcap_snp_c1_15048
solcap_snp_c2_33403 solcap_snp_c2_33400
solcap_snp_c2_33386 solcap_snp_c2_33381
solcap_snp_c2_33422
solcap_snp_c2_48182 solcap_snp_c1_14271
solcap_snp_c1_12166
solcap_snp_c2_18892 solcap_snp_c2_18895
solcap_snp_c2_18918 solcap_snp_c2_18924
solcap_snp_c2_18943 solcap_snp_c2_18945
solcap_snp_c2_15826 solcap_snp_c2_15803
solcap_snp_c2_44274 solcap_snp_c2_44296
solcap_snp_c2_44294
solcap_snp_c1_14763 solcap_snp_c2_50151
solcap_snp_c2_50150 solcap_snp_c2_51053
solcap_snp_c2_51052 solcap_snp_c2_51749
solcap_snp_c2_40320
solcap_snp_c1_15692 solcap_snp_c2_53914
solcap_snp_c2_28521
solcap_snp_c2_28550
solcap_snp_c2_28634
solcap_snp_c2_49377 solcap_snp_c2_49375
solcap_snp_c2_7353
solcap_snp_c1_13116
solcap_snp_c2_52702
solcap_snp_c2_52025 solcap_snp_c2_5332
solcap_snp_c1_10397 solcap_snp_c2_34710
solcap_snp_c2_34705
solcap_snp_c2_34717
solcap_snp_c2_56726
solcap_snp_c2_19020
solcap_snp_c2_34604 solcap_snp_c1_10390
solcap_snp_c1_10391
solcap_snp_c2_34632 solcap_snp_c2_34639
solcap_snp_c2_34640
solcap_snp_c1_8297
solcap_snp_c1_8282
solcap_snp_c1_5578 solcap_snp_c1_5483
solcap_snp_c1_5499 solcap_snp_c2_16997
solcap_snp_c2_17061 solcap_snp_c1_5566
solcap_snp_c1_5567 solcap_snp_c1_5587
solcap_snp_c1_8754 solcap_snp_c2_28433
solcap_snp_c1_8723
solcap_snp_c1_8765 solcap_snp_c2_28482
solcap_snp_c2_28480 solcap_snp_c2_28476
solcap_snp_c2_28475 solcap_snp_c2_28474
solcap_snp_c1_9779 solcap_snp_c2_33771
solcap_snp_c2_32667 solcap_snp_c1_9786
solcap_snp_c2_32677
solcap_snp_c2_30907 solcap_snp_c1_2686
solcap_snp_c1_2687 solcap_snp_c2_28508
solcap_snp_c2_57850 solcap_snp_c1_15689
solcap_snp_c2_34065 solcap_snp_c2_34564
solcap_snp_c2_2122 solcap_snp_c2_51957
solcap_snp_c2_2178
solcap_snp_c2_51974
solcap_snp_c1_8522 solcap_snp_c2_27452
solcap_snp_c1_6252 solcap_snp_c1_8518
solcap_snp_c2_29282
solcap_snp_c1_15756
solcap_snp_c1_6136 solcap_snp_c1_6140
solcap_snp_c1_9170
solcap_snp_c2_32802
solcap_snp_c1_11719
solcap_snp_c2_49245 solcap_snp_c2_56491
solcap_snp_c2_41467 solcap_snp_c2_41463
solcap_snp_c2_41446 solcap_snp_c1_12162
solcap_snp_c1_823 solcap_snp_c1_819
solcap_snp_c2_2743 solcap_snp_c1_816
solcap_snp_c2_32291
solcap_snp_c2_32317 solcap_snp_c2_51329
solcap_snp_c2_51312
solcap_snp_c2_45751 solcap_snp_c2_44335
solcap_snp_c2_44334 solcap_snp_c1_15044
solcap_snp_c1_14108
solcap_snp_c2_33405
solcap_snp_c2_33400 solcap_snp_c2_33381
solcap_snp_c2_33422
solcap_snp_c1_14271
solcap_snp_c2_18892 solcap_snp_c2_18894
solcap_snp_c2_18918 solcap_snp_c2_18922
solcap_snp_c2_18945
solcap_snp_c2_15859
solcap_snp_c2_15826 solcap_snp_c2_15803
solcap_snp_c2_44274 solcap_snp_c2_44296
solcap_snp_c2_44294
solcap_snp_c1_14763 solcap_snp_c2_50151
solcap_snp_c2_50150 solcap_snp_c2_51053
solcap_snp_c2_51052 solcap_snp_c2_40294
solcap_snp_c2_40290
solcap_snp_c1_15692 solcap_snp_c2_53903
solcap_snp_c2_53914
solcap_snp_c2_28548 solcap_snp_c2_28550
solcap_snp_c2_28608
solcap_snp_c2_28637 solcap_snp_c2_49378
solcap_snp_c1_13116
solcap_snp_c2_52700 solcap_snp_c2_52702
solcap_snp_c2_53854
solcap_snp_c2_52025 solcap_snp_c2_5332
solcap_snp_c2_34710 solcap_snp_c2_34709
solcap_snp_c2_34705 solcap_snp_c2_34698
solcap_snp_c2_36748 solcap_snp_c2_36779
solcap_snp_c2_56726 solcap_snp_c2_19081
solcap_snp_c2_19018
solcap_snp_c1_10390 solcap_snp_c1_10391
solcap_snp_c1_10384
solcap_snp_c2_34608 solcap_snp_c2_34632
solcap_snp_c2_34634 solcap_snp_c2_34639
solcap_snp_c2_34640
solcap_snp_c1_8297
solcap_snp_c1_8293 solcap_snp_c1_8282
solcap_snp_c2_26653 solcap_snp_c1_8237
solcap_snp_c1_16495
solcap_snp_c1_5499 solcap_snp_c2_16997
solcap_snp_c1_5546
solcap_snp_c1_5560
solcap_snp_c2_17061 solcap_snp_c1_5566
solcap_snp_c1_5567
solcap_snp_c2_16135
solcap_snp_c1_8763 solcap_snp_c2_28481
solcap_snp_c2_28480 solcap_snp_c2_28476
solcap_snp_c1_8761 solcap_snp_c2_28474
solcap_snp_c2_33771
solcap_snp_c2_32650 solcap_snp_c1_9785
solcap_snp_c2_32677
solcap_snp_c2_30907 solcap_snp_c1_2686
solcap_snp_c1_2687 solcap_snp_c2_32702
solcap_snp_c2_32704 solcap_snp_c2_28508
solcap_snp_c2_32710 solcap_snp_c2_57850
solcap_snp_c1_15689 solcap_snp_c2_34065
solcap_snp_c2_34564 solcap_snp_c2_2122
solcap_snp_c2_2178
solcap_snp_c2_51954
solcap_snp_c1_6252 solcap_snp_c1_8508
solcap_snp_c2_29282
solcap_snp_c2_54204
solcap_snp_c1_6136 solcap_snp_c1_6140
solcap_snp_c1_9170
solcap_snp_c2_32802
solcap_snp_c1_11719
solcap_snp_c2_49245 solcap_snp_c2_56491
solcap_snp_c2_41467 solcap_snp_c2_41463
solcap_snp_c2_41446 solcap_snp_c1_12162
solcap_snp_c1_823 solcap_snp_c1_819
solcap_snp_c2_2743 solcap_snp_c1_816
solcap_snp_c2_32291
solcap_snp_c2_32317 solcap_snp_c2_51329
solcap_snp_c2_51312
solcap_snp_c2_45751 solcap_snp_c2_44335
solcap_snp_c2_44334 solcap_snp_c1_15044
solcap_snp_c1_14108
solcap_snp_c2_33405
solcap_snp_c2_33400 solcap_snp_c2_33381
solcap_snp_c2_33422
solcap_snp_c2_48182 solcap_snp_c2_48184
solcap_snp_c2_18895 solcap_snp_c2_18943
solcap_snp_c2_41044
solcap_snp_c2_15859
solcap_snp_c2_15829 solcap_snp_c2_15803
solcap_snp_c2_44295
solcap_snp_c2_50150 solcap_snp_c2_51053
solcap_snp_c2_51052 solcap_snp_c1_15416
solcap_snp_c2_51749 solcap_snp_c2_40294
solcap_snp_c2_40290
solcap_snp_c1_15692 solcap_snp_c2_53914
solcap_snp_c2_28521
solcap_snp_c2_28550
solcap_snp_c2_28633
solcap_snp_c2_28637 solcap_snp_c2_49377
solcap_snp_c2_49375
solcap_snp_c2_52700 solcap_snp_c2_52702
solcap_snp_c2_53854
solcap_snp_c2_52025 solcap_snp_c2_5332
solcap_snp_c2_34710 solcap_snp_c2_34709
solcap_snp_c2_34698
solcap_snp_c2_36731 solcap_snp_c2_36745
solcap_snp_c2_36748 solcap_snp_c2_36779
solcap_snp_c2_56726
solcap_snp_c2_19020
solcap_snp_c2_34604 solcap_snp_c1_10390
solcap_snp_c1_10391
solcap_snp_c2_34632 solcap_snp_c2_34639
solcap_snp_c2_34640
solcap_snp_c2_26653
solcap_snp_c1_5499 solcap_snp_c2_16997
solcap_snp_c1_5546
solcap_snp_c1_5560
solcap_snp_c2_17061 solcap_snp_c1_5566
solcap_snp_c1_5567
solcap_snp_c2_16135
solcap_snp_c1_8764 solcap_snp_c1_8763
solcap_snp_c2_28481
solcap_snp_c2_28474

chr08_Premier_Russet

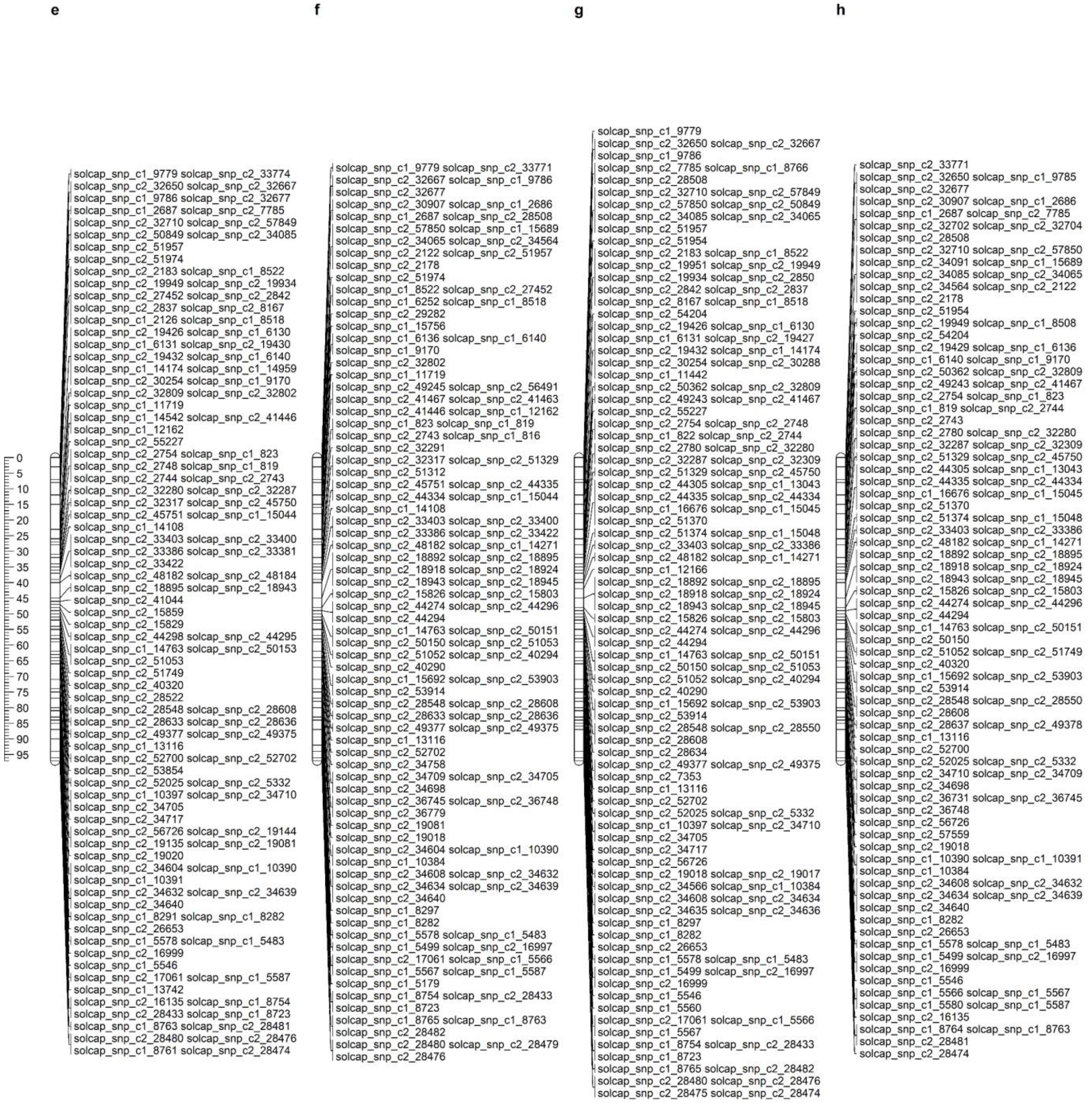

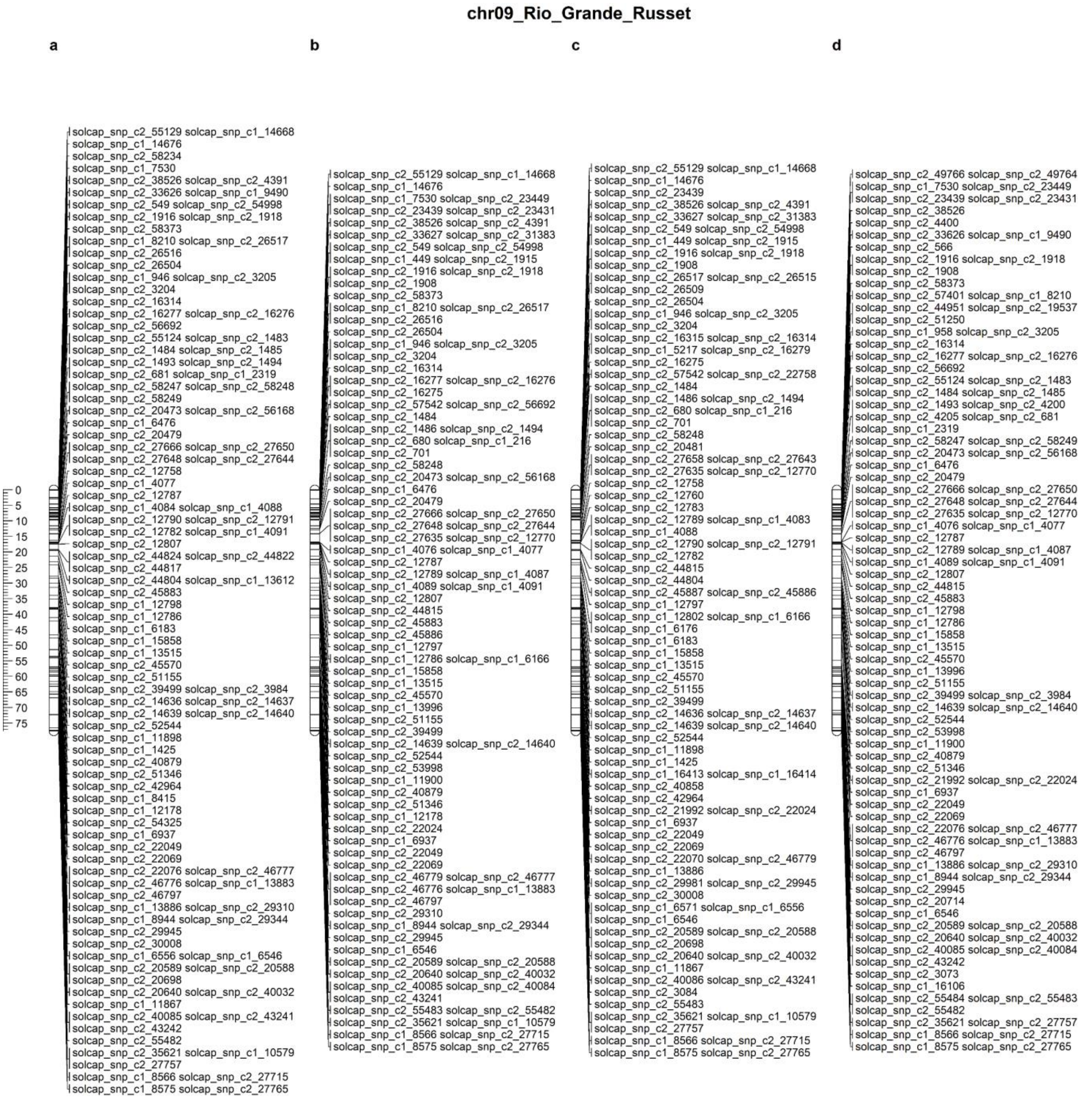
chr09_Rio_Grande_Russet
a
b
c
d
0
5
10
15
20
25
30
35
40
45
50
55
60
65
70
75
solcap_snp_c2_55129 solcap_snp_c1_14668
solcap_snp_c1_14676
solcap_snp_c2_58234
solcap_snp_c1_7530
solcap_snp_c2_38526 solcap_snp_c2_4391
solcap_snp_c2_33626 solcap_snp_c1_9490
solcap_snp_c2_549 solcap_snp_c2_54998
solcap_snp_c2_1916 solcap_snp_c2_1918
solcap_snp_c2_58373
solcap_snp_c1_8210 solcap_snp_c2_26517
solcap_snp_c2_26516
solcap_snp_c2_26504
solcap_snp_c1_946 solcap_snp_c2_3205
solcap_snp_c2_3204
solcap_snp_c2_16314
solcap_snp_c2_16277 solcap_snp_c2_16276
solcap_snp_c2_56692
solcap_snp_c2_55124 solcap_snp_c2_1483
solcap_snp_c2_1484 solcap_snp_c2_1485
solcap_snp_c2_1493 solcap_snp_c2_1494
solcap_snp_c2_681 solcap_snp_c1_2319
solcap_snp_c2_58247 solcap_snp_c2_58248
solcap_snp_c2_58249
solcap_snp_c2_20473 solcap_snp_c2_56168
solcap_snp_c1_6476
solcap_snp_c2_20479
solcap_snp_c2_27666 solcap_snp_c2_27650
solcap_snp_c2_27648 solcap_snp_c2_27644
solcap_snp_c2_12758
solcap_snp_c1_4077
solcap_snp_c2_12787
solcap_snp_c1_4084 solcap_snp_c1_4088
solcap_snp_c2_12790 solcap_snp_c2_12791
solcap_snp_c2_12782 solcap_snp_c1_4091
solcap_snp_c2_12807
solcap_snp_c2_44824 solcap_snp_c2_44822
solcap_snp_c2_44817
solcap_snp_c2_44804 solcap_snp_c1_13612
solcap_snp_c2_45883
solcap_snp_c1_12798
solcap_snp_c1_12786
solcap_snp_c1_6183
solcap_snp_c1_15858
solcap_snp_c1_13515
solcap_snp_c2_45570
solcap_snp_c2_51155
solcap_snp_c2_39499 solcap_snp_c2_3984
solcap_snp_c2_14636 solcap_snp_c2_14637
solcap_snp_c2_14639 solcap_snp_c2_14640
solcap_snp_c2_52544
solcap_snp_c1_11898
solcap_snp_c1_1425
solcap_snp_c2_40879
solcap_snp_c2_51346
solcap_snp_c2_42964
solcap_snp_c1_8415
solcap_snp_c1_12178
solcap_snp_c2_54325
solcap_snp_c1_6937
solcap_snp_c2_22049
solcap_snp_c2_22069
solcap_snp_c2_22076 solcap_snp_c2_46777
solcap_snp_c2_46776 solcap_snp_c1_13883
solcap_snp_c2_46797
solcap_snp_c1_13886 solcap_snp_c2_29310
solcap_snp_c1_8944 solcap_snp_c2_29344
solcap_snp_c2_29945
solcap_snp_c2_30008
solcap_snp_c1_6556 solcap_snp_c1_6546
solcap_snp_c2_20589 solcap_snp_c2_20588
solcap_snp_c2_20698
solcap_snp_c2_20640 solcap_snp_c2_40032
solcap_snp_c1_11867
solcap_snp_c2_40085 solcap_snp_c2_43241
solcap_snp_c2_43242
solcap_snp_c2_55482
solcap_snp_c2_35621 solcap_snp_c1_10579
solcap_snp_c2_27757
solcap_snp_c1_8566 solcap_snp_c2_27715
solcap_snp_c1_8575 solcap_snp_c2_27765
solcap_snp_c2_55129 solcap_snp_c1_14668
solcap_snp_c1_14676
solcap_snp_c1_7530 solcap_snp_c2_23449
solcap_snp_c2_23439 solcap_snp_c2_23431
solcap_snp_c2_38526 solcap_snp_c2_4391
solcap_snp_c2_33627 solcap_snp_c2_31383
solcap_snp_c2_549 solcap_snp_c2_54998
solcap_snp_c1_449 solcap_snp_c2_1915
solcap_snp_c2_1916 solcap_snp_c2_1918
solcap_snp_c2_1908
solcap_snp_c2_58373
solcap_snp_c1_8210 solcap_snp_c2_26517
solcap_snp_c2_26516
solcap_snp_c2_26504
solcap_snp_c1_946 solcap_snp_c2_3205
solcap_snp_c2_3204
solcap_snp_c2_16314
solcap_snp_c2_16277 solcap_snp_c2_16276
solcap_snp_c2_16275
solcap_snp_c2_57542 solcap_snp_c2_56692
solcap_snp_c2_1484
solcap_snp_c2_1486 solcap_snp_c2_1494
solcap_snp_c2_680 solcap_snp_c1_216
solcap_snp_c2_701
solcap_snp_c2_58248
solcap_snp_c2_20473 solcap_snp_c2_56168
solcap_snp_c1_6476
solcap_snp_c2_20479
solcap_snp_c2_27666 solcap_snp_c2_27650
solcap_snp_c2_27648 solcap_snp_c2_27644
solcap_snp_c2_27635 solcap_snp_c2_12770
solcap_snp_c1_4076 solcap_snp_c1_4077
solcap_snp_c2_12787
solcap_snp_c2_12789 solcap_snp_c1_4087
solcap_snp_c1_4089 solcap_snp_c1_4091
solcap_snp_c2_12807
solcap_snp_c2_44815
solcap_snp_c2_45883
solcap_snp_c2_45886
solcap_snp_c1_12797
solcap_snp_c1_12786 solcap_snp_c1_6166
solcap_snp_c1_15858
solcap_snp_c1_13515
solcap_snp_c2_45570
solcap_snp_c1_13996
solcap_snp_c2_51155
solcap_snp_c2_39499
solcap_snp_c2_14639 solcap_snp_c2_14640
solcap_snp_c2_52544
solcap_snp_c2_53998
solcap_snp_c1_11900
solcap_snp_c2_40879
solcap_snp_c2_51346
solcap_snp_c1_12178
solcap_snp_c2_22024
solcap_snp_c1_6937
solcap_snp_c2_22049
solcap_snp_c2_22069
solcap_snp_c2_46779 solcap_snp_c2_46777
solcap_snp_c2_46776 solcap_snp_c1_13883
solcap_snp_c2_46797
solcap_snp_c2_29310
solcap_snp_c1_8944 solcap_snp_c2_29344
solcap_snp_c2_29945
solcap_snp_c1_6546
solcap_snp_c2_20589 solcap_snp_c2_20588
solcap_snp_c2_20640 solcap_snp_c2_40032
solcap_snp_c2_40085 solcap_snp_c2_40084
solcap_snp_c2_43241
solcap_snp_c2_55483 solcap_snp_c2_55482
solcap_snp_c2_35621 solcap_snp_c1_10579
solcap_snp_c1_8566 solcap_snp_c2_27715
solcap_snp_c1_8575 solcap_snp_c2_27765
solcap_snp_c2_55129 solcap_snp_c1_14668
solcap_snp_c1_14676
solcap_snp_c2_23439
solcap_snp_c2_38526 solcap_snp_c2_4391
solcap_snp_c2_33627 solcap_snp_c2_31383
solcap_snp_c2_549 solcap_snp_c2_54998
solcap_snp_c1_449 solcap_snp_c2_1915
solcap_snp_c2_1916 solcap_snp_c2_1918
solcap_snp_c2_1908
solcap_snp_c2_26517 solcap_snp_c2_26515
solcap_snp_c2_26509
solcap_snp_c2_26504
solcap_snp_c1_946 solcap_snp_c2_3205
solcap_snp_c2_3204
solcap_snp_c2_16315 solcap_snp_c2_16314
solcap_snp_c1_5217 solcap_snp_c2_16279
solcap_snp_c2_16275
solcap_snp_c2_57542 solcap_snp_c2_22758
solcap_snp_c2_1484
solcap_snp_c2_1486 solcap_snp_c2_1494
solcap_snp_c2_680 solcap_snp_c1_216
solcap_snp_c2_701
solcap_snp_c2_58248
solcap_snp_c2_20481
solcap_snp_c2_27658 solcap_snp_c2_27643
solcap_snp_c2_27635 solcap_snp_c2_12770
solcap_snp_c2_12758
solcap_snp_c2_12760
solcap_snp_c2_12783
solcap_snp_c2_12789 solcap_snp_c1_4083
solcap_snp_c1_4088
solcap_snp_c2_12790 solcap_snp_c2_12791
solcap_snp_c2_12782
solcap_snp_c2_44815
solcap_snp_c2_44804
solcap_snp_c2_45887 solcap_snp_c2_45886
solcap_snp_c1_12797
solcap_snp_c1_12802 solcap_snp_c1_6166
solcap_snp_c1_6176
solcap_snp_c1_6183
solcap_snp_c1_15858
solcap_snp_c1_13515
solcap_snp_c2_45570
solcap_snp_c2_51155
solcap_snp_c2_39499
solcap_snp_c2_14636 solcap_snp_c2_14637
solcap_snp_c2_14639 solcap_snp_c2_14640
solcap_snp_c2_52544
solcap_snp_c1_11898
solcap_snp_c1_1425
solcap_snp_c1_16413 solcap_snp_c1_16414
solcap_snp_c2_40858
solcap_snp_c2_42964
solcap_snp_c2_21992 solcap_snp_c2_22024
solcap_snp_c1_6937
solcap_snp_c2_22049
solcap_snp_c2_22069
solcap_snp_c2_22070 solcap_snp_c2_46779
solcap_snp_c1_13886
solcap_snp_c2_29981 solcap_snp_c2_29945
solcap_snp_c2_30008
solcap_snp_c1_6571 solcap_snp_c1_6556
solcap_snp_c1_6546
solcap_snp_c2_20589 solcap_snp_c2_20588
solcap_snp_c2_20698
solcap_snp_c2_20640 solcap_snp_c2_40032
solcap_snp_c1_11867
solcap_snp_c2_40086 solcap_snp_c2_43241
solcap_snp_c2_3084
solcap_snp_c2_55483
solcap_snp_c2_35621 solcap_snp_c1_10579
solcap_snp_c2_27757
solcap_snp_c1_8566 solcap_snp_c2_27715
solcap_snp_c1_8575 solcap_snp_c2_27765
solcap_snp_c2_49766 solcap_snp_c2_49764
solcap_snp_c1_7530 solcap_snp_c2_23449
solcap_snp_c2_23439 solcap_snp_c2_23431
solcap_snp_c2_38526
solcap_snp_c2_4400
solcap_snp_c2_33626 solcap_snp_c1_9490
solcap_snp_c2_566
solcap_snp_c2_1916 solcap_snp_c2_1918
solcap_snp_c2_1908
solcap_snp_c2_58373
solcap_snp_c2_57401 solcap_snp_c1_8210
solcap_snp_c2_44951 solcap_snp_c2_19537
solcap_snp_c2_51250
solcap_snp_c1_958 solcap_snp_c2_3205
solcap_snp_c2_16314
solcap_snp_c2_16277 solcap_snp_c2_16276
solcap_snp_c2_56692
solcap_snp_c2_55124 solcap_snp_c2_1483
solcap_snp_c2_1484 solcap_snp_c2_1485
solcap_snp_c2_1493 solcap_snp_c2_4200
solcap_snp_c2_4205 solcap_snp_c2_681
solcap_snp_c1_2319
solcap_snp_c2_58247 solcap_snp_c2_58249
solcap_snp_c2_20473 solcap_snp_c2_56168
solcap_snp_c1_6476
solcap_snp_c2_20479
solcap_snp_c2_27666 solcap_snp_c2_27650
solcap_snp_c2_27648 solcap_snp_c2_27644
solcap_snp_c2_27635 solcap_snp_c2_12770
solcap_snp_c1_4076 solcap_snp_c1_4077
solcap_snp_c2_12787
solcap_snp_c2_12789 solcap_snp_c1_4087
solcap_snp_c1_4089 solcap_snp_c1_4091
solcap_snp_c2_12807
solcap_snp_c2_44815
solcap_snp_c2_45883
solcap_snp_c1_12798
solcap_snp_c1_12786
solcap_snp_c1_15858
solcap_snp_c1_13515
solcap_snp_c2_45570
solcap_snp_c1_13996
solcap_snp_c2_51155
solcap_snp_c2_39499 solcap_snp_c2_3984
solcap_snp_c2_14639 solcap_snp_c2_14640
solcap_snp_c2_52544
solcap_snp_c2_53998
solcap_snp_c1_11900
solcap_snp_c2_40879
solcap_snp_c2_51346
solcap_snp_c2_21992 solcap_snp_c2_22024
solcap_snp_c1_6937
solcap_snp_c2_22049
solcap_snp_c2_22069
solcap_snp_c2_22076 solcap_snp_c2_46777
solcap_snp_c2_46776 solcap_snp_c1_13883
solcap_snp_c2_46797
solcap_snp_c1_13886 solcap_snp_c2_29310
solcap_snp_c1_8944 solcap_snp_c2_29344
solcap_snp_c2_29945
solcap_snp_c2_20714
solcap_snp_c1_6546
solcap_snp_c2_20589 solcap_snp_c2_20588
solcap_snp_c2_20640 solcap_snp_c2_40032
solcap_snp_c2_40085 solcap_snp_c2_40084
solcap_snp_c2_43242
solcap_snp_c2_3073
solcap_snp_c1_16106
solcap_snp_c2_55484 solcap_snp_c2_55483
solcap_snp_c2_55482
solcap_snp_c2_35621 solcap_snp_c2_27757
solcap_snp_c1_8566 solcap_snp_c2_27715
solcap_snp_c1_8575 solcap_snp_c2_27765

chr09_Premier_Russet

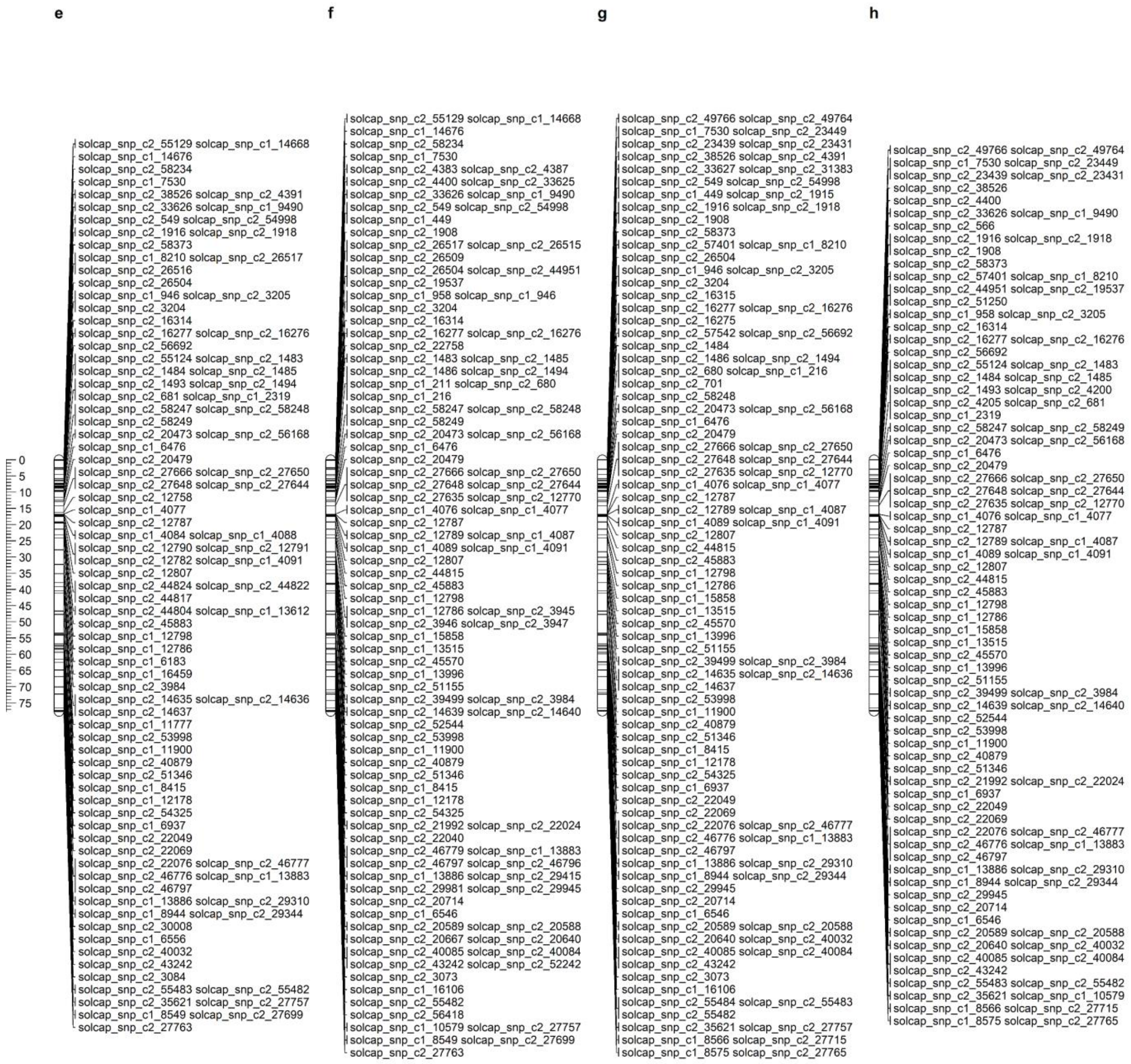

chr10_Rio_Grande_Russet

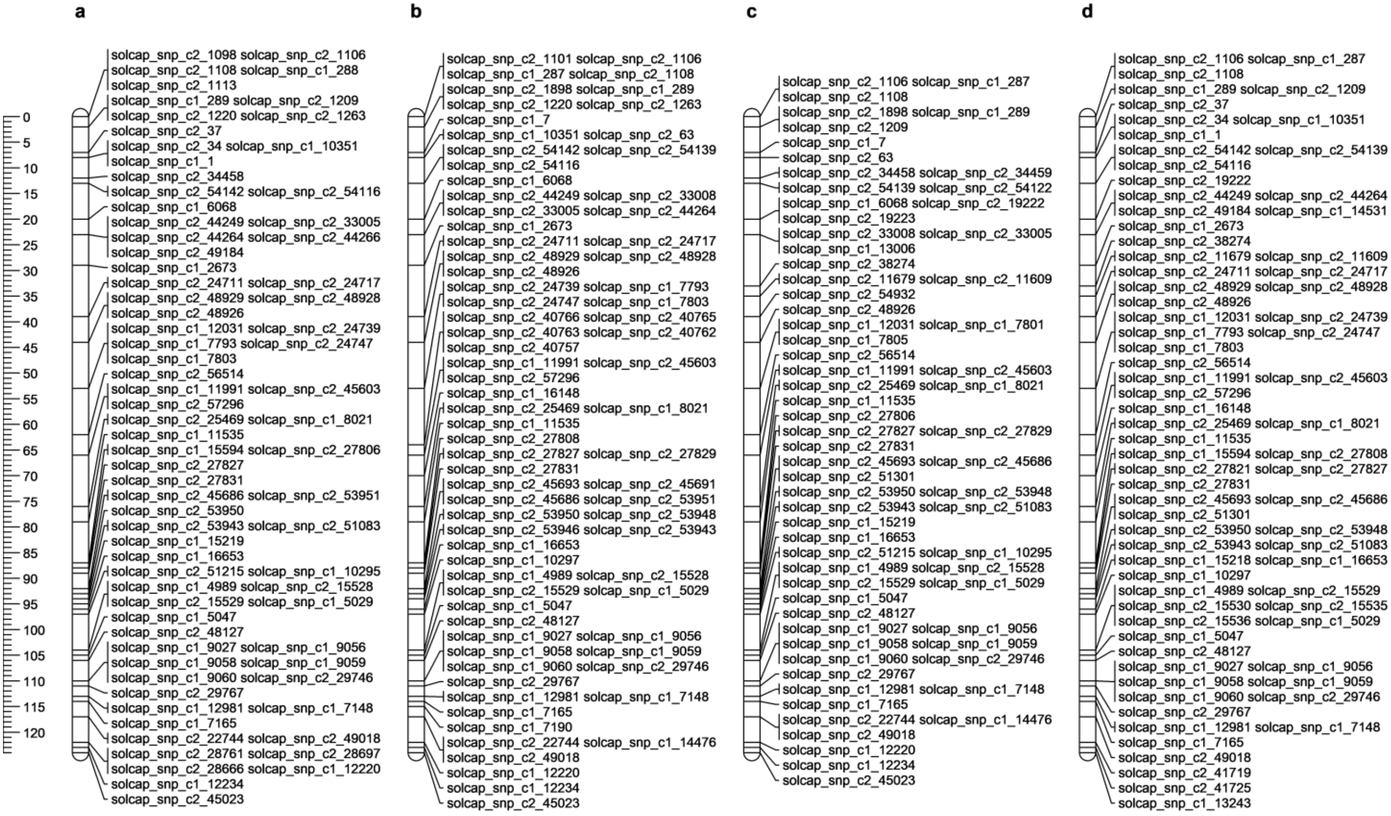

chr10_Premier_Russet

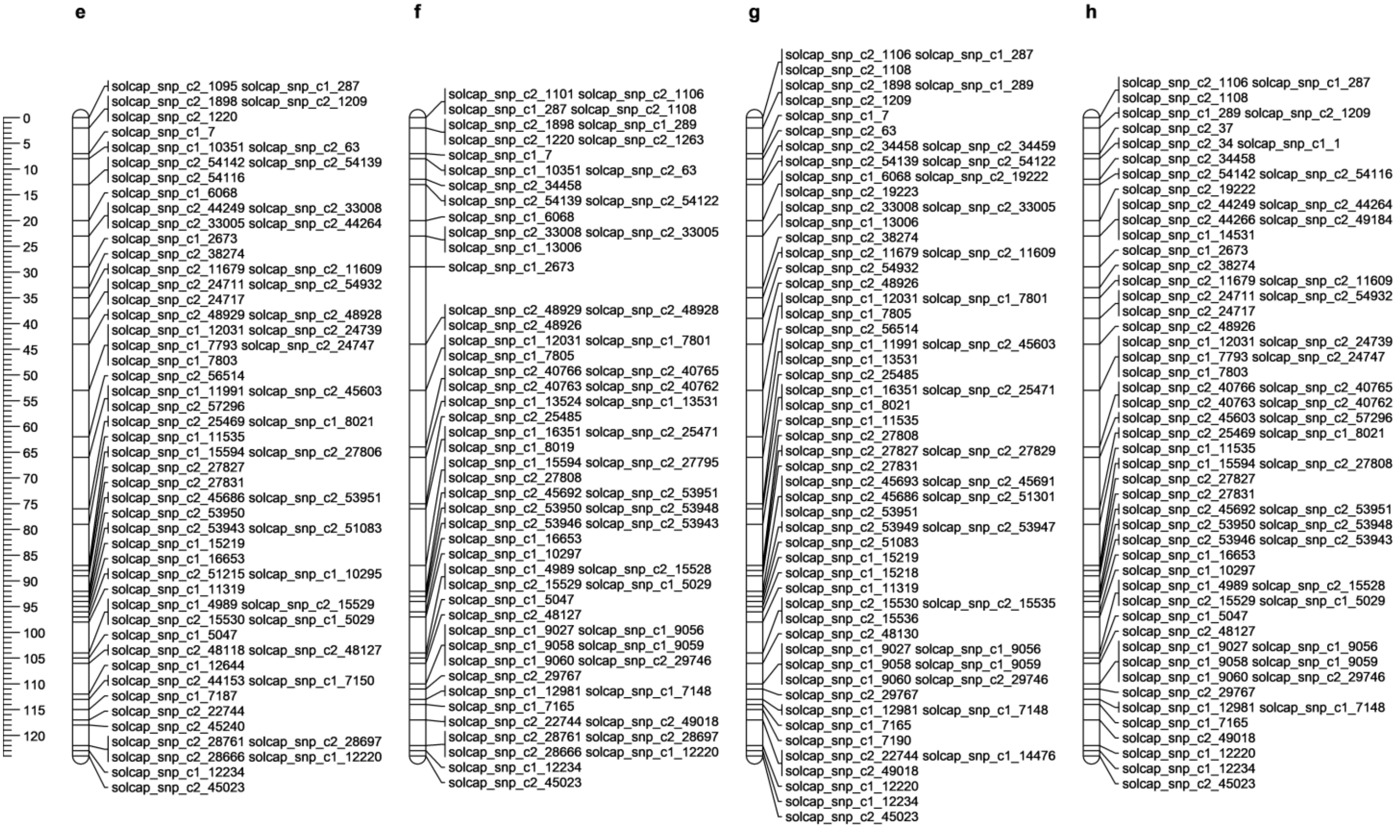

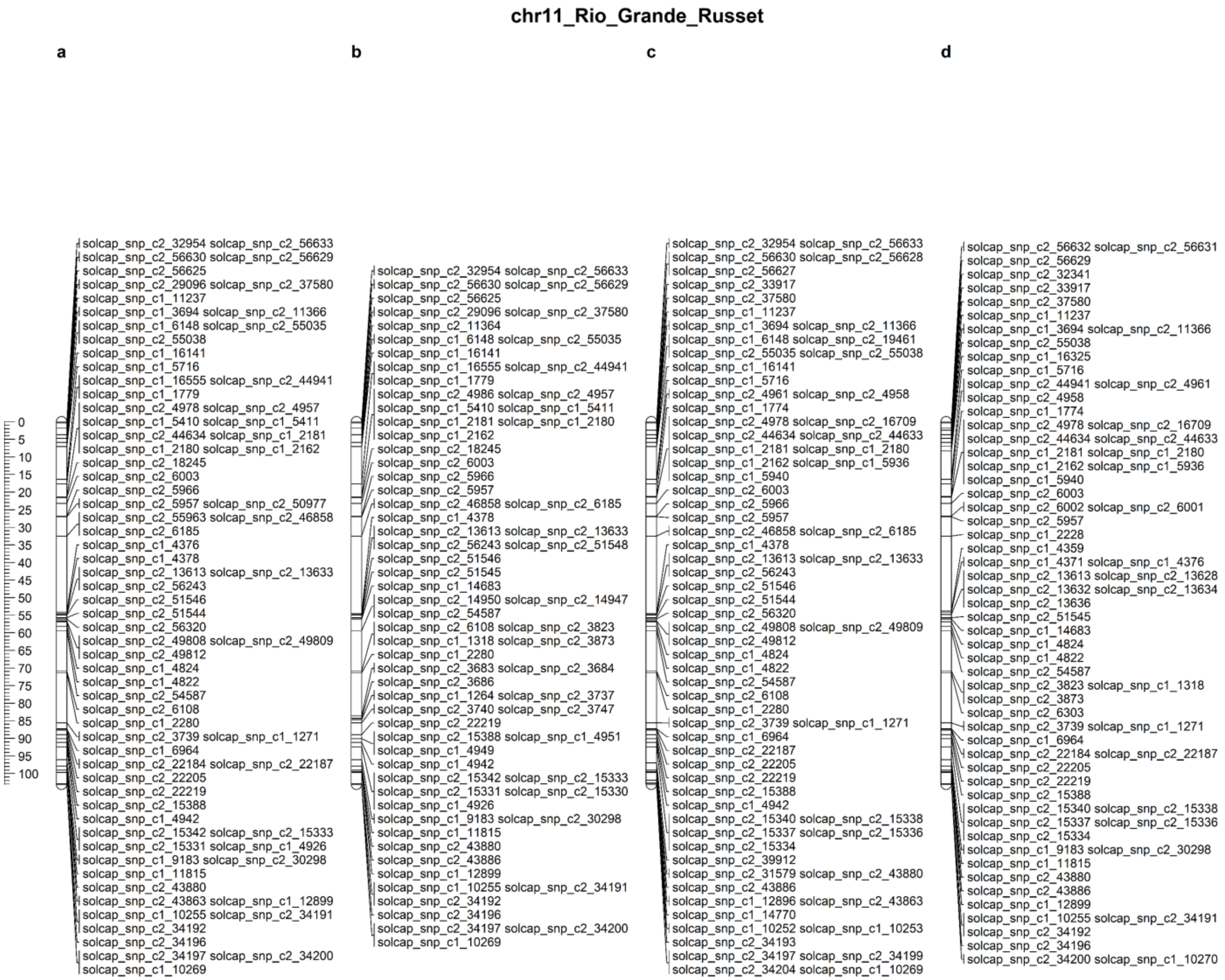

chr11_Rio_Grande_Russet
a
b
c
d
0
5
10
15
20
25
30
35
40
45
50
55
60
65
70
75
80
85
90
95
100
solcap_snp_c2_32954 solcap_snp_c2_56633
solcap_snp_c2_56630 solcap_snp_c2_56629
solcap_snp_c2_56625
solcap_snp_c2_29096 solcap_snp_c2_37580
solcap_snp_c1_11237
solcap_snp_c1_3694 solcap_snp_c2_11366
solcap_snp_c1_6148 solcap_snp_c2_55035
solcap_snp_c2_55038
solcap_snp_c1_16141
solcap_snp_c1_5716
solcap_snp_c1_16555 solcap_snp_c2_44941
solcap_snp_c1_1779
solcap_snp_c2_4978 solcap_snp_c2_4957
solcap_snp_c1_5410 solcap_snp_c1_5411
solcap_snp_c2_44634 solcap_snp_c1_2181
solcap_snp_c1_2180 solcap_snp_c1_2162
solcap_snp_c2_18245
solcap_snp_c2_6003
solcap_snp_c2_5966
solcap_snp_c2_5957 solcap_snp_c2_50977
solcap_snp_c2_55963 solcap_snp_c2_46858
solcap_snp_c2_6185
solcap_snp_c1_4376
solcap_snp_c1_4378
solcap_snp_c2_13613 solcap_snp_c2_13633
solcap_snp_c2_56243
solcap_snp_c2_51546
solcap_snp_c2_51544
solcap_snp_c2_56320
solcap_snp_c2_49808 solcap_snp_c2_49809
solcap_snp_c2_49812
solcap_snp_c1_4824
solcap_snp_c1_4822
solcap_snp_c2_54587
solcap_snp_c2_6108
solcap_snp_c1_2280
solcap_snp_c2_3739 solcap_snp_c1_1271
solcap_snp_c1_6964
solcap_snp_c2_22184 solcap_snp_c2_22187
solcap_snp_c2_22205
solcap_snp_c2_22219
solcap_snp_c2_15388
solcap_snp_c1_4942
solcap_snp_c2_15342 solcap_snp_c2_15333
solcap_snp_c2_15331 solcap_snp_c1_4926
solcap_snp_c1_9183 solcap_snp_c2_30298
solcap_snp_c1_11815
solcap_snp_c2_43880
solcap_snp_c2_43863 solcap_snp_c1_12899
solcap_snp_c1_10255 solcap_snp_c2_34191
solcap_snp_c2_34192
solcap_snp_c2_34196
solcap_snp_c2_34197 solcap_snp_c2_34200
solcap_snp_c1_10269
solcap_snp_c2_32954 solcap_snp_c2_56633
solcap_snp_c2_56630 solcap_snp_c2_56629
solcap_snp_c2_56625
solcap_snp_c2_29096 solcap_snp_c2_37580
solcap_snp_c2_11364
solcap_snp_c1_6148 solcap_snp_c2_55035
solcap_snp_c1_16141
solcap_snp_c1_16555 solcap_snp_c2_44941
solcap_snp_c1_1779
solcap_snp_c2_4986 solcap_snp_c2_4957
solcap_snp_c1_5410 solcap_snp_c1_5411
solcap_snp_c1_2181 solcap_snp_c1_2180
solcap_snp_c1_2162
solcap_snp_c2_18245
solcap_snp_c2_6003
solcap_snp_c2_5966
solcap_snp_c2_5957
solcap_snp_c2_46858 solcap_snp_c2_6185
solcap_snp_c1_4378
solcap_snp_c2_13613 solcap_snp_c2_13633
solcap_snp_c2_56243 solcap_snp_c2_51548
solcap_snp_c2_51546
solcap_snp_c2_51545
solcap_snp_c1_14683
solcap_snp_c2_14950 solcap_snp_c2_14947
solcap_snp_c2_54587
solcap_snp_c2_6108 solcap_snp_c2_3823
solcap_snp_c1_1318 solcap_snp_c2_3873
solcap_snp_c1_2280
solcap_snp_c2_3683 solcap_snp_c2_3684
solcap_snp_c2_3686
solcap_snp_c1_1264 solcap_snp_c2_3737
solcap_snp_c2_3740 solcap_snp_c2_3747
solcap_snp_c2_22219
solcap_snp_c2_15388 solcap_snp_c1_4951
solcap_snp_c1_4949
solcap_snp_c1_4942
solcap_snp_c2_15342 solcap_snp_c2_15333
solcap_snp_c2_15331 solcap_snp_c2_15330
solcap_snp_c1_4926
solcap_snp_c1_9183 solcap_snp_c2_30298
solcap_snp_c1_11815
solcap_snp_c2_43880
solcap_snp_c2_43886
solcap_snp_c1_12899
solcap_snp_c1_10255 solcap_snp_c2_34191
solcap_snp_c2_34192
solcap_snp_c2_34196
solcap_snp_c2_34197 solcap_snp_c2_34200
solcap_snp_c1_10269
solcap_snp_c2_32954 solcap_snp_c2_56633
solcap_snp_c2_56630 solcap_snp_c2_56628
solcap_snp_c2_56627
solcap_snp_c2_33917
solcap_snp_c2_37580
solcap_snp_c1_11237
solcap_snp_c1_3694 solcap_snp_c2_11366
solcap_snp_c1_6148 solcap_snp_c2_19461
solcap_snp_c2_55035 solcap_snp_c2_55038
solcap_snp_c1_16141
solcap_snp_c1_5716
solcap_snp_c2_4961 solcap_snp_c2_4958
solcap_snp_c1_1774
solcap_snp_c2_4978 solcap_snp_c2_16709
solcap_snp_c2_44634 solcap_snp_c2_44633
solcap_snp_c1_2181 solcap_snp_c1_2180
solcap_snp_c1_2162 solcap_snp_c1_5936
solcap_snp_c1_5940
solcap_snp_c2_6003
solcap_snp_c2_5966
solcap_snp_c2_5957
solcap_snp_c2_46858 solcap_snp_c2_6185
solcap_snp_c1_4378
solcap_snp_c2_13613 solcap_snp_c2_13633
solcap_snp_c2_56243
solcap_snp_c2_51546
solcap_snp_c2_51544
solcap_snp_c2_56320
solcap_snp_c2_49808 solcap_snp_c2_49809
solcap_snp_c2_49812
solcap_snp_c1_4824
solcap_snp_c1_4822
solcap_snp_c2_54587
solcap_snp_c2_6108
solcap_snp_c1_2280
solcap_snp_c2_3739 solcap_snp_c1_1271
solcap_snp_c1_6964
solcap_snp_c2_22187
solcap_snp_c2_22205
solcap_snp_c2_22219
solcap_snp_c2_15388
solcap_snp_c1_4942
solcap_snp_c2_15340 solcap_snp_c2_15338
solcap_snp_c2_15337 solcap_snp_c2_15336
solcap_snp_c2_15334
solcap_snp_c2_39912
solcap_snp_c2_31579 solcap_snp_c2_43880
solcap_snp_c2_43886
solcap_snp_c1_12896 solcap_snp_c2_43863
solcap_snp_c1_14770
solcap_snp_c1_10252 solcap_snp_c1_10253
solcap_snp_c2_34193
solcap_snp_c2_34197 solcap_snp_c2_34199
solcap_snp_c2_34204 solcap_snp_c1_10269
solcap_snp_c2_56632 solcap_snp_c2_56631
solcap_snp_c2_56629
solcap_snp_c2_32341
solcap_snp_c2_33917
solcap_snp_c2_37580
solcap_snp_c1_11237
solcap_snp_c1_3694 solcap_snp_c2_11366
solcap_snp_c2_55038
solcap_snp_c1_16325
solcap_snp_c1_5716
solcap_snp_c2_44941 solcap_snp_c2_4961
solcap_snp_c2_4958
solcap_snp_c1_1774
solcap_snp_c2_4978 solcap_snp_c2_16709
solcap_snp_c2_44634 solcap_snp_c2_44633
solcap_snp_c1_2181 solcap_snp_c1_2180
solcap_snp_c1_2162 solcap_snp_c1_5936
solcap_snp_c1_5940
solcap_snp_c2_6003
solcap_snp_c2_6002 solcap_snp_c2_6001
solcap_snp_c2_5957
solcap_snp_c1_2228
solcap_snp_c1_4359
solcap_snp_c1_4371 solcap_snp_c1_4376
solcap_snp_c2_13613 solcap_snp_c2_13628
solcap_snp_c2_13632 solcap_snp_c2_13634
solcap_snp_c2_13636
solcap_snp_c2_51545
solcap_snp_c1_14683
solcap_snp_c1_4824
solcap_snp_c1_4822
solcap_snp_c2_54587
solcap_snp_c2_3823 solcap_snp_c1_1318
solcap_snp_c2_3873
solcap_snp_c2_6303
solcap_snp_c2_3739 solcap_snp_c1_1271
solcap_snp_c1_6964
solcap_snp_c2_22184 solcap_snp_c2_22187
solcap_snp_c2_22205
solcap_snp_c2_22219
solcap_snp_c2_15388
solcap_snp_c2_15340 solcap_snp_c2_15338
solcap_snp_c2_15337 solcap_snp_c2_15336
solcap_snp_c2_15334
solcap_snp_c1_9183 solcap_snp_c2_30298
solcap_snp_c1_11815
solcap_snp_c2_43880
solcap_snp_c2_43886
solcap_snp_c1_12899
solcap_snp_c1_10255 solcap_snp_c2_34191
solcap_snp_c2_34192
solcap_snp_c2_34196
solcap_snp_c2_34200 solcap_snp_c1_10270

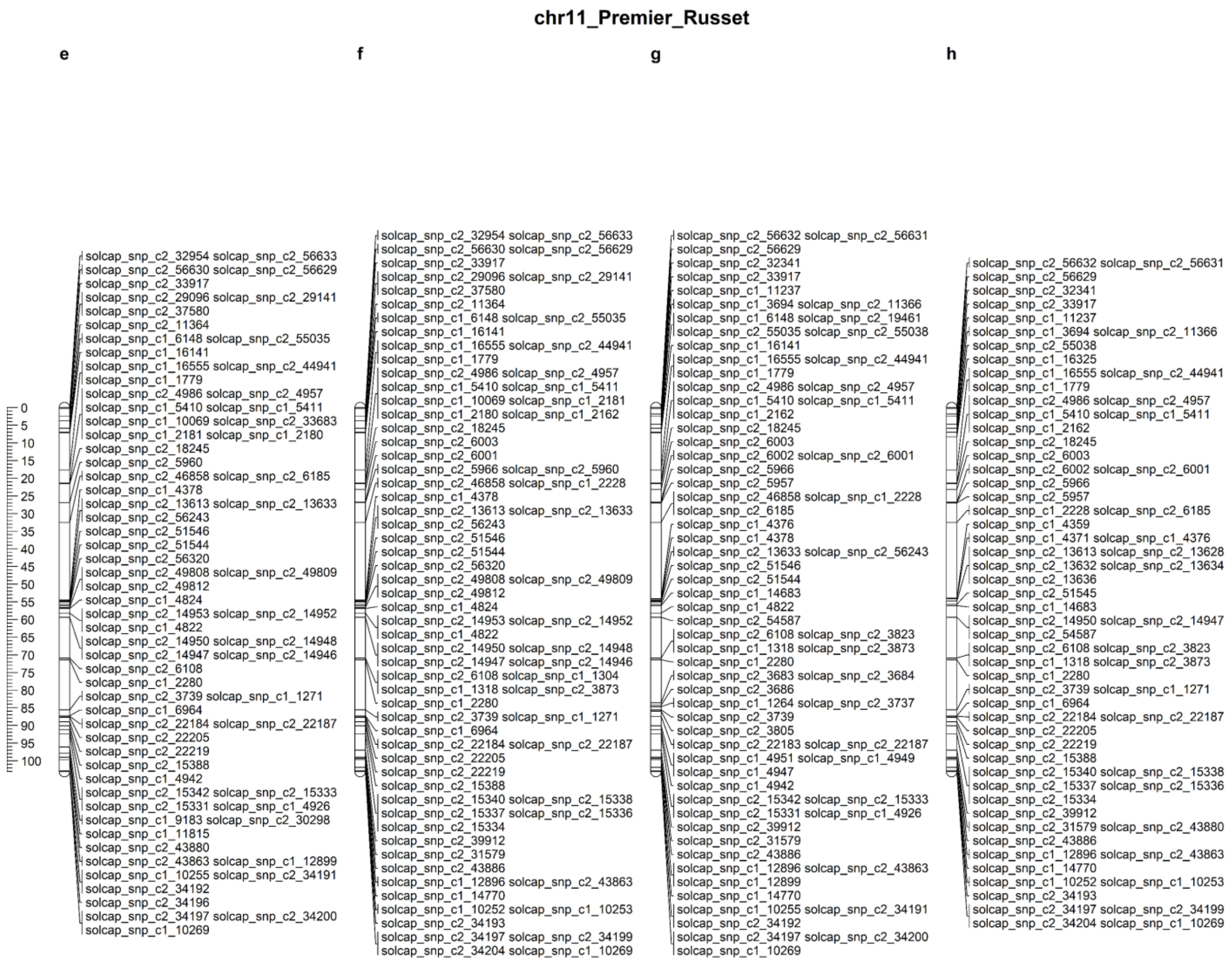
chr11_Premier_Russet
e
f
g
h
0
5
10
15
20
25
30
35
40
45
50
55
60
65
70
75
80
85
90
95
100
solcap_snp_c2_32954 solcap_snp_c2_56633
solcap_snp_c2_56630 solcap_snp_c2_56629
solcap_snp_c2_33917
solcap_snp_c2_29096 solcap_snp_c2_29141
solcap_snp_c2_37580
solcap_snp_c2_11364
solcap_snp_c1_6148 solcap_snp_c2_55035
solcap_snp_c1_16141
solcap_snp_c1_16555 solcap_snp_c2_44941
solcap_snp_c1_1779
solcap_snp_c2_4986 solcap_snp_c2_4957
solcap_snp_c1_5410 solcap_snp_c1_5411
solcap_snp_c1_10069 solcap_snp_c2_33683
solcap_snp_c1_2181 solcap_snp_c1_2180
solcap_snp_c2_18245
solcap_snp_c2_5960
solcap_snp_c2_46858 solcap_snp_c2_6185
solcap_snp_c1_4378
solcap_snp_c2_13613 solcap_snp_c2_13633
solcap_snp_c2_56243
solcap_snp_c2_51546
solcap_snp_c2_51544
solcap_snp_c2_56320
solcap_snp_c2_49808 solcap_snp_c2_49809
solcap_snp_c2_49812
solcap_snp_c1_4824
solcap_snp_c2_14953 solcap_snp_c2_14952
solcap_snp_c1_4822
solcap_snp_c2_14950 solcap_snp_c2_14948
solcap_snp_c2_14947 solcap_snp_c2_14946
solcap_snp_c2_6108
solcap_snp_c1_2280
solcap_snp_c2_3739 solcap_snp_c1_1271
solcap_snp_c1_6964
solcap_snp_c2_22184 solcap_snp_c2_22187
solcap_snp_c2_22205
solcap_snp_c2_22219
solcap_snp_c2_15388
solcap_snp_c1_4942
solcap_snp_c2_15342 solcap_snp_c2_15333
solcap_snp_c2_15331 solcap_snp_c1_4926
solcap_snp_c1_9183 solcap_snp_c2_30298
solcap_snp_c1_11815
solcap_snp_c2_43880
solcap_snp_c2_43863 solcap_snp_c1_12899
solcap_snp_c1_10255 solcap_snp_c2_34191
solcap_snp_c2_34192
solcap_snp_c2_34196
solcap_snp_c2_34197 solcap_snp_c2_34200
solcap_snp_c1_10269
solcap_snp_c2_32954 solcap_snp_c2_56633
solcap_snp_c2_56630 solcap_snp_c2_56629
solcap_snp_c2_33917
solcap_snp_c2_29096 solcap_snp_c2_29141
solcap_snp_c2_37580
solcap_snp_c2_11364
solcap_snp_c1_6148 solcap_snp_c2_55035
solcap_snp_c1_16141
solcap_snp_c1_16555 solcap_snp_c2_44941
solcap_snp_c1_1779
solcap_snp_c2_4986 solcap_snp_c2_4957
solcap_snp_c1_5410 solcap_snp_c1_5411
solcap_snp_c1_10069 solcap_snp_c1_2181
solcap_snp_c1_2180 solcap_snp_c1_2162
solcap_snp_c2_18245
solcap_snp_c2_6003
solcap_snp_c2_6001
solcap_snp_c2_5966 solcap_snp_c2_5960
solcap_snp_c2_46858 solcap_snp_c1_2228
solcap_snp_c1_4378
solcap_snp_c2_13613 solcap_snp_c2_13633
solcap_snp_c2_56243
solcap_snp_c2_51546
solcap_snp_c2_51544
solcap_snp_c2_56320
solcap_snp_c2_49808 solcap_snp_c2_49809
solcap_snp_c2_49812
solcap_snp_c1_4824
solcap_snp_c2_14953 solcap_snp_c2_14952
solcap_snp_c1_4822
solcap_snp_c2_14950 solcap_snp_c2_14948
solcap_snp_c2_14947 solcap_snp_c2_14946
solcap_snp_c2_6108 solcap_snp_c1_1304
solcap_snp_c1_1318 solcap_snp_c2_3873
solcap_snp_c1_2280
solcap_snp_c2_3739 solcap_snp_c1_1271
solcap_snp_c1_6964
solcap_snp_c2_22184 solcap_snp_c2_22187
solcap_snp_c2_22205
solcap_snp_c2_22219
solcap_snp_c2_15388
solcap_snp_c2_15340 solcap_snp_c2_15338
solcap_snp_c2_15337 solcap_snp_c2_15336
solcap_snp_c2_15334
solcap_snp_c2_39912
solcap_snp_c2_31579
solcap_snp_c2_43886
solcap_snp_c1_12896 solcap_snp_c2_43863
solcap_snp_c1_14770
solcap_snp_c1_10252 solcap_snp_c1_10253
solcap_snp_c2_34193
solcap_snp_c2_34197 solcap_snp_c2_34199
solcap_snp_c2_34204 solcap_snp_c1_10269
solcap_snp_c2_56632 solcap_snp_c2_56631
solcap_snp_c2_56629
solcap_snp_c2_32341
solcap_snp_c2_33917
solcap_snp_c1_11237
solcap_snp_c1_3694 solcap_snp_c2_11366
solcap_snp_c1_6148 solcap_snp_c2_19461
solcap_snp_c2_55035 solcap_snp_c2_55038
solcap_snp_c1_16141
solcap_snp_c1_16555 solcap_snp_c2_44941
solcap_snp_c1_1779
solcap_snp_c2_4986 solcap_snp_c2_4957
solcap_snp_c1_5410 solcap_snp_c1_5411
solcap_snp_c1_2162
solcap_snp_c2_18245
solcap_snp_c2_6003
solcap_snp_c2_6002 solcap_snp_c2_6001
solcap_snp_c2_5966
solcap_snp_c2_5957
solcap_snp_c2_46858 solcap_snp_c1_2228
solcap_snp_c2_6185
solcap_snp_c1_4376
solcap_snp_c1_4378
solcap_snp_c2_13633 solcap_snp_c2_56243
solcap_snp_c2_51546
solcap_snp_c2_51544
solcap_snp_c1_14683
solcap_snp_c1_4822
solcap_snp_c2_54587
solcap_snp_c2_6108 solcap_snp_c2_3823
solcap_snp_c1_1318 solcap_snp_c2_3873
solcap_snp_c1_2280
solcap_snp_c2_3683 solcap_snp_c2_3684
solcap_snp_c2_3686
solcap_snp_c1_1264 solcap_snp_c2_3737
solcap_snp_c2_3739
solcap_snp_c2_3805
solcap_snp_c2_22183 solcap_snp_c2_22187
solcap_snp_c1_4951 solcap_snp_c1_4949
solcap_snp_c1_4947
solcap_snp_c1_4942
solcap_snp_c2_15342 solcap_snp_c2_15333
solcap_snp_c2_15331 solcap_snp_c1_4926
solcap_snp_c2_39912
solcap_snp_c2_31579
solcap_snp_c2_43886
solcap_snp_c1_12896 solcap_snp_c2_43863
solcap_snp_c1_12899
solcap_snp_c1_14770
solcap_snp_c1_10255 solcap_snp_c2_34191
solcap_snp_c2_34192
solcap_snp_c2_34197 solcap_snp_c2_34200
solcap_snp_c1_10269
solcap_snp_c2_56632 solcap_snp_c2_56631
solcap_snp_c2_56629
solcap_snp_c2_32341
solcap_snp_c2_33917
solcap_snp_c1_11237
solcap_snp_c1_3694 solcap_snp_c2_11366
solcap_snp_c2_55038
solcap_snp_c1_16325
solcap_snp_c1_16555 solcap_snp_c2_44941
solcap_snp_c1_1779
solcap_snp_c2_4986 solcap_snp_c2_4957
solcap_snp_c1_5410 solcap_snp_c1_5411
solcap_snp_c1_2162
solcap_snp_c2_18245
solcap_snp_c2_6003
solcap_snp_c2_6002 solcap_snp_c2_6001
solcap_snp_c2_5966
solcap_snp_c2_5957
solcap_snp_c1_2228 solcap_snp_c2_6185
solcap_snp_c1_4359
solcap_snp_c1_4371 solcap_snp_c1_4376
solcap_snp_c2_13613 solcap_snp_c2_13628
solcap_snp_c2_13632 solcap_snp_c2_13634
solcap_snp_c2_13636
solcap_snp_c2_51545
solcap_snp_c1_14683
solcap_snp_c2_14950 solcap_snp_c2_14947
solcap_snp_c2_54587
solcap_snp_c2_6108 solcap_snp_c2_3823
solcap_snp_c1_1318 solcap_snp_c2_3873
solcap_snp_c1_2280
solcap_snp_c2_3739 solcap_snp_c1_1271
solcap_snp_c1_6964
solcap_snp_c2_22184 solcap_snp_c2_22187
solcap_snp_c2_22205
solcap_snp_c2_22219
solcap_snp_c2_15388
solcap_snp_c2_15340 solcap_snp_c2_15338
solcap_snp_c2_15337 solcap_snp_c2_15336
solcap_snp_c2_15334
solcap_snp_c2_39912
solcap_snp_c2_31579 solcap_snp_c2_43880
solcap_snp_c2_43886
solcap_snp_c1_12896 solcap_snp_c2_43863
solcap_snp_c1_14770
solcap_snp_c1_10252 solcap_snp_c1_10253
solcap_snp_c2_34193
solcap_snp_c2_34197 solcap_snp_c2_34199
solcap_snp_c2_34204 solcap_snp_c1_10269

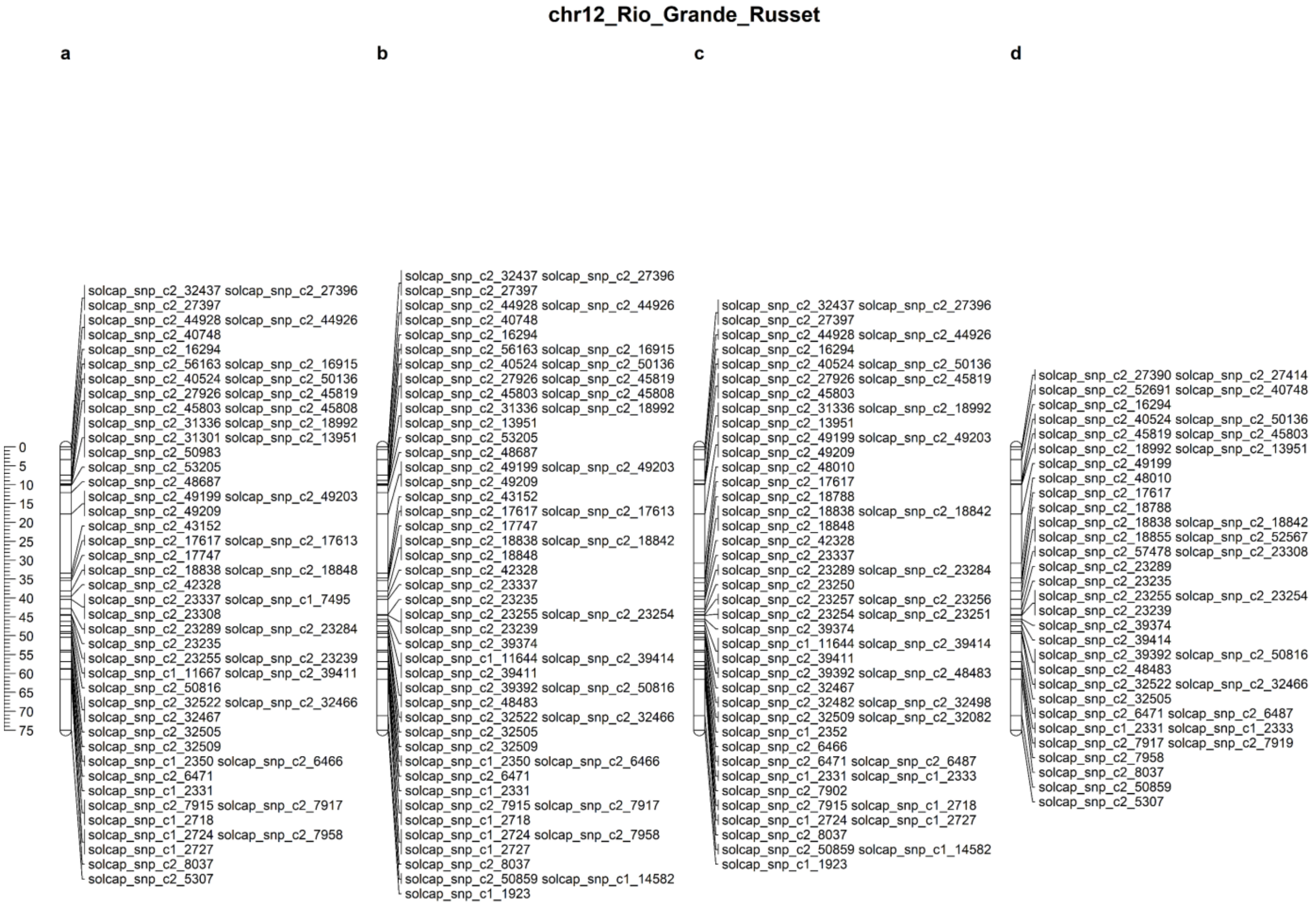
chr12_Rio_Grande_Russet
a
b
c
d
0
5
10
15
20
25
30
35
40
45
50
55
60
65
70
75
solcap_snp_c2_32437 solcap_snp_c2_27396
solcap_snp_c2_27397
solcap_snp_c2_44928 solcap_snp_c2_44926
solcap_snp_c2_40748
solcap_snp_c2_16294
solcap_snp_c2_56163 solcap_snp_c2_16915
solcap_snp_c2_40524 solcap_snp_c2_50136
solcap_snp_c2_27926 solcap_snp_c2_45819
solcap_snp_c2_45803 solcap_snp_c2_45808
solcap_snp_c2_31336 solcap_snp_c2_18992
solcap_snp_c2_31301 solcap_snp_c2_13951
solcap_snp_c2_50983
solcap_snp_c2_53205
solcap_snp_c2_48687
solcap_snp_c2_49199 solcap_snp_c2_49203
solcap_snp_c2_49209
solcap_snp_c2_43152
solcap_snp_c2_17617 solcap_snp_c2_17613
solcap_snp_c2_17747
solcap_snp_c2_18838 solcap_snp_c2_18848
solcap_snp_c2_42328
solcap_snp_c2_23337 solcap_snp_c1_7495
solcap_snp_c2_23308
solcap_snp_c2_23289 solcap_snp_c2_23284
solcap_snp_c2_23235
solcap_snp_c2_23255 solcap_snp_c2_23239
solcap_snp_c1_11667 solcap_snp_c2_39411
solcap_snp_c2_50816
solcap_snp_c2_32522 solcap_snp_c2_32466
solcap_snp_c2_32467
solcap_snp_c2_32505
solcap_snp_c2_32509
solcap_snp_c1_2350 solcap_snp_c2_6466
solcap_snp_c2_6471
solcap_snp_c1_2331
solcap_snp_c2_7915 solcap_snp_c2_7917
solcap_snp_c1_2718
solcap_snp_c1_2724 solcap_snp_c2_7958
solcap_snp_c1_2727
solcap_snp_c2_8037
solcap_snp_c2_5307
solcap_snp_c2_32437 solcap_snp_c2_27396
solcap_snp_c2_27397
solcap_snp_c2_44928 solcap_snp_c2_44926
solcap_snp_c2_40748
solcap_snp_c2_16294
solcap_snp_c2_56163 solcap_snp_c2_16915
solcap_snp_c2_40524 solcap_snp_c2_50136
solcap_snp_c2_27926 solcap_snp_c2_45819
solcap_snp_c2_45803 solcap_snp_c2_45808
solcap_snp_c2_31336 solcap_snp_c2_18992
solcap_snp_c2_13951
solcap_snp_c2_53205
solcap_snp_c2_48687
solcap_snp_c2_49199 solcap_snp_c2_49203
solcap_snp_c2_49209
solcap_snp_c2_43152
solcap_snp_c2_17617 solcap_snp_c2_17613
solcap_snp_c2_17747
solcap_snp_c2_18838 solcap_snp_c2_18842
solcap_snp_c2_18848
solcap_snp_c2_42328
solcap_snp_c2_23337
solcap_snp_c2_23235
solcap_snp_c2_23255 solcap_snp_c2_23254
solcap_snp_c2_23239
solcap_snp_c2_39374
solcap_snp_c1_11644 solcap_snp_c2_39414
solcap_snp_c2_39411
solcap_snp_c2_39392 solcap_snp_c2_50816
solcap_snp_c2_48483
solcap_snp_c2_32522 solcap_snp_c2_32466
solcap_snp_c2_32505
solcap_snp_c2_32509
solcap_snp_c1_2350 solcap_snp_c2_6466
solcap_snp_c2_6471
solcap_snp_c1_2331
solcap_snp_c2_7915 solcap_snp_c2_7917
solcap_snp_c1_2718
solcap_snp_c1_2724 solcap_snp_c2_7958
solcap_snp_c1_2727
solcap_snp_c2_8037
solcap_snp_c2_50859 solcap_snp_c1_14582
solcap_snp_c1_1923
solcap_snp_c2_32437 solcap_snp_c2_27396
solcap_snp_c2_27397
solcap_snp_c2_44928 solcap_snp_c2_44926
solcap_snp_c2_16294
solcap_snp_c2_40524 solcap_snp_c2_50136
solcap_snp_c2_27926 solcap_snp_c2_45819
solcap_snp_c2_45803
solcap_snp_c2_31336 solcap_snp_c2_18992
solcap_snp_c2_13951
solcap_snp_c2_49199 solcap_snp_c2_49203
solcap_snp_c2_49209
solcap_snp_c2_48010
solcap_snp_c2_17617
solcap_snp_c2_18788
solcap_snp_c2_18838 solcap_snp_c2_18842
solcap_snp_c2_18848
solcap_snp_c2_42328
solcap_snp_c2_23337
solcap_snp_c2_23289 solcap_snp_c2_23284
solcap_snp_c2_23250
solcap_snp_c2_23257 solcap_snp_c2_23256
solcap_snp_c2_23254 solcap_snp_c2_23251
solcap_snp_c2_39374
solcap_snp_c1_11644 solcap_snp_c2_39414
solcap_snp_c2_39411
solcap_snp_c2_39392 solcap_snp_c2_48483
solcap_snp_c2_32467
solcap_snp_c2_32482 solcap_snp_c2_32498
solcap_snp_c2_32509 solcap_snp_c2_32082
solcap_snp_c1_2352
solcap_snp_c2_6466
solcap_snp_c2_6471 solcap_snp_c2_6487
solcap_snp_c1_2331 solcap_snp_c1_2333
solcap_snp_c2_7902
solcap_snp_c2_7915 solcap_snp_c1_2718
solcap_snp_c1_2724 solcap_snp_c1_2727
solcap_snp_c2_8037
solcap_snp_c2_50859 solcap_snp_c1_14582
solcap_snp_c1_1923
solcap_snp_c2_27390 solcap_snp_c2_27414
solcap_snp_c2_52691 solcap_snp_c2_40748
solcap_snp_c2_16294
solcap_snp_c2_40524 solcap_snp_c2_50136
solcap_snp_c2_45819 solcap_snp_c2_45803
solcap_snp_c2_18992 solcap_snp_c2_13951
solcap_snp_c2_49199
solcap_snp_c2_48010
solcap_snp_c2_17617
solcap_snp_c2_18788
solcap_snp_c2_18838 solcap_snp_c2_18842
solcap_snp_c2_18855 solcap_snp_c2_52567
solcap_snp_c2_57478 solcap_snp_c2_23308
solcap_snp_c2_23289
solcap_snp_c2_23235
solcap_snp_c2_23255 solcap_snp_c2_23254
solcap_snp_c2_23239
solcap_snp_c2_39374
solcap_snp_c2_39414
solcap_snp_c2_39392 solcap_snp_c2_50816
solcap_snp_c2_48483
solcap_snp_c2_32522 solcap_snp_c2_32466
solcap_snp_c2_32505
solcap_snp_c2_6471 solcap_snp_c2_6487
solcap_snp_c1_2331 solcap_snp_c1_2333
solcap_snp_c2_7917 solcap_snp_c2_7919
solcap_snp_c2_7958
solcap_snp_c2_8037
solcap_snp_c2_50859
solcap_snp_c2_5307

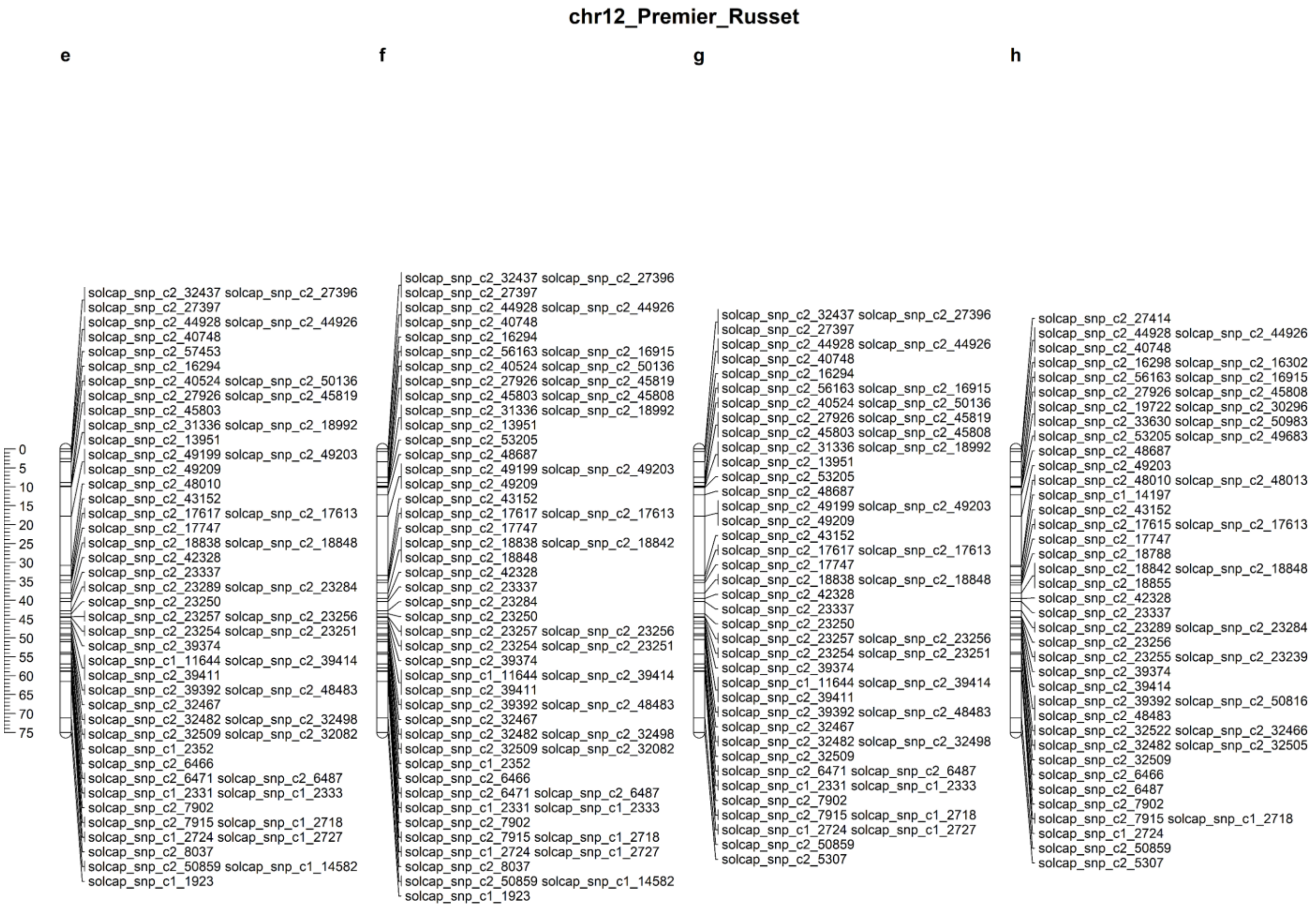
chr12_Premier_Russet
e
f
g
h
0
5
10
15
20
25
30
35
40
45
50
55
60
65
70
75
solcap_snp_c2_32437 solcap_snp_c2_27396
solcap_snp_c2_27397
solcap_snp_c2_44928 solcap_snp_c2_44926
solcap_snp_c2_40748
solcap_snp_c2_57453
solcap_snp_c2_16294
solcap_snp_c2_40524 solcap_snp_c2_50136
solcap_snp_c2_27926 solcap_snp_c2_45819
solcap_snp_c2_45803
solcap_snp_c2_31336 solcap_snp_c2_18992
solcap_snp_c2_13951
solcap_snp_c2_49199 solcap_snp_c2_49203
solcap_snp_c2_49209
solcap_snp_c2_48010
solcap_snp_c2_43152
solcap_snp_c2_17617 solcap_snp_c2_17613
solcap_snp_c2_17747
solcap_snp_c2_18838 solcap_snp_c2_18848
solcap_snp_c2_42328
solcap_snp_c2_23337
solcap_snp_c2_23289 solcap_snp_c2_23284
solcap_snp_c2_23250
solcap_snp_c2_23257 solcap_snp_c2_23256
solcap_snp_c2_23254 solcap_snp_c2_23251
solcap_snp_c2_39374
solcap_snp_c1_11644 solcap_snp_c2_39414
solcap_snp_c2_39411
solcap_snp_c2_39392 solcap_snp_c2_48483
solcap_snp_c2_32467
solcap_snp_c2_32482 solcap_snp_c2_32498
solcap_snp_c2_32509 solcap_snp_c2_32082
solcap_snp_c1_2352
solcap_snp_c2_6466
solcap_snp_c2_6471 solcap_snp_c2_6487
solcap_snp_c1_2331 solcap_snp_c1_2333
solcap_snp_c2_7902
solcap_snp_c2_7915 solcap_snp_c1_2718
solcap_snp_c1_2724 solcap_snp_c1_2727
solcap_snp_c2_8037
solcap_snp_c2_50859 solcap_snp_c1_14582
solcap_snp_c1_1923
solcap_snp_c2_32437 solcap_snp_c2_27396
solcap_snp_c2_27397
solcap_snp_c2_44928 solcap_snp_c2_44926
solcap_snp_c2_40748
solcap_snp_c2_16294
solcap_snp_c2_56163 solcap_snp_c2_16915
solcap_snp_c2_40524 solcap_snp_c2_50136
solcap_snp_c2_27926 solcap_snp_c2_45819
solcap_snp_c2_45803 solcap_snp_c2_45808
solcap_snp_c2_31336 solcap_snp_c2_18992
solcap_snp_c2_13951
solcap_snp_c2_53205
solcap_snp_c2_48687
solcap_snp_c2_49199 solcap_snp_c2_49203
solcap_snp_c2_49209
solcap_snp_c2_43152
solcap_snp_c2_17617 solcap_snp_c2_17613
solcap_snp_c2_17747
solcap_snp_c2_18838 solcap_snp_c2_18842
solcap_snp_c2_18848
solcap_snp_c2_42328
solcap_snp_c2_23337
solcap_snp_c2_23284
solcap_snp_c2_23250
solcap_snp_c2_23257 solcap_snp_c2_23256
solcap_snp_c2_23254 solcap_snp_c2_23251
solcap_snp_c2_39374
solcap_snp_c1_11644 solcap_snp_c2_39414
solcap_snp_c2_39411
solcap_snp_c2_39392 solcap_snp_c2_48483
solcap_snp_c2_32467
solcap_snp_c2_32482 solcap_snp_c2_32498
solcap_snp_c2_32509 solcap_snp_c2_32082
solcap_snp_c1_2352
solcap_snp_c2_6466
solcap_snp_c2_6471 solcap_snp_c2_6487
solcap_snp_c1_2331 solcap_snp_c1_2333
solcap_snp_c2_7902
solcap_snp_c2_7915 solcap_snp_c1_2718
solcap_snp_c1_2724 solcap_snp_c1_2727
solcap_snp_c2_8037
solcap_snp_c2_50859 solcap_snp_c1_14582
solcap_snp_c1_1923
solcap_snp_c2_32437 solcap_snp_c2_27396
solcap_snp_c2_27397
solcap_snp_c2_44928 solcap_snp_c2_44926
solcap_snp_c2_40748
solcap_snp_c2_16294
solcap_snp_c2_56163 solcap_snp_c2_16915
solcap_snp_c2_40524 solcap_snp_c2_50136
solcap_snp_c2_27926 solcap_snp_c2_45819
solcap_snp_c2_45803 solcap_snp_c2_45808
solcap_snp_c2_31336 solcap_snp_c2_18992
solcap_snp_c2_13951
solcap_snp_c2_53205
solcap_snp_c2_48687
solcap_snp_c2_49199 solcap_snp_c2_49203
solcap_snp_c2_49209
solcap_snp_c2_43152
solcap_snp_c2_17617 solcap_snp_c2_17613
solcap_snp_c2_17747
solcap_snp_c2_18838 solcap_snp_c2_18848
solcap_snp_c2_42328
solcap_snp_c2_23337
solcap_snp_c2_23250
solcap_snp_c2_23257 solcap_snp_c2_23256
solcap_snp_c2_23254 solcap_snp_c2_23251
solcap_snp_c2_39374
solcap_snp_c1_11644 solcap_snp_c2_39414
solcap_snp_c2_39411
solcap_snp_c2_39392 solcap_snp_c2_48483
solcap_snp_c2_32467
solcap_snp_c2_32482 solcap_snp_c2_32498
solcap_snp_c2_32509
solcap_snp_c2_6471 solcap_snp_c2_6487
solcap_snp_c1_2331 solcap_snp_c1_2333
solcap_snp_c2_7902
solcap_snp_c2_7915 solcap_snp_c1_2718
solcap_snp_c1_2724 solcap_snp_c1_2727
solcap_snp_c2_50859
solcap_snp_c2_5307
solcap_snp_c2_27414
solcap_snp_c2_44928 solcap_snp_c2_44926
solcap_snp_c2_40748
solcap_snp_c2_16298 solcap_snp_c2_16302
solcap_snp_c2_56163 solcap_snp_c2_16915
solcap_snp_c2_27926 solcap_snp_c2_45808
solcap_snp_c2_19722 solcap_snp_c2_30296
solcap_snp_c2_33630 solcap_snp_c2_50983
solcap_snp_c2_53205 solcap_snp_c2_49683
solcap_snp_c2_48687
solcap_snp_c2_49203
solcap_snp_c2_48010 solcap_snp_c2_48013
solcap_snp_c1_14197
solcap_snp_c2_43152
solcap_snp_c2_17615 solcap_snp_c2_17613
solcap_snp_c2_17747
solcap_snp_c2_18788
solcap_snp_c2_18842 solcap_snp_c2_18848
solcap_snp_c2_18855
solcap_snp_c2_42328
solcap_snp_c2_23337
solcap_snp_c2_23289 solcap_snp_c2_23284
solcap_snp_c2_23256
solcap_snp_c2_23255 solcap_snp_c2_23239
solcap_snp_c2_39374
solcap_snp_c2_39414
solcap_snp_c2_39392 solcap_snp_c2_50816
solcap_snp_c2_48483
solcap_snp_c2_32522 solcap_snp_c2_32466
solcap_snp_c2_32482 solcap_snp_c2_32505
solcap_snp_c2_32509
solcap_snp_c2_6466
solcap_snp_c2_6487
solcap_snp_c2_7902
solcap_snp_c2_7915 solcap_snp_c1_2718
solcap_snp_c1_2724
solcap_snp_c2_50859
solcap_snp_c2_5307
