# Supplementary material for: Linkage and QTL mapping for tuber shape and specific gravity in a tetraploid mapping population of potato representing the russet market class
Source: BMC Plant Biol. 2021 Nov 3;21:507. doi: 10.1186/s12870-021-03265-2 (PMC8565078; doi:10.1186/s12870-021-03265-2)
Supplement: Supplementary file 9 — Additional file 9: Supplementary Table S3 Summary of the BLUP datasets (DOCX 16.1 KB). ** Supplementary Table 3 is uploaded as a separate Microsoft Word file. [file 12870_2021_3265_MOESM9_ESM.docx]

**Additional file 11: Supplementary Table 3. Summary of the BLUP datasets**

| **Tuber Shape (TS)** | | | | | | | |
| --- | --- | --- | --- | --- | --- | --- | --- |
| BLUP datasets ^a^ |  | Description | |  | | Total # of BLUPs | |
| *TS_clo* |  | BLUPs of pooling phenotype data across all two-years and two locations | |  | | 182 | |
| *TS_clo_ID* |  | BLUPs of interaction between clone and Idaho (location) | |  | | 181 | |
| *TS_clo_NC* |  | BLUPs of interaction between clone and North Carolina (location) | |  | | 180 | |
| *TS_clo_2010* |  | BLUPs of interaction between clone and 2010 (year) | |  | | 178 | |
| *TS_clo_2011* |  | BLUPs of interaction between clone and 2011 (year) | |  | | 173 | |
| *TS_clo_ID_2010* |  | BLUPs of interaction among clone, 2010 (year), and Idaho (location) | |  | | 178 | |
| *TS_clo_ID_2011* |  | BLUPs of interaction among clone, 2011 (year), and Idaho (location) | |  | | 165 | |
| *TS_clo_NC_2010* |  | BLUPs of interaction among clone, 2010 (year), and North Carolina (location) | |  | | 176 | |
| *TS_clo_NC_2011* |  | BLUPs of interaction among clone, 2011 (year), and North Carolina (location) | |  | | 166 | |
| **Specific Gravity (SG)** | | | | | | | |
| BLUP datasets ^a, c^ |  | Description | |  | | Total # of  BLUPs ^b^ | |
| *SG_clo* |  | | BLUPs of pooling phenotype data across all two-years and three locations | |  | | 183 |
| *SG_clo_ID* |  | | BLUPs of interaction between clone and Idaho (location) | |  | | 182 |
| *SG_clo_NC* |  | | BLUPs of interaction between clone and North Carolina (location) | |  | | 180 |
| *SG_clo_MN* |  | | BLUPs of interaction between clone and Minnesota (location) | |  | | 168 |
| *SG_clo_ID_2010* |  | | BLUPs of interaction among clone, 2010 (year), and Idaho (location) | |  | | 179 |
| *SG_clo_ID_2011* |  | | BLUPs of interaction among clone, 2011 (year), and Idaho (location) | |  | | 165 |
| *SG_clo_NC_2010* |  | | BLUPs of interaction among clone, 2010 (year), and North Carolina (location) | |  | | 176 |
| *SG_clo_NC_2011* |  | | BLUPs of interaction among clone, 2011 (year), and North Carolina (location) | |  | | 166 |
| *SG_clo_MN_2011* |  | | BLUPs of interaction among clone, 2011 (year), and Minnesota (location) | |  | | 168 |
|  |  | |  | |  | |  |

^a^ BLUP data abbreviations: tuber shape (*TS*), specific gravity (*SG*), a genetic effect of clones (*clo*), Idaho (*ID*), North Carolina (*NC*), Minnesota (*MN*) location effects, 2010 (*2010*) and 2011 (*2011*) year effects

^b^ Due to missing values, while phenotyping, each raw phenotype dataset has a different number of observations for the two traits resulting in the BLUP datasets composed of various amounts of components

^c^ The variance of clone × year was zero for specific gravity resulting in the absence of the BLUP datasets of the interactions between clone and year (e.g., SG_clo_2010 and SG_clo_2011).
